# Environmentally Friendly Approach to Knoevenagel Condensation of Rhodanine in Choline Chloride: Urea Deep Eutectic Solvent and QSAR Studies on Their Antioxidant Activity

**DOI:** 10.3390/molecules23081897

**Published:** 2018-07-29

**Authors:** Maja Molnar, Harshad Brahmbhatt, Vesna Rastija, Valentina Pavić, Mario Komar, Maja Karnaš, Jurislav Babić

**Affiliations:** 1 Faculty of Food Technology Osijek, Josip Juraj Strossmayer University of Osijek, Franje Kuhaca 20, 31000 Osijek, Croatia; brahmbhattharshad@hotmail.com (H.B.); mkomar@ptfos.hr (M.K.); jbabic@ptfos.hr (J.B.); 2 Faculty of Agriculture, Josip Juraj Strossmayer University of Osijek, Vladimira Preloga 1, 31000 Osijek, Croatia; Vesna.Rastija@pfos.hr (V.R.); Maja.Karnas@pfos.hr (M.K.); 3 Department of Biology, Josip Juraj Strossmayer University of Osijek, Cara Hadrijana 8/A, 31000 Osijek, Croatia; vpavic@biologija.unios.hr

**Keywords:** rhodanine, deep eutectic solvents, Knoevenagel condensation, antioxidant activity, QSAR, molecular docking, tyrosinase

## Abstract

A series of rhodanine derivatives was synthesized in the Knoevenagel condensation of rhodanine and different aldehydes using choline chloride:urea (1:2) deep eutectic solvent. This environmentally friendly and catalyst free approach was very effective in the condensation of rhodanine with commercially available aldehydes, as well as the ones synthesized in our laboratory. All rhodanine derivatives were subjected to 1,1-diphenyl-2-picrylhydrazyl radical (DPPH) scavenging activity investigation and quantitative structure-activity relationship (QSAR) studies were performed to elucidate their structure-activity relationship. The best multiple linear QSAR model demonstrate a stability in the internal validation and Y-randomization (*R*^2^ = 0.81; *F* = 24.225; *Q*^2^_loo_ = 0.72; *R*^2^_Yscr_ = 0.148). Sphericity of the molecule, ratio of symmetric atoms enhanced atomic mass along the principle axes in regard to total number of atoms in molecule, and 3D distribution of the atoms higher electronegativity (O, N, and S) in molecules are important characteristic for antioxidant ability of rhodanine derivatives. Molecular docking studies were carried out in order to explain in silico antioxidant studies, a specific protein tyrosine kinase (2HCK). The binding interactions of the most active compound have shown strong hydrogen bonding and van der Waals interactions with the target protein.

## 1. Introduction

Deep eutectic solvents (DESs) are often characterized as environmentally friendly and acceptable, due to their low vapor pressure, non-toxic properties, reusability, and cheapness [1,2]. DESs are formed by the interaction of different hydrogen bond acceptors (HBA), like quaternary ammonium salts, and hydrogen bond donors (HBD), like urea, carboxylic acids, sugars or amides [1]. They have been proven to be efficient in many applications, such as organic synthesis, extraction, electrochemistry, etc. [3,4,5]. Their application in organic synthesis includes synthesis of different heterocycles [6,7,8,9,10], esterification [11], aldol reactions [12], halogenation [13] as well as *N*-acetylation of amines [14], synthesis of mercapto quinazolinones [15], and Schiff bases [16], all performed by our group. Knoevenagel condensation in deep eutectic solvents has been studied by Keshavarzipour and Tavakol [17], indicating that DESs could be a very useful tool in this type of condensation. Knoevenagel condensation has been employed in the synthesis of numerous heterocyclic compounds, and is one of the basic reactions in organic chemistry. Many different conditions, catalysts and solvent were studied in this regard. Yarovenko et al. [18] studied Knoevenagel condensation of rhodanine with various aldehydes in different solvents and with different catalysts. They found that methanol as a solvent and ethylenediammonium diacetate at ambient temperature gave high yield of the product, sodium acetate in acetic acid or acetic anhydride gave moderate yields, while ethanol and piperidine as a catalyst gave no product.

*N*-Substituted 5-arylidenerhodanines are usually synthesized by the Knoevenagel condensation of the *N*-substituted rhodanine with various aldehydes, conventionally or using microwaves in one-pot two-step process [19]. Nitche and Klein synthesized *N*-substituted rhodanines from amines under microwaves using water as a solvent [20], while Azizi et al. synthesized arylidene rhodanine derivatives by condensation of primary amines, aldehydes, ethyl chloroacetate, and carbon disulphide in polyethylene glycol (PEG) at room temperature or with ultrasonic irradiation [21]. Suresh and Sandhu performed the Knoevenagel condensation of rhodanines using ionic liquids under ultrasound irradiation [22].

Rhodanines have been reported to possess antibacterial [23], antiviral [24], antimalarial [25], antifungal [26], and antitumor activity [27]. Antioxidants are the bioactive chemical compounds which fight against oxidative stress by neutralizing the free radicals, such as reactive oxygen species (ROS) and thereby prevent many diseases. Excessive generation of the reactive oxygen species has been shown to activate protein kinases. The small molecules may function through directly inhibiting the catalytic activity of the kinase by interfering with the binding of adenosine triphosphate (ATP) or substrates [28].

Therefore, the aim of this research is to employ a novel green approach in Knoevenagel condensation of rhodanine derivatives as potential antioxidants.

Furthermore, in order to signify the importance of structural and chemical attributes for the antioxidant activity of 5-arylidenerhodanines, and to improve further research on the mechanisms of action of these compounds against ROS, quantitative structure-activity relationship (QSAR) analysis is performed. Interaction of 5-arylidenerhodanines and haematopoietic cell tyrosine kinase (Hck) in silico has evaluated by molecular docking based on binding mode of quercetin as inhibitor.

## 2. Results and Discussion

### 2.1. Synthesis

**Scheme 1:**
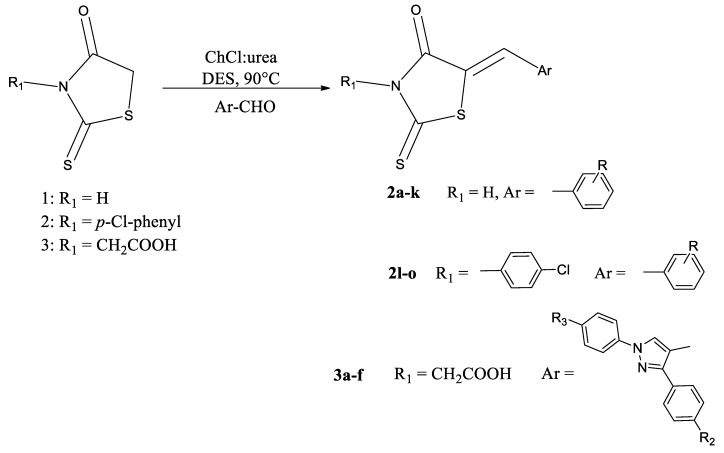
Synthesis of rhodanine derivatives via Knoevenagel condensation (R: **2a**: 2-OCH_3_; **2b**: 4-N(CH_3_)_2_; **2c**: 3-OCH_3_; **2d**: 3,4,5-(OCH_3_)_3_; **2e**: 3,4-(OH)_2_; **2f**: 3-OCH_3_, 4-OH; **2g**: 2-OH; **2h**: H; **2i**: 4-pyridyl; **2j**: 2,5-(OCH_3_)_2_; **2k**: 4-OH; **2l**: 3-OCH_3_, 4-OH; **2m**: 4-N(CH_3_)_2_; **2n**: 4-OH; **2o**: 2,5-(OCH_3_)_2_; **3a**: R_1_ = CH_2_COOH, R_2_ = CH_3_, R_3_ = CH_3_; **3b**: R_1_ = CH_2_COOH, R_2_ = F, R_3_ = CH_3_; **3c**: R_1_ = CH_2_COOH, R_2_ = OCH_3_, R_3_ = CH_3_; **3d**: R_1_ = CH_2_COOH, R_2_ = Cl, R_3_ = CH_3_; **3e**: R_1_ = CH_2_COOH, R_2_ = Br, R_3_ = CH_3_; **3f**: R_1_ = CH_2_COOH, R_2_ = I, R_3_ = CH_3_).

Synthesis of the target compounds (Scheme 1) was performed from rhodanine and corresponding aldehydes, commercially available and synthesized ones, as the starting compounds. The deep eutectic solvent formed of the choline chloride and urea was found to be very suitable for this type of condensation, since no catalyst was required, and the yields of the compounds were moderate to excellent. Besides its green character, this method is characterized by a simplicity of post synthetic procedures, which includes the addition of water into reaction mixture followed by product precipitation. A formation of the condensed rhodanine derivatives was confirmed by nuclear magnetic resonance (NMR) spectroscopy, where there is no rhodanine –CH_2_– peak around 4.2 ppm noticed in synthesized compounds. Furthermore, new peaks appear in the synthesized compounds and are attributed to aromatic protons of the substituents 7.07–7.50 ppm, as well as =CH– proton around 7.7 ppm. Unsubstituted –NH rhodanine group in the compounds **2a**–**k** is characterized by the peak around 13.55 ppm.

We have also investigated a possibility to recycle and reuse DES in several cycles for the same reaction. As a model reaction we utilized a reaction between rhodanine and 4-dimethylaminobenzaldehyde. After the isolation of desired product, water was evaporated from DES and the same DES was used again for the same reaction. We performed three cycles of the above described procedure and the results are presented in Table 1. These results confirmed the fact stated before, that DESs, besides other characteristics, owe their green character to the fact that they can be effectively reused.

### 2.2. Antioxidant Activity

Compounds (**2a**–**3f**) were investigated for their antioxidant activity expressed as % 1,1-diphenyl-2-picrylhydrazyl radical (DPPH) scavenging activity. Data presented in Table 2 show that the substituents on 5-arylidenerhodanines have a great influence on the antioxidant activity. Benzylidene derivatives of the 5-arylidenerhodanines (**2a**–**2o**) manifested enhanced antioxidant activity, compared to 1,3-diphenyl-1*H*-pyrazole derivatives (**3a**–**3f**). Benzylidene derivative **2e**, with two hydroxyl groups in 3,4-postion on phenyl ring, showed the highest antioxidant activity of 71.2%. Substitution of the 3-OH group with methoxy group decreased the activity of compound **2f** (39.2%). Furthermore, when a starting rhodanine compounds **1**–**3** are compared to the substituted ones, it is noticeable that the substitution of rhodanine **1** in position 5 (compounds **2a**–**k**) enhances an antioxidant activity in most cases. For derivatives (**2l**–**o**) of compound **2**, as well as compounds **3a**–**f**, derivatives of compound **3**, it is noticed that substitution of a starting compounds in position 5 decreases the antioxidant activity.

### 2.3. QSAR Models

The best QSAR model for the antioxidant activity of 21 rhodanine derivatives was obtained by the combination of DRAGON and ADMEWORKS descriptors:

log % DPPH = 18.086 − 9.117 *SPH* − 5.494 *Mor24e* − 44.94 *G2m* (1)

where *SPH* is a sphericity of molecule; *Mor24e* is 3D-MoRSE descriptor reflects the contribution of the 3D distribution of Sanderson electronegativity; and *G2m* is the 2nd component symmetry directional WHIM index.

Fitting criteria:

*R*^2^ = 0.81; *R*^2^_adj_ = 0.777; *Kxx* = 0.264; Δ*K* = 0.05; *RMSE*_tr_ = 0.342; *MAE*_tr_ = 0.277; *RSS*_tr_ = 2.46; *CCC*_tr_ = 0.895; *s* = 0.38; *F* = 24.225

Internal validation criteria:

*Q*^2^_loo_ = 0.72; *RMSE*_cv_ = 0.416; *MAE*_cv_ = 0.348; *CCC*_cv_ = 0.843; *PRESS*_cv_ = 3.629

Robustness:

*R*^2^_Yscr_ = 0.148; *Q*^2^_Yscr_ = −0.351; *RMSE*_AVYscr_ = 0.724

Descriptor values, experimental values and values of the antioxidant activity calculated by model, as leverage of compounds (HAT values) are given in Supplementary Material 1 (SM 1).

**Figure 1:**
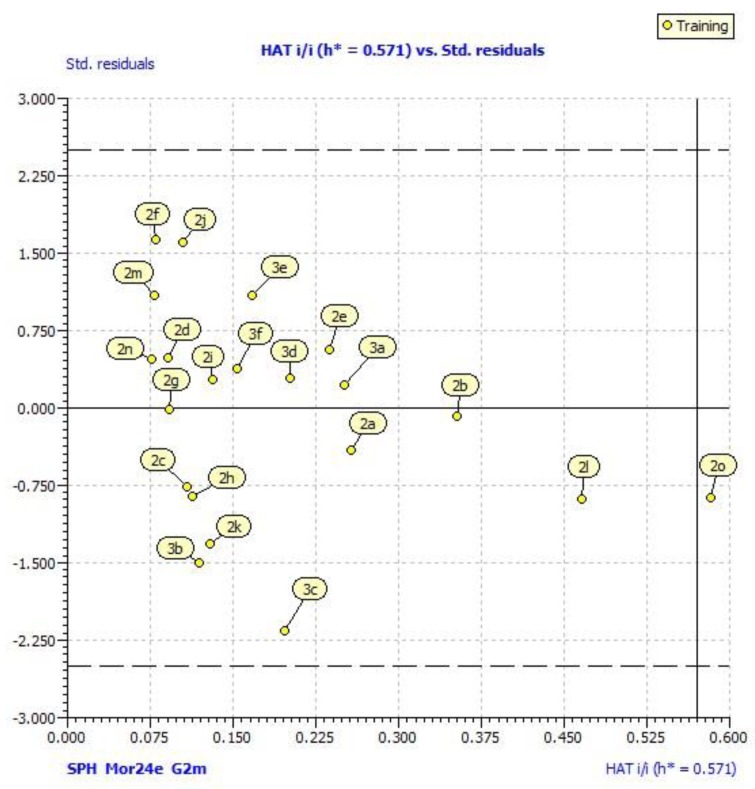
Williams plot of the applicability domain of the quantitative structure-activity relationship (QSAR) model for antioxidant activity calculated by model (1).

**Figure 2:**
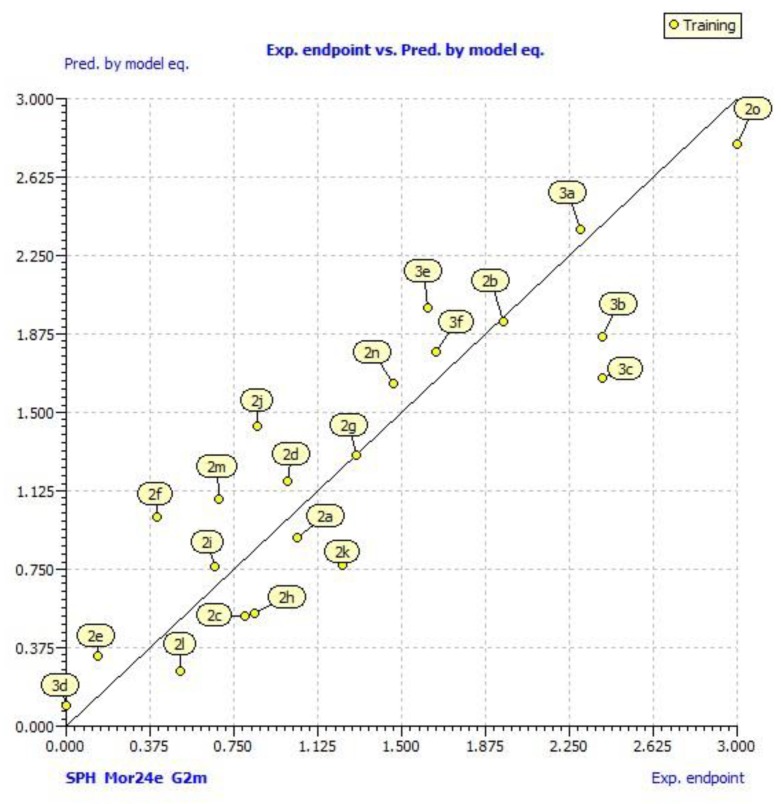
Observed versus predicted log DPPH for the 21 derivatives of rhodanine calculated by model (1).

The model satisfied fitting and internal validation criteria: *R*^2^ ≥ 0.60 (the closer *R*^2^ values are to unity, the more similar calculated values are to the experimental ones); *CCC ≥* 0.85; *RMSE* and *MAE* close to zero and *RMSE*_tr_ < *RMSE*_cv_ (Table 3). Also, larger *F* statistic and lower standard deviation means that the model is more significant. Robustness of obtained QSAR models is affirmed by *R^2^_y_*scr and *Q*^2^_y_scr values < 0.2, as *R*^2^_y_scr > *Q*^2^_y_scr [29]. Low collinearity between descriptors included in models is verified by the low values of *Kxx* and values of Δ*K* ≥ 0.05. Williams plots for the same models reveal no outliers, but compound **2o**, which is practically inactive (% DPPH = 0.1), is outside of applicability domain (Figure 1). Its leverage (*HAT* = 0.583) is greater than the warning leverage (h* = 0.571), therefore predicted value of compound **2o** must be used with a great care. A scatter plot of the experimentally obtained antioxidant activity by the model (1) is shown in Figure 2.

Due to a small number of molecules in the dataset it was not possible to make an external validation. Despite the fact that the model is not predictive, included molecular descriptors may relieve in elucidation of the important physicochemical and structural requirements for the antioxidant activity of rhodanine derivatives. Sphericity (*SPH)* is a geometrical descriptor. The sphericity index varies from zero for flat molecules, to unity for totally spherical molecules [30]. According to the negative coefficient of *SPH* in Equation (1), the more spherical the molecule, the higher is the value for this descriptor, which results in a higher antioxidant ability. The 3D-MoRSE (Molecular Representation of Structures based on Electronic diffraction) descriptor *Mor24e* reflects the contribution of 3D distribution of Sanderson electronegativity (*e*) at a scattering parameter *s* = 23 Å^−1^ [31]. According to the model (1), it is expected that the increased values of *Mor24e* indicate the higher antioxidant activity. This descriptor is extremely sensitive to the position of the atoms of higher electronegativity (O, N, and S) in molecules. According to the negative coefficients of *Mor24e* in Equation (1), compounds with the higher antioxidant activity have enhanced positive values of this descriptor.

The third variable in the model (1) is WHIM descriptors (Weighted Holistic Invariant Molecular descriptors) *G2m* (2nd component symmetry directional WHIM index/weighted by mass). WHIM descriptors (Weighted Holistic Invariant Molecular descriptors) are geometrical descriptors based on the statistical indices, calculated on the projections of the atoms along principal axes [32]. *G2m* captures the 3D information about symmetry along the principal axes. Molecular symmetry is evaluated on the basis of the number of symmetric atoms with respect to the molecule centre, and the number of unsymmetrical atoms. Considering the negative coefficient of *G2m* in Equation (1), it may be concluded that molecules with symmetrical distribution of atoms higher atomic mass along the second axes have better antioxidant ability. Benzylidene derivatives of 5-arylidenerhodanines (**2a**–**2o**) have a smaller total number of atoms and more symmetrical molecular shape compared to the 1,3-diphenyl-1*H*-pyrazole derivatives (**3a**–**3f**). Consequently, benzylidene derivatives (**2a**–**2o**) have higher values of *G2m* and the increased antioxidant activity (SM 1).

### 2.4. Molecular Docking Study

Molecular docking studies provide a virtual screening of different molecules as a potential ligands to predict their ability to interact with the given target candidates. For the present study we have chosen the hematopoietic cell tyrosine kinase (PDB ID: 2HCK) in the complex with quercetin as a target molecule [33]. Molecule with the best determined antioxidant activity (**2e**) was chosen as a ligand. Docking score and energy of the interactions between protein residue and ligand are tabulated in Table 3.

**Figure 3:**
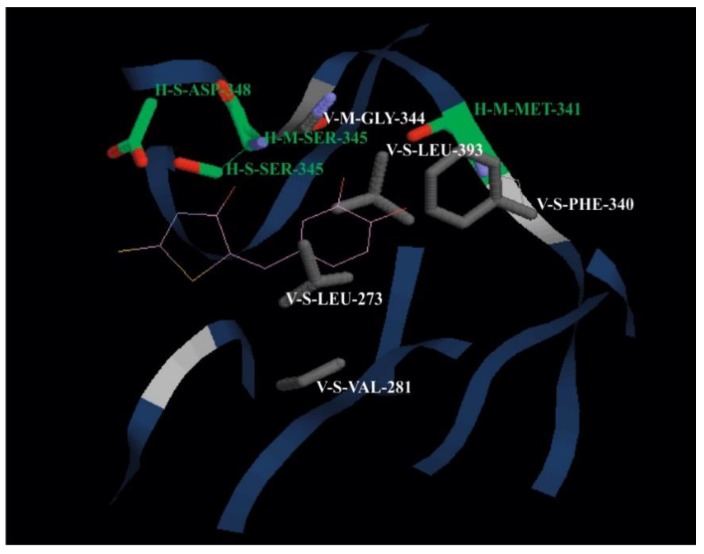
Interactions of the most active compound **2e** with the nearby amino acid group of the tyrosine kinase Hck. The following rows show the interactions between the active compounds and the interacting residue groups. H and V indicate the interaction types (H bond and van der Waals interactions, respectively); M and S indicate the main chain and the side chain of the interacting residue, respectively. The hydrogen-bondings are coloured in green and van der Waals (white) if the energy ≤−2.5. Docking pose of ligand is pink, where yellow denotes sulphur atoms and blue nitrogen atom.

**Figure 4:**
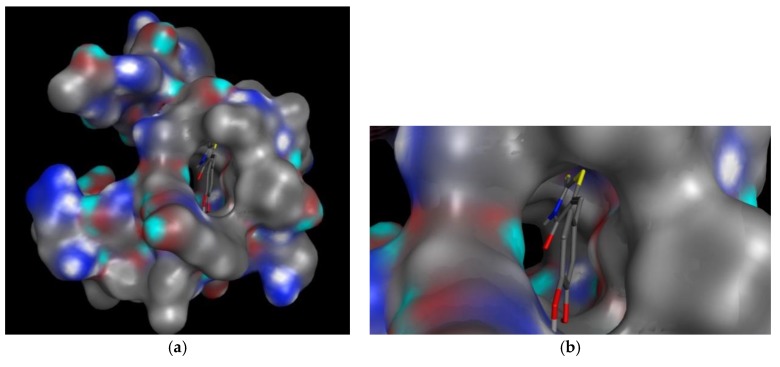
Surface representation of: (**a**) The entire tyrosine kinase hematopoietic cell kinase (Hck); and (**b**) a segment of tyrosine kinase Hck active site with docked compound **2e**.

Figure 3 illustrates the interaction of compound **2e** with 2HCK in binding site, while Figure 4 shows the cavity of hematopoietic cell tyrosine kinase with the docked compound **2e**. M and S indicate the main chain and the side chain of the interacting residue, respectively.

Molecular docking confirmed that the (*Z*)-5-(3,4-dihydroxybenzylidene)-2-thioxothiazolidin-4-one (**2e**) forms the key interactions, the four H bond with Met 341, Ser 345 (one with the main chain, and the other one with the side chain), and with Asp 348. Compound **2e** forms the van der Waals interactions with Leu 273, Val 281, Phe 340, Gly 344, Ser 345, and Leu 393. Interactions of the molecule **2e** match with the interactions of quercetin in the binding site. Quercetin forms H bonds with Val 281, Leu 273, Gly 344, Leu 393, Met 341, and Ser 345 located at the C-terminal lobe of tyrosine kinase Hck [33]. The hematopoietic cell kinase (Hck) is a member of the tyrosine-protein kinase (SRC) family of the cytoplasmic tyrosine kinases (SFKs), and is expressed in the cells of the myeloid and B-lymphocyte cell lineages. Excessive HcK activation is associated with several types of leukemia, and enhances the cell proliferation [34].

## 3. Materials and Methods

### 3.1. General

All chemicals were purchased from the commercial suppliers. Melting points of all compounds were determined with the Electrotermal (Rochford, UK) melting point apparatus and were used without correction. Thin layer chromatography (TLC) was performed in benzene:acetone:acetic acid (8:1:1) as a solvent, with the fluorescent silica gel plates HF_254_ (Merck, Darmstadt, Germany). ^1^H and ^13^C-NMR spectra were recorded on the Bruker Avance 600 MHz NMR Spectrometer (Bruker Biospin GmbH, Rheinstetten, Germany) at 293 K with DMSO-d^6^ was as a solvent and tetramethylsilane (TMS) as an internal standard. The mass spectra were recorded by the LC/MS/MS API 2000 (Applied Biosystems/MDS SCIEX, Redwood City, CA, USA). Absorbance for antioxidant activity was measured with Helious ϒ, UV-Vis spectrophotometer (Thermo spectronic, Waltham, MA, USA).

### 3.2. DES Preparation

DES was prepared as described by Amić and Molnar [14]. Briefly, choline chloride and urea (molar ratio 1:2) were stirred at 80 °C until the clear liquid was formed. DES was cooled to room temperature and used as such in further synthesis of the rhodanine compounds.

### 3.3. Knoevenagel Condensation in Choline Chloride:Urea (1:2) DES

An equimolar ratio of the corresponding rhodanine and aldehyde was mixed together in DES. The mixture was heated to 90 °C and stirred until completion of the reaction (monitored by TLC). Upon completion, water was added to the mixture and crude product was collected by filtration.

For investigation of DES recyclability, we performed experiments as follows: After filtration of desired product, water was evaporated under vacuum and reactants were added to DES thus obtained. After completion of reaction products were isolated again and the isolated yield calculated for this 2nd run. The same procedure was repeated for several times as stated in Table 1.

*(Z)-5-(2-methoxybenzylidene)-2-thioxothiazolidin-4-one (**2a**)*. Yield 47%; *R*_f_ = 0.74; Mp = 251 °C; ^1^H-NMR (300 MHz, ppm, DMSO-*d*_6_): 3.89 (s, 3H, OCH_3_), 7.07–7.10 (t, *J* = 7.34 Hz, 1H, arom.), 7.13–7.15 (d, *J* = 8.80 Hz, 1H, arom.), 7.36–7.38 (m, 1H, arom.), 7.48–7.50 (m, 1H, arom.), 7.78 (s, 1H, =CH), 13.76 (s, 1H, NH); ^13^C-NMR (DMSO-*d*_6_) δ (ppm): 196.01, 169.40, 158.09, 132.94, 129.61, 126.64, 125.26, 121.16, 111.92, 55.72; MS: *m*/*z*: 249.90 (M−) (251.32). (Supplementary material 2).

*(Z)-5-(4-(dimethylamino)benzylidene)-2-thioxothiazolidin-4-one (**2b**)*. Yield 78%; *R*_f_ = 0.58; Mp = 280 °C (lit. [35] 284–286 °C); ^1^H-NMR (300 MHz, ppm, DMSO-*d*_6_): 3.04 (s, 6H, 2CH_3_), 6.80-6.83 (d, *J* = 8.67 Hz, 2H, arom.), 7.40-7.43 (d, *J* = 9.04 Hz, 2H, arom), 7.51 (s, 1H, =CH), 13.55 (s, 1H, NH); ^13^C-NMR (DMSO-*d*_6_) δ (ppm): 195.49, 169.95, 152.22, 133.69, 133.35, 120.26, 117.87, 112.64, 40.00; MS: *m*/*z*: 263.10 (M−) (264.37). (Supplementary material 2).

*(Z)-5-(3-methoxybenzylidene)-2-thioxothiazolidin-4-one (**2c**)*. Yield 44%; *R*_f_ = 0.72; Mp = 246 °C (lit. [36] 247 °C); ^1^H-NMR (300 MHz, ppm, DMSO-*d_6_*): 3.81 (s, 3H, OCH_3_), 7.08–7.09 (dd, *J* = 8.07 Hz, *J* = 2.2 Hz, 1H, arom.), 7.15–7.16 (dd, *J* = 4.03 Hz, *J* = 1.8 Hz, 2H, arom), 7.46 (t, *J* = 8.07 Hz, 1H, arom.), 7.62 (s, 1H, =CH), 13.86 (s, 1H, NH); ^13^C-NMR (DMSO-*d*_6_) δ (ppm): 195.63, 169.31, 159.64, 134.27, 131.55, 130.53, 125.79, 122.33, 116.69, 115.55, 55.27; MS: *m*/*z*: 249.90 (M−) (251.32). (Supplementary material 2).

*(Z)-2-thioxo-5-(3,4,5-trimethoxybenzylidene)thiazolidin-4-one (**2d**)*. Yield 29%; *R*_f_ = 0.70; Mp = 190 °C; ^1^H-NMR (300 MHz, ppm, DMSO-*d*_6_): 3.74 (s, 3H, OCH_3_), 3.84 (s, 6H, 2OCH_3_), 6.90 (s, 2H, arom.), 7.60 (s, 1H, =CH), 13.82 (s. 1H, NH); ^13^C-NMR (DMSO-*d*_6_) δ (ppm): 195.54, 169.33, 153.22, 139.65, 131.98, 128.42, 124.31, 107.86, 60.19, 55.97; MS: *m*/*z*: 310.00 (M−) (311.38). (Supplementary material 2).

*(Z)-5-(3,4-dihydroxybenzylidene)-2-thioxothiazolidin-4-one (**2e**)*. Yield 36%; *R*_f_ = 0.42; Mp = 287–289 °C; ^1^H-NMR (300 MHz, ppm, DMSO-*d*_6_): 6.87 (s, 1H, arom.), 6.98–7.01 (m, 2H, arom), 7.47 (s, 1H, =CH), 9.53 (s, 1H, OH), 9.47 (s, 1H, OH), 13.67 (s, 1H, NH); ^13^C-NMR (DMSO-*d*_6_) δ (ppm): 196.09, 170.08, 149.67, 146.53, 133.22, 125.37, 124.83, 121.26, 117.10, 116.90; MS: *m/z*: 252.00 (M−) (253.30). (Supplementary material 2).

*(Z)-5-(4-hydroxy-3-methoxybenzylidene)-2-thioxothiazolidin-4-one (**2f**)*. Yield 53%; *R*_f_ = 0.67; Mp = 220 °C; ^1^H-NMR (300 MHz, ppm, DMSO-*d*_6_): 3.83 (s, 3H, OCH_3_), 6.93–6.94 (d, *J* = 8.1 Hz, 1H, arom.), 7.08 (m, 1H, arom.), 7.14 (s, 1H, arom.), 7.56 (s, 1H, =CH), 10.10 (s, 1H, OH), 13.66 (s, 1H, NH); ^13^C-NMR (DMSO-*d*_6_) δ (ppm): 195.46, 169.22, 149.95, 148.06, 132.73, 125.02, 124.33, 121.07, 116.30, 114.29, 55.59; MS: *m/z*: 266.00 (M−) (267.32). (Supplementary material 2).

*(Z)-5-(2-hydroxybenzylidene)-2-thioxothiazolidin-4-one (**2g**)*. Yield 32%; *R*_f_ = 0.79; Mp = 201 °C; ^1^H-NMR (300 MHz, ppm, DMSO-*d*_6_): 7.32–7.35 (m, 1H, arom.), 7.45–7.47 (d, *J* = 8.8 Hz, 2H, arom.), 7.58–7.61 (m, 1H, arom.), 7.72–7.75 (m, 1H, arom.), 8.15 (s, 1H, =CH); ^13^C-NMR (DMSO-*d*_6_) δ (ppm): 158.49, 151.87, 137.18, 131.58, 127.92, 124.88, 123.24, 119.18. 116.10 MS: *m/z*: 236.10 (M−) (237.30). (Supplementary material 2).

*(Z)-5-benzylidene-2-thioxothiazolidin-4-one (**2h**)*. Yield 26%; *R*_f_ = 0.67; Mp = 199 °C (lit. 202–203 °C [35,36,37,38]; ^1^H-NMR (300 MHz, ppm, DMSO-*d*_6_): 7.49–7.61 (m, 5H, arom.), 7.65 (s, 1H, =CH), 13.74 (s. 1H, NH); ^13^C-NMR (DMSO-*d*_6_) δ (ppm): 196.01, 170.19, 133.44, 132.06, 131.19, 130.93, 129.91; MS: *m/z*: 220.00 (M−) (221.30). (Supplementary material 2).

*(Z)-5-(pyridin-4-ylmethylene)-2-thioxothiazolidin-4-one (**2i**)*. Yield 30%; *R*_f_ = 0.96; Mp = 299 °C (294 °C [38]; ^1^H-NMR (300 MHz, ppm, DMSO-*d*_6_): 7.52–7.54 (m, 2H, pyr.), 7.58 (s, 1H, =CH), 8.72–8.74 (m, 2H, pyr.); MS: *m/z*: 220.90 (M−) (222.29). (Supplementary material 2).

*(Z)-5-(2,5-dimethoxybenzylidene)-2-thioxothiazolidin-4-one (**2j**)*. Yield 75%; *R*_f_ = 0.79; Mp = 240 °C; ^1^H-NMR (300 MHz, ppm, DMSO-*d*_6_): 3.76 (s, 3H, OCH_3_), 3.84 (s, 3H, OCH_3_), 6.87 (s, 1H, arom.), 7.08 (d, *J* = 1.5 Hz, 2H, arom.), 7.73 (s, 1H, =CH), 13.76 (s, 1H, NH); ^13^C-NMR (DMSO-*d*_6_) δ (ppm): 195.83, 169.34, 153.19, 152.55, 126.65, 125.70, 121.78, 118.50, 113.87, 113.18, 55.56; MS: *m/z*: 280.20 (M−) (281.35). (Supplementary material 2).

*(Z)-5-(4-hydroxybenzylidene)-2-thioxothiazolidin-4-one (**2k**)*. Yield 36%; *R*_f_ = 0.62; Mp = 285 °C; ^1^H-NMR (300 MHz, ppm, DMSO-*d*_6_): 6.91–6.93 (d, *J* = 8.8 Hz, 2H, arom.), 7.44–7.46 (d, *J* = 8.8 Hz, 2H, arom.), 7.55 (s, 1H, =CH), 10.4 (s, 1H, OH), 13.68 (s, 1H, NH); ^13^C-NMR (DMSO-*d*_6_) δ (ppm): 195.50, 169.48, 160.33, 132.28, 123.93, 120.96, 116.50, MS: *m/z*: 236.00 (M−) (237.30). (Supplementary material 2).

*(Z)-3-(4-chlorophenyl)-5-(4-hydroxy-3-methoxybenzylidene)-2-thioxothiazolidin-4-one (**2l**)*. Yield 72%; *R*_f_ = 0.58; Mp = 225–227 °C; ^1^H-NMR (300 MHz, ppm, DMSO-*d*_6_): 3.85 (s, 3H, OCH_3_), 6.96–6.99 (d, *J* = 8.1 Hz, 1H, arom.), 7.16–7.18 (d, *J* = 8.1 Hz, 1H, arom), 7.26 (s, 1H, arom.), 7.46–7.47 (d, *J* = 8.8 Hz, 2H, arom.), 7.63–7.64 (d, *J* = 8.1 Hz, 2H, arom.), 7.76 (s, 1H, =CH), 10.19 (s, 1H, OH); ); ^13^C-NMR (DMSO-*d*_6_) δ (ppm): 193.63, 166.85, 150.22, 148.13, 132.10, 130.78, 129.37, 125.12, 124.46, 118.65, 116.44, 114.65, 55.65 MS: *m/z*: 376.20 (M−) (377.85). (Supplementary material 2).

*(Z)-3-(4-chlorophenyl)-5-(4-(dimethylamino)benzylidene)-2-thioxothiazolidin-4-one (**2m**)*. Yield 48%; *R*_f_ = 0.77; Mp = 249–251 °C; ^1^H-NMR (300 MHz, ppm, DMSO-*d*_6_): 3.08 (s, 6H, 2CH_3_), 6.86–6.88 (m, 2H, arom.), 7.44–7.47 (m, 2H, arom.), 7.52–7.55 (m, 2H, arom), 7.62–7.65 (m, 2H, arom.), 7.73 (s, 1H, =CH); MS: *m/z*: 375.30 (M+) (374.90). (Supplementary material 2).

*(Z)-3-(4-chlorophenyl)-5-(4-hydroxybenzylidene)-2-thioxothiazolidin-4-one (**2n**)*. Yield 10%; *R*_f_ = 0.72; Mp = 292–294 °C; ^1^H-NMR (300 MHz, ppm, DMSO-*d*_6_): 6.96-6.97 (d, *J* = 8.8 Hz, 2H, arom.), 7.45–7.47 (m, 2H, arom), 7.55–7.56 (d, *J* = 8.8 Hz, 2H, arom.), 7.62–7.63 (m, 2H, arom.), 7.76 (s, 1H, =CH), 10.48 (s, 1H, OH); ^13^C-NMR (DMSO-*d*_6_) δ (ppm): 193.68, 166.86, 160.62, 134.16, 133.63, 133.27, 130.75, 129.25, 124.05, 118.50, 116.64; MS: *m/z*: 345.90 (M−) (346.85). (Supplementary material 2).

*(Z)-3-(4-chlorophenyl)-5-(2,5-dimethoxybenzylidene)-2-thioxothiazolidin-4-one (**2o**)*. Yield 32%; *R*_f_ = 0.78; Mp = 170–172 °C; ^1^H-NMR (300 MHz, ppm, DMSO-*d*_6_): 3.78 (s, 3H, OCH_3_), 3.87 (s, 3H, OCH_3_), 6.99 (s, 1H, arom.), 7.13 (d, *J* = 1.5 Hz, 2H, arom.), 7.43–7.46 (m, 2H, arom.), 7.60–7.63 (m, 2H, arom.), 7.90 (s, 1H, =CH); ^13^C-NMR (DMSO-*d*_6_) δ (ppm): 194.70, 167.18, 153.10, 134.57, 131.16, 129.85, 128.81, 122.49, 119.37, 114.95, 113.95, 56.69, 56.22; MS: *m/z*: 392.00 (M+) (391.89). (Supplementary material 2).

*(Z)-2-(5-((1,3-di-p-tolyl-1H-pyrazol-4-yl)methylene)-4-oxo-2-thioxothiazolidin-3-yl)acetic acid (**3a**)*. Yield 75%; *R*_f_ = 0.61; Mp = 240 °C (carb.); ^1^H-NMR (300 MHz, ppm, DMSO-*d*_6_): 2.38 (s, 3H, CH_3_), 2.41 (s, 3H, CH_3_), 4.47 (s, 2H, CH_2_), 7.36–7.40 (m, 4H, arom.), 7.53–7.54 (d, *J* = 2.3 Hz, 2H, arom.), 7.56 (s, 1H, =CH-.), 7.93–7.96 (d, *J* = 8.7 Hz, 2H, arom.), 8.78 (s, 1H, pyr); ); ^13^C-NMR (DMSO-*d*_6_) δ (ppm): 139.24, 137.59, 136.98, 130.61, 130.20, 129.17, 119.92, 119.72, 116.02, 21.1; MS: *m/z*: 448.20 (M−) (449.54). (Supplementary material 2).

*(Z)-2-(5-((3-(4-fluorophenyl)-1-(p-tolyl)-1H-pyrazol-4-yl)methylene)-4-oxo-2-thioxothiazolidin-3-yl)acetic acid (**3b**)*. Yield 72%; *R*_f_ = 0.66; Mp = >300 °C; ^1^H-NMR (300 MHz, ppm, DMSO-*d*_6_): 2.44 (s, 3H, CH_3_), 4.45 (s, 2H, CH_2_), 7.36–7.39 (m, 4H, arom.), 7.45 (s, 1H, =CH-), 7.69–7.71 (m, 2H, arom.), 7.93–7.96 (m, 2H, arom.), 8.78 (s, 1H, pyr); ); MS: *m/z*: 452.10 (M−) (453.51). (Supplementary material 2).

*(Z)-2-(5-((3-(4-methoxyphenyl)-1-(p-tolyl)-1H-pyrazol-4-yl)methylene)-4-oxo-2-thioxothiazolidin-3-yl)acetic acid (**3c**)*. Yield 66%; *R*_f_ = 0.70; Mp = 250 °C (carb.); ^1^H-NMR (300 MHz, ppm, DMSO-*d*_6_): 2.38 (s, 3H, CH_3_), 3.86 (s, 3H, OCH_3_), 4.59 (s, 2H, CH_2_), 7.11–7.14 (m, 2H, arom.), 7.35–7.37 (d, *J* = 8.3 Hz, 2H, arom.), 7.57 (s, 1H, =CH-), 7.60-7.64 (m, 2H, arom.), 7.90–7.93 (d, *J* = 8.7 Hz, 2H, arom.), 8.70 (s, 1H, pyr); ); ^13^C-NMR (DMSO-*d*_6_) δ (ppm): 193.14, 167.13, 160.82, 137.54, 130.59, 124.39, 119.87, 114.99, 55.81, 40.19, 20.96; MS: *m/z*: 464.20 (M−) (465.54). (Supplementary material 2).

*(Z)-2-(5-((3-(4-chlorophenyl)-1-(p-tolyl)-1H-pyrazol-4-yl)methylene)-4-oxo-2-thioxothiazolidin-3-yl)acetic acid (**3d**)*. Yield 63%; *R*_f_ = 0.84; Mp = 318 °C; ^1^H-NMR (300 MHz, ppm, DMSO-*d*_6_): 2.38 (s, 3H, CH_3_), 4.45 (s, 2H, CH_2_), 7.33–7.35 (m, 4H, arom.), 7.51 (s, 1H, =CH-.), 7.69–7.74 (d, *J* = 7.4 Hz, 2H, arom.), 7.93–7.96 (m, 2H, arom.), 8.78 (s, 1H, pyr); ); ^13^C-NMR (DMSO-*d*_6_) δ (ppm): 193.91, 167.51, 138.46, 131.66, 131.23, 130.31, 123.04, 120.69, 116.99, 49.65, 21.78; MS: *m/z*: 468.10 (M−) (469.97). (Supplementary material 2).

*(Z)-2-(5-((3-(4-bromophenyl)-1-(p-tolyl)-1H-pyrazol-4-yl)methylene)-4-oxo-2-thioxothiazolidin-3-yl)acetic acid (**3e**)*. Yield 78%; *R*_f_ = 0.87; Mp = 277 °C; ^1^H-NMR (300 MHz, ppm, DMSO-*d*_6_): 2.39 (s, 3H, CH_3_), 4.41 (s, 2H, CH_2_), 7.35-7.37 (d, *J* = 8.3 Hz, 2H, arom.), 7.53 (s, 1H, =CH-), 7.61–7.64 (d, *J* = 8.7 Hz, 2H, arom.), 7.76–7.79 (d, *J* = 8.3 Hz, 2H, arom.), 7.91–7.94 (d, *J* = 8.7 Hz, 2H, arom.), 8.74 (s, 1H, pyr); ); ^13^C-NMR (DMSO-*d*_6_) δ (ppm): 193.88, 167.38, 138.62, 137.80, 133.28, 132.04, 131.22, 130.19, 123.62, 120.54, 116.84, 49.65, 21.31; MS: *m/z*: 514.00 (M−) (514.42). (Supplementary material 2).

*(Z)-2-(5-((3-(4-iodophenyl)-1-(p-tolyl)-1H-pyrazol-4-yl)methylene)-4-oxo-2-thioxothiazolidin-3-yl)acetic acid (**3f**)*. Yield 67%; *R*_f_ = 0.89; Mp = 300 °C (carb.); ^1^H-NMR (300 MHz, ppm, DMSO-*d*_6_): 2.36 (s, 3H, CH_3_), 4.50 (s, 2H, CH_2_), 7.35–7.36 (d, *J* = 8.1 Hz, 2H, arom.), 7.44–7.46 (m, 2H, arom.), 7.51 (s, 1H, =CH−.), 7.92–7.96 (m, 4H, arom.), 8.78 (s, 1H, pyr); ); ^13^C-NMR (DMSO-*d*_6_) δ (ppm): 153.03, 137.73, 136.38, 130.76, 130.53, 129.86, 119.29, 115.47, 95.89, 20.33; MS: *m/z*: 560.10 (M−) (561.42). (Supplementary material 2).

### 3.4. Antioxidant Activity

Antioxidant activity was performed according to our previous work [39]. All compounds and DPPH radical were prepared in dimethyl sulfoxide (DMSO) as a solvent. Briefly, 750 µL of 0.2 mM solution of each compound was mixed with 750 µL of 0.2 mM DPPH radical solution, incubated for 30 minutes and absorbance of each mixture was measured at 517 nm. DPPH scavenging activity was calculated according to Equation (2).

(2) $$\%\;DPPH\;scavenging=\frac{A_{b}+A_{s}-A_{m}}{A_{b}}\times 100,$$

*A*_b_—absorbance of 0.1 mM DMSO solution of DPPH radical at 517 nm.

*A*_s_—absorbance of 0.1 mM DMSO solution of test compound at 517 nm.

*A*_m_—absorbance of DMSO mixture of test compound and DPPH radical at 517 nm.

### 3.5. QSAR Method

#### 3.5.1. Data Set

The dataset used for building QSAR models consists of 21 molecules whose antioxidant activities were measured and described in the present study. Antioxidant activity, expressed as % DPPH, were converted in the form of the negative logarithm of % DPPH/100 (pDPPH) and presented in Table 2.

#### 3.5.2. Descriptor Calculation

The 3D structures of 21 molecules were optimized applying the Avogadro 1.2.0. (University of Pittsburgh, Pittsburgh, PA, USA) using the molecular mechanics force field (MM+) [40]. Subsequently, all structures were submitted to geometry optimization using the semiempirical AM1 method [39]. Two sets of descriptors were generated using Parameter Client (Virtual Computational Chemistry Laboratory, an electronic remote version of the Dragon program [41] and by ADMEWORKS ModelBuilder [42]. In order to reduce the huge number of calculated descriptors, firstly, zero value descriptors were excluded from the initial pool. Further exclusion was performed using QSARINS-Chem 2.2.1 (University of Insubria, Varese, Italy) [43]: Constant and semi-constant descriptors, i.e., those having chemical compounds with a constant value for more than 80%, and descriptors that are too inter-correlated (>85%) were rejected.

#### 3.5.3. Regression Analysis and Validation of Models

The best QSAR model was obtained by using a Genetic Algorithm (GA) using QSARINS. The number of descriptors (I) in the multiple regression equation was limited on two. The models have been assessed by: Fitting criteria; internal cross-validation using leave-one out (LOO) method. Robustness of QSAR models was tested by Y-randomization test. Considering a limited number of compounds, external validation was not possible. Fitting criteria included: The coefficient of determination (*R*^2^), adjusted *R*^2^ (*R*^2^_adj_), cross-validate *R*^2^ using leave-one-out method (*Q*^2^_loo_), global correlation among descriptors (*Kxx*), difference between global correlation between molecular descriptors and y the response variable, and global correlation among descriptors (Δ*K*), standard deviation of regression (*s*), and Fisher ratio (*F*). Internal validations also included the following parameters: Root-mean-square error of the training set (*RMSE*_tr_); root-mean-square error of the training set determined through cross validated LOO method (*RMSE*_cv_), concordance correlation coefficient of the training set (*CCC*_tr_), test set using LOO cross validation (CCC_cv_), mean absolute error of the training set (*MAE*_tr_), mean absolute error of the internal validation set (*MAE*_cv_), predictive residual sum of squares determined through cross-validated LOO method (*PRESS*_cv_) in the training set.

Investigation of the applicability domain of a prediction model was performed by leverage plot (plotting residuals vs. leverage of training compounds). The warning leverage h* is defined as 3*p*′/*n*, where *n* is the number of training compounds and *p*′ is the number of model adjustable parameters [44].

Investigation of the applicability domain of obtained model was performed by leverage plot (Williams plots) using QSARINS. External validation of model was left out due to a relatively small number of compounds. Robustness of QSAR model was tested by Y-randomization test.

### 3.6. Molecular Docking

The molecular docking of the most active compound **2e** was performed with *i*GEMDOCK (BioXGEM, Taiwan). Crystal coordinates of the haematopoietic cell tyrosine kinase (PDB ID: 2HCK) in the complex with quercetin was downloaded from Protein Dana Bank (https://www.rcsb.org/structure/2HCK). In the first step, structure of 2HCK was prepared, including the removal of water molecules and optimized protein structure using PyMOL (The PyMOL Molecular Graphics System, Version 2.0 Schrödinger, LLC, New York, NY, USA). Binding site of protein was defined according the bounded ligand. After preparing the protein target and optimized structure of the compound **2e** as a ligand, genetic parameters were set (population size 200; generations: 70; number of solution or poses: 2). The compound was docked into the binding site and protein-compound interaction profiles of electrostatic (Elec), hydrogen-bonding (Hbond), and van der Waals (vdW) interactions were generated. Energy-based scoring function or total energy (*E*) is: *E* = vdW + Hbond + Elec [45]. 

## 4. Conclusions

A series of rhodanine derivatives were synthesized in the Knoevenagel condensation of rhodanine and different aldehydes in deep eutectic solvent. Among all the synthesized compounds, benzylidene derivative of 5-aryldenerhodanines with in 3,4-hydroxyl moiety on phenyl ring exhibited the best DPPH radical scavenging activity. QSAR analysis revealed the importance of the following characteristics for antioxidant activity: Sphericity of molecule, ratio of symmetric atoms enhanced atomic mass along the principle axes regards to total number of atoms in molecule, and 3D distribution of the atoms higher electronegativity (O, N, and S) in molecules. Presences of hydroxyl groups and atoms of higher electronegativity are important for formation of interaction with tyrosine kinase. Molecular docking study confirmed that the most active compound binds to the tyrosine kinase Hck, at the same binding site as quercetin, mostly throughout the hydrogen bonds.

## Figures and Tables

**Table 1:** Influence of DES recycle on the yield of the final product.

Solvent	Yield (%)
choline chloride:urea	78
1st recycle	78
2nd recycle	74
3rd recycle	76

**Table 2:** Structure and the observed antioxidant activity of rhodanine derivatives expressed as % 1,1-diphenyl-2-picrylhydrazyl radical (DPPH) scavenging and negative value of % DPPH/100 (pDPPH).

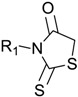														
**Cmp.**			**R_1_**					**%DPPH**				**pDPPH**		
**1**			H					3.6				1.444		
**2**			*p*-Cl-phenyl					94.4				0.025		
**3**			CH_2_COOH					23.9				0.622		
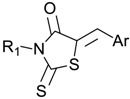														
**Cmp.**	**Ar**	**R_1_**		**% DPPH**	**pDPPH**		**Cmp.**		**Ar**		**R_1_**		**% DPPH**	**pDPPH**
**2a**	2-OCH_3_-phenyl	H		9.2	1.036		**2i**		4-pyridyl		H		21.5	0.668
**2b**	4-N(CH_3_)_2_-phenyl	H		1.1	1.959		**2j**		2,5-(OCH_3_)_2_-phenyl		H		13.9	0.857
**2c**	3-OCH_3_-phenyl	H		15.7	0.804		**2k**		4-OH-phenyl		H		5.8	1.237
**2d**	3,4,5-(OCH_3_)_3_-phenyl	H		10.1	0.996		**2l**		3-OH-4-OCH_3_-phenyl		4-Cl-phenyl		30.8	0.511
**2e**	3,4-(OH)_2_-phenyl	H		71.2	0.148		**2m**		4-N(CH_3_)_2_-phenyl		4-Cl-phenyl		20.6	0.686
**2f**	3-OCH_3_-4-OH-phenyl	H		39.2	0.407		**2n**		4-OH-phenyl		4-Cl-phenyl		3.4	1.469
**2g**	2-OH-phenyl	H		5.0	1.301		**2o**		2,5-(OCH_3_)_2_-phenyl		4-Cl-phenyl		0.1	3
**2h**	phenyl	H		14.2	0.848									
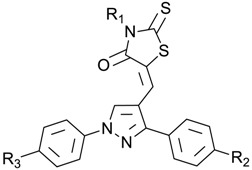														
**Cmp.**	**R_1_**	**R_2_**		**R_3_**		**% DPPH**				**pDPPH**				
**3a**	CH_2_COOH	-CH_3_		-CH_3_		0.5				2.301				
**3b**	CH_2_COOH	-F		-CH_3_		0.4				2.398				
**3c**	CH_2_COOH	-OCH_3_		-CH_3_		0.4				2.398				
**3d**	CH_2_COOH	-Cl		-CH_3_		0				0				
**3e**	CH_2_COOH	-Br		-CH_3_		2.4				1.62				
**3f**	CH_2_COOH	-I		-CH_3_		2.2				1.658				

**Table 3:** Docking score and energy of the interactions between protein residue and ligand **2e**.

Complex	Total Energy/kcal·mol^−1^	van der Waals Interaction	H Bond	Elec
2HCK + 2e	−77.1535	−53.5621	−23.5914	0
	**H Bond**	**Energy**	**van der Waals interaction**	**Energy**
	H-M-MET-341	−8.5	V-S-LEU-273	−8.07
	H-M-SER-345	−3.5	V-S-VAL-281	−4.13
	H-S-SER-345	−3.46	V-S-PHE-340	−4.79
	H-S-ASP-348	−6.60	V-M-GLY-344	−5.96
			V-M-SER-345	−5.28
			V-S-LEU-393	−4.10

## Supplementary Materials

Supplementary materials are available on line.
