# Supplementary material for: Environmentally Friendly Approach to Knoevenagel Condensation of Rhodanine in Choline Chloride: Urea Deep Eutectic Solvent and QSAR Studies on Their Antioxidant Activity
Source: Molecules. 2018 Jul 29;23(8):1897. doi: 10.3390/molecules23081897 (PMC6222480; doi:10.3390/molecules23081897)
Supplement: Supplementary file 1 [file molecules-23-01897-s001.zip › molecules-327901/molecules-327901.pdf]

Supplementary data

Environmentally friendly approach to Knoevenagel condensation of rhodanine in choline chloride:urea deep eutectic solvent and QSAR studies on their antioxidant activity

Maja Molnar[1*], Harshad Brahmbhatt[1], Vesna Rastija[2], Valentina Pavić[3], Mario Komar[1], Maja Karnaš[2] and Jurislav Babić[1]

(*Z*)-5-(2-methoxybenzylidene)-2-thioxothiazolidin-4-one (2a)

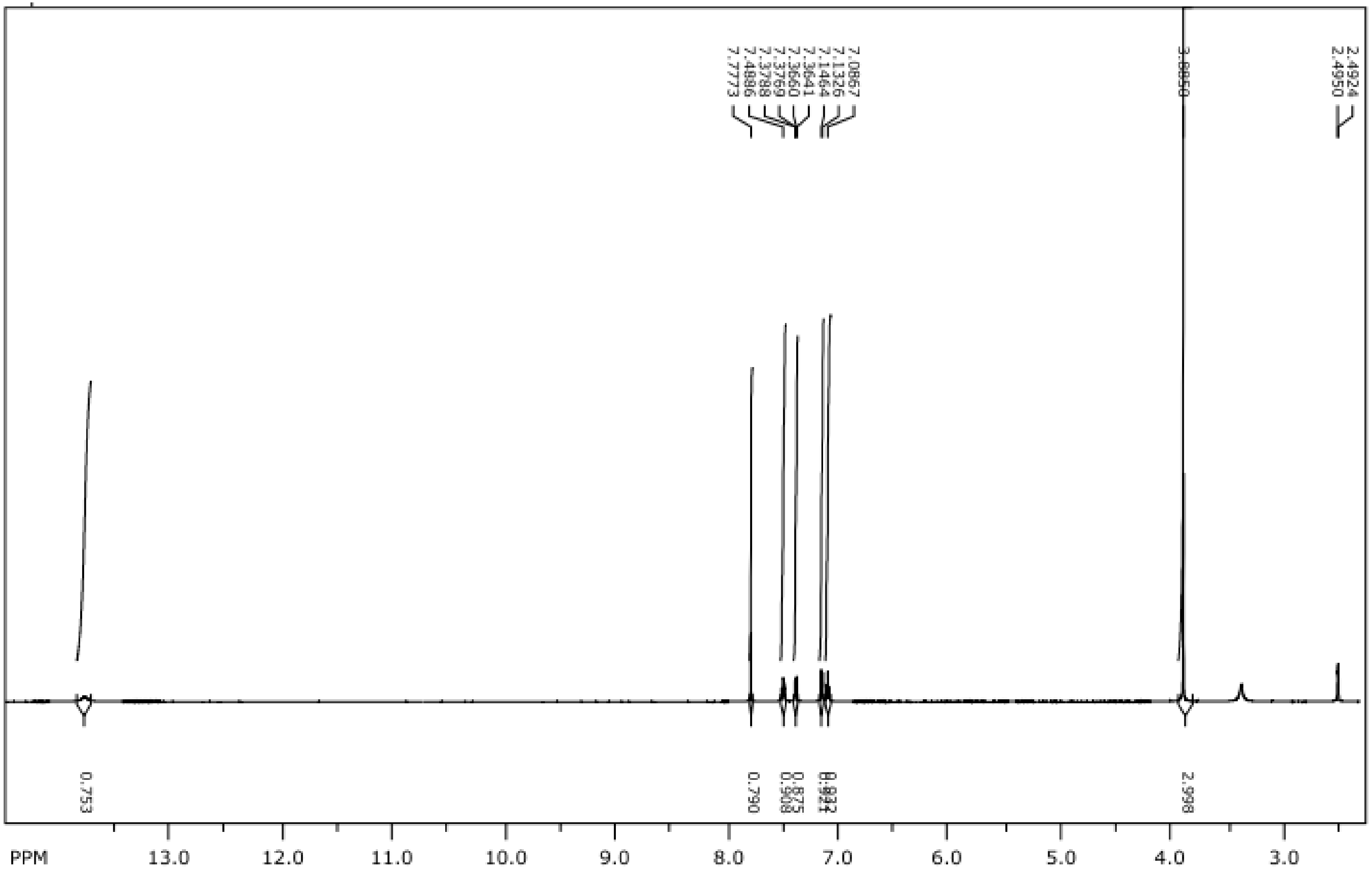

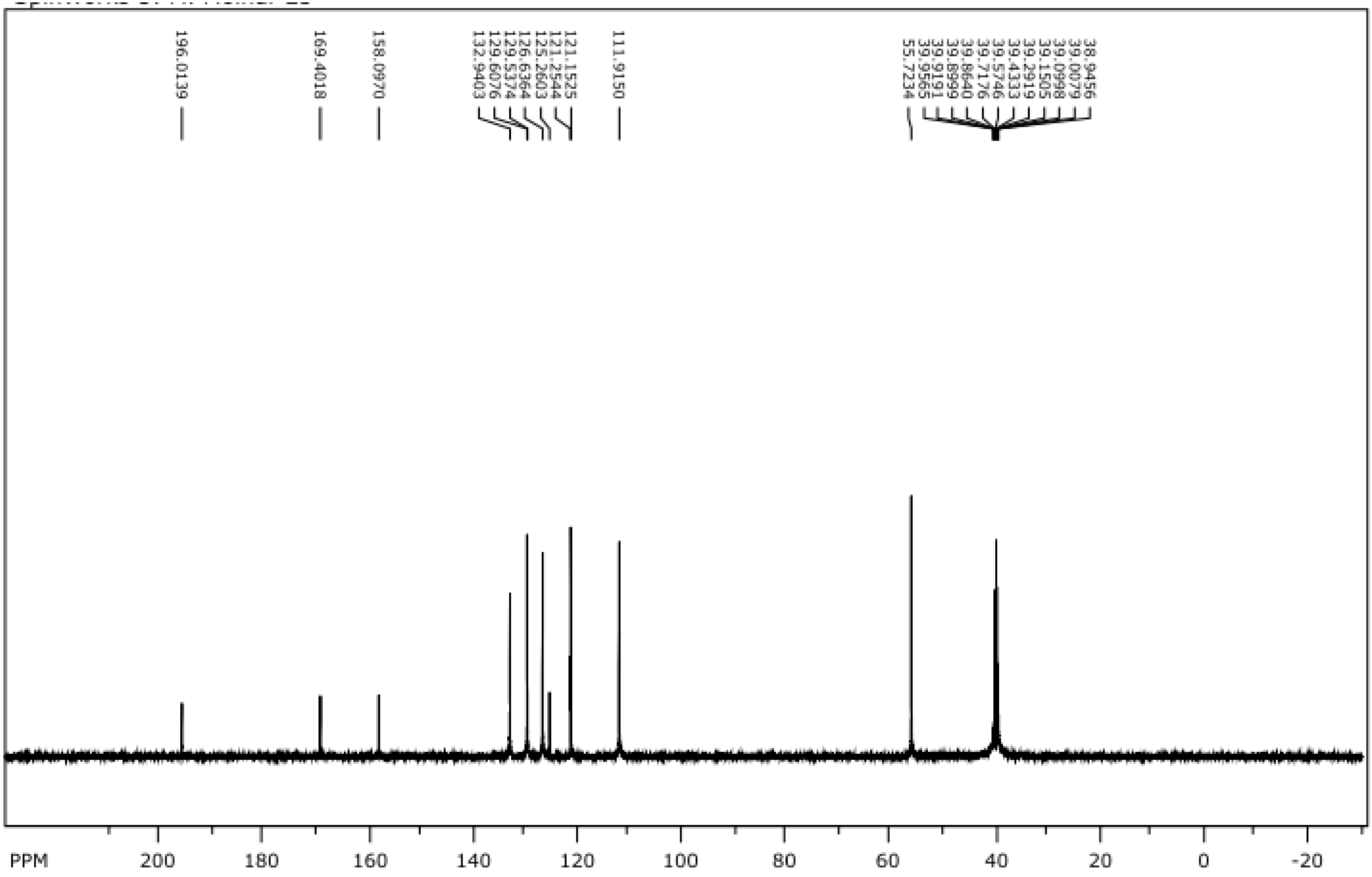
196.0139
169.4018
158.0970
132.9403
129.6076
129.5374
126.6364
125.2603
121.2544
121.1525
111.9150
55.7234
39.9565
39.9191
39.8999
39.8640
39.7176
39.5746
39.4333
39.2919
39.1505
39.0998
39.0079
38.9456
PPM
200
180
160
140
120
100
80
60
40
20
0
-20

-Q1: 60 MCA scans from Sample 1 (030_2b) of 030_2b.wiff (Turbo Spray) Max. 1.3e7 cps.

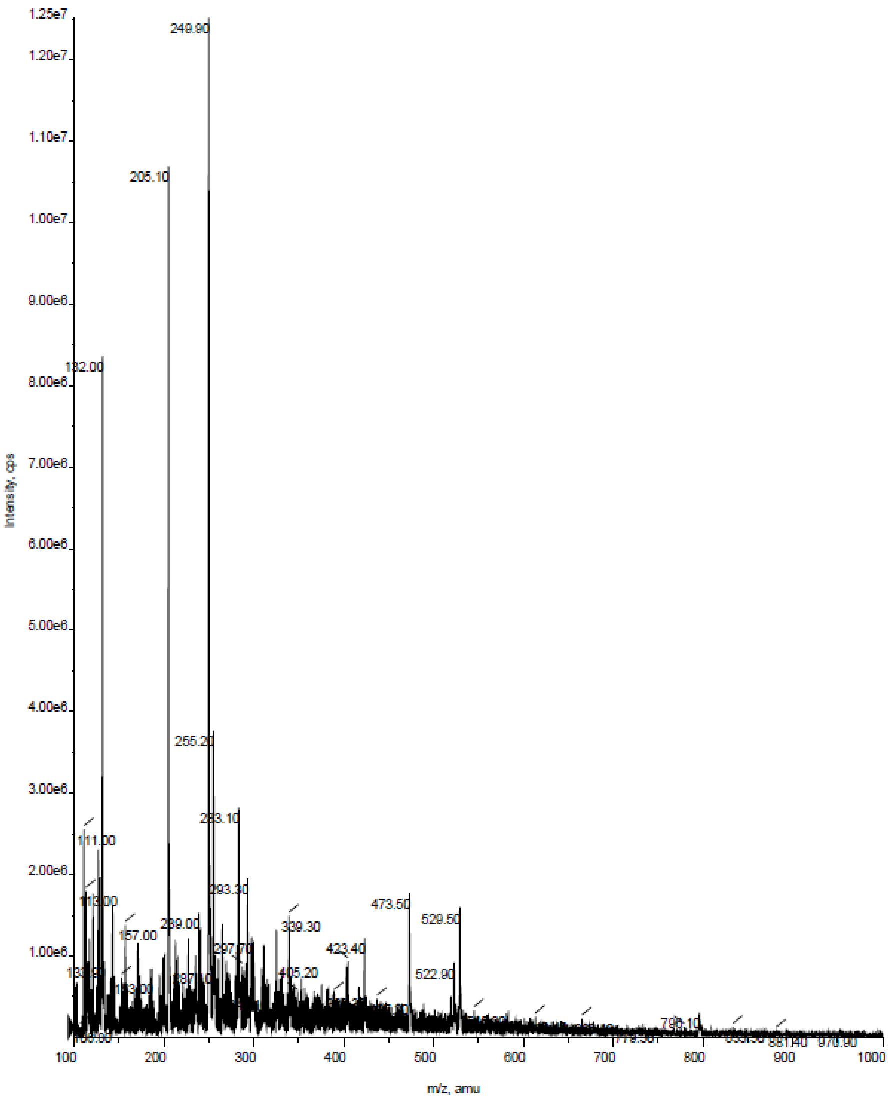

(*Z*)-5-(4-(dimethylamino)benzylidene)-2-thioxothiazolidin-4-one (2b)

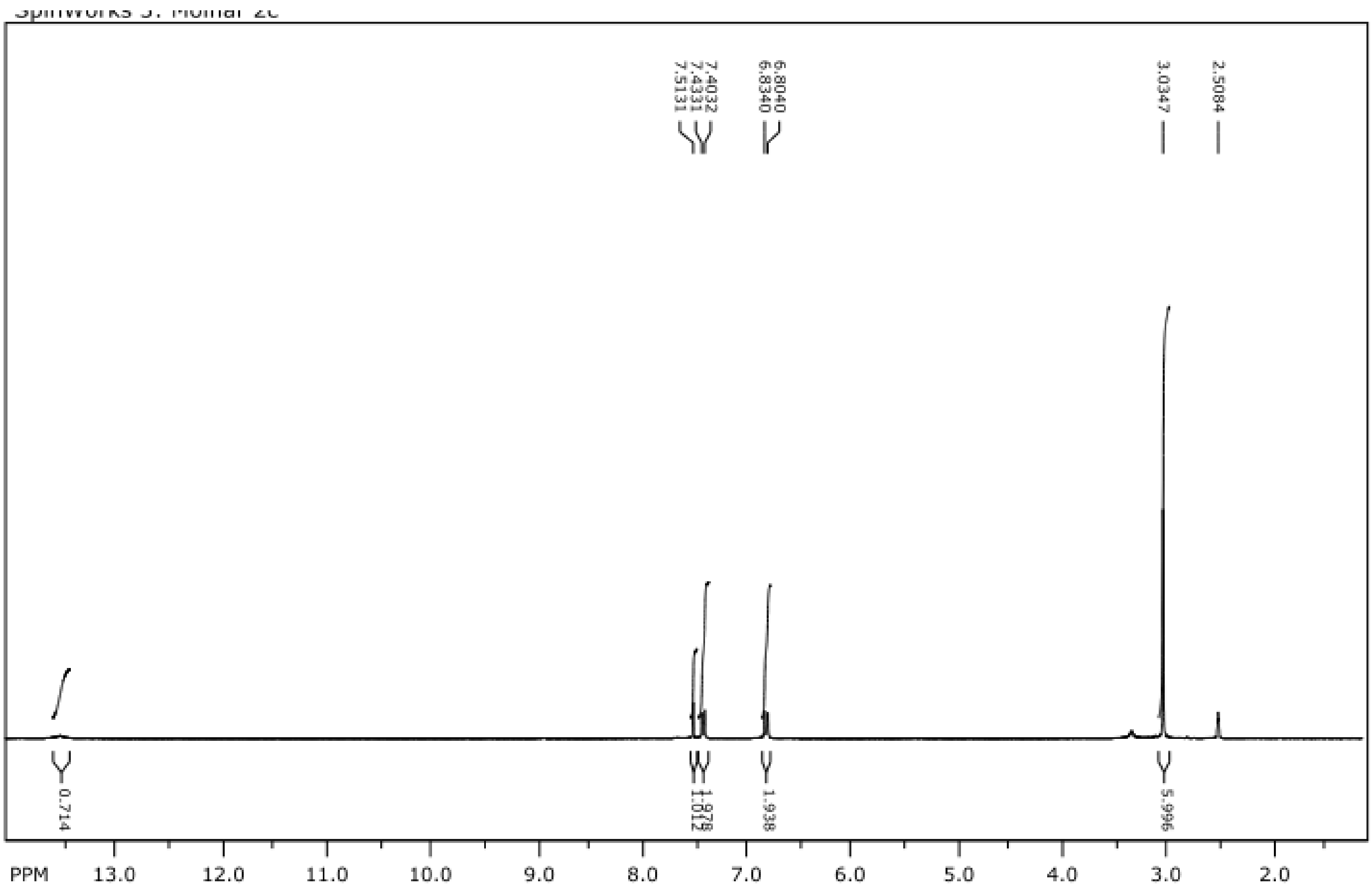

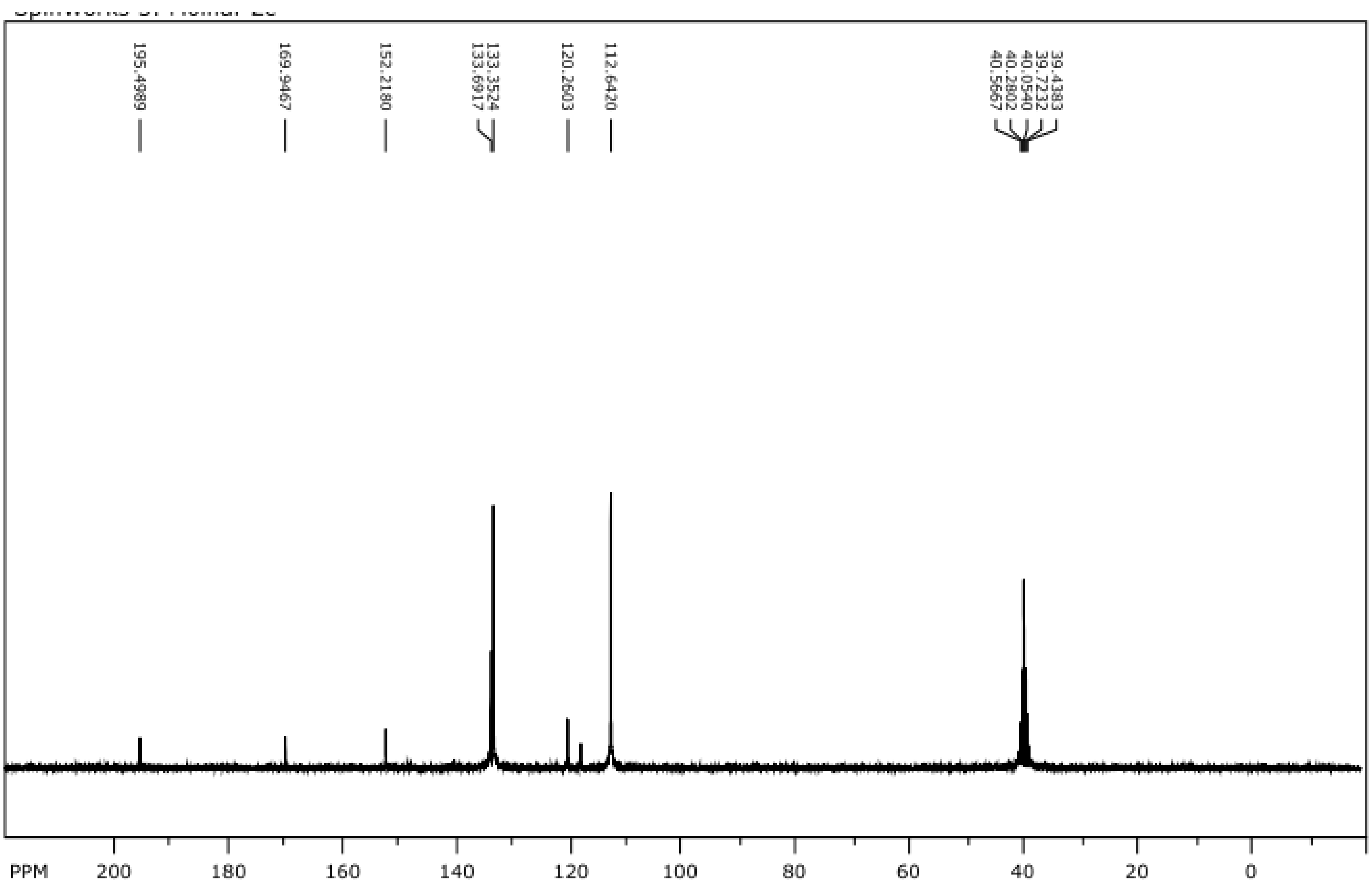
195.4989
169.9467
152.2180
133.3524
133.6917
120.2603
112.6420
39.4383
39.7232
40.0540
40.2802
40.5667
PPM
200
180
160
140
120
100
80
60
40
20
0

-Q1: 60 MCA scans from Sample 1 (032_2c) of 032_2c.wiff (Turbo Spray) Max. 1.6e7 cps.

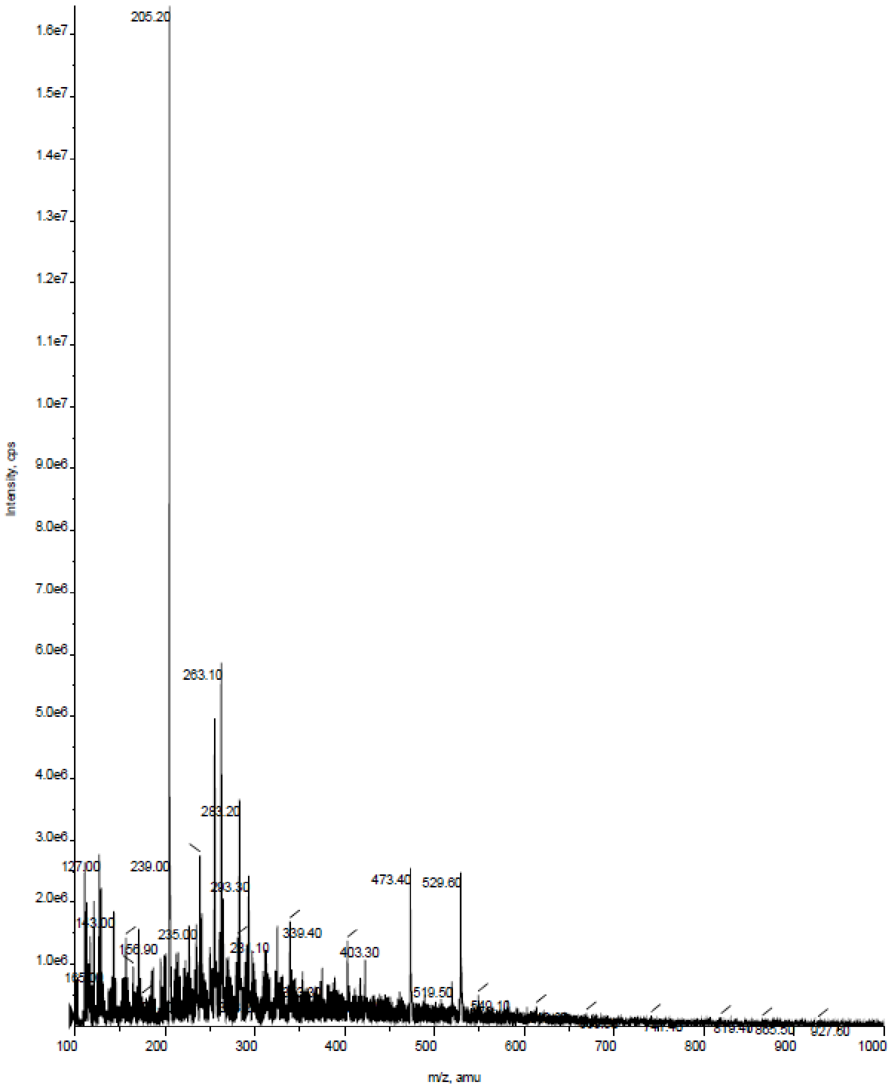

(*Z*)-5-(3-methoxybenzylidene)-2-thioxothiazolidin-4-one (2c)

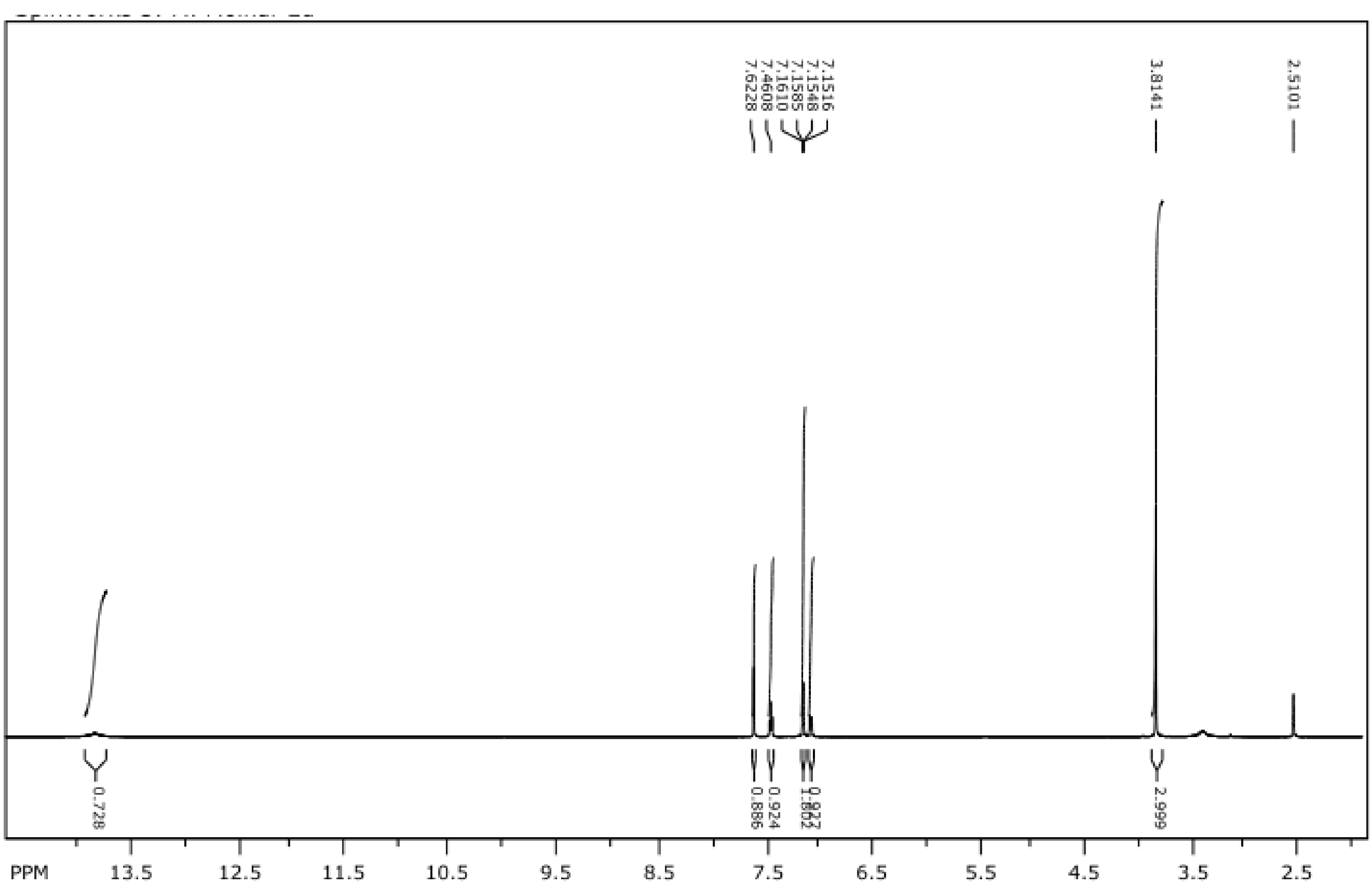

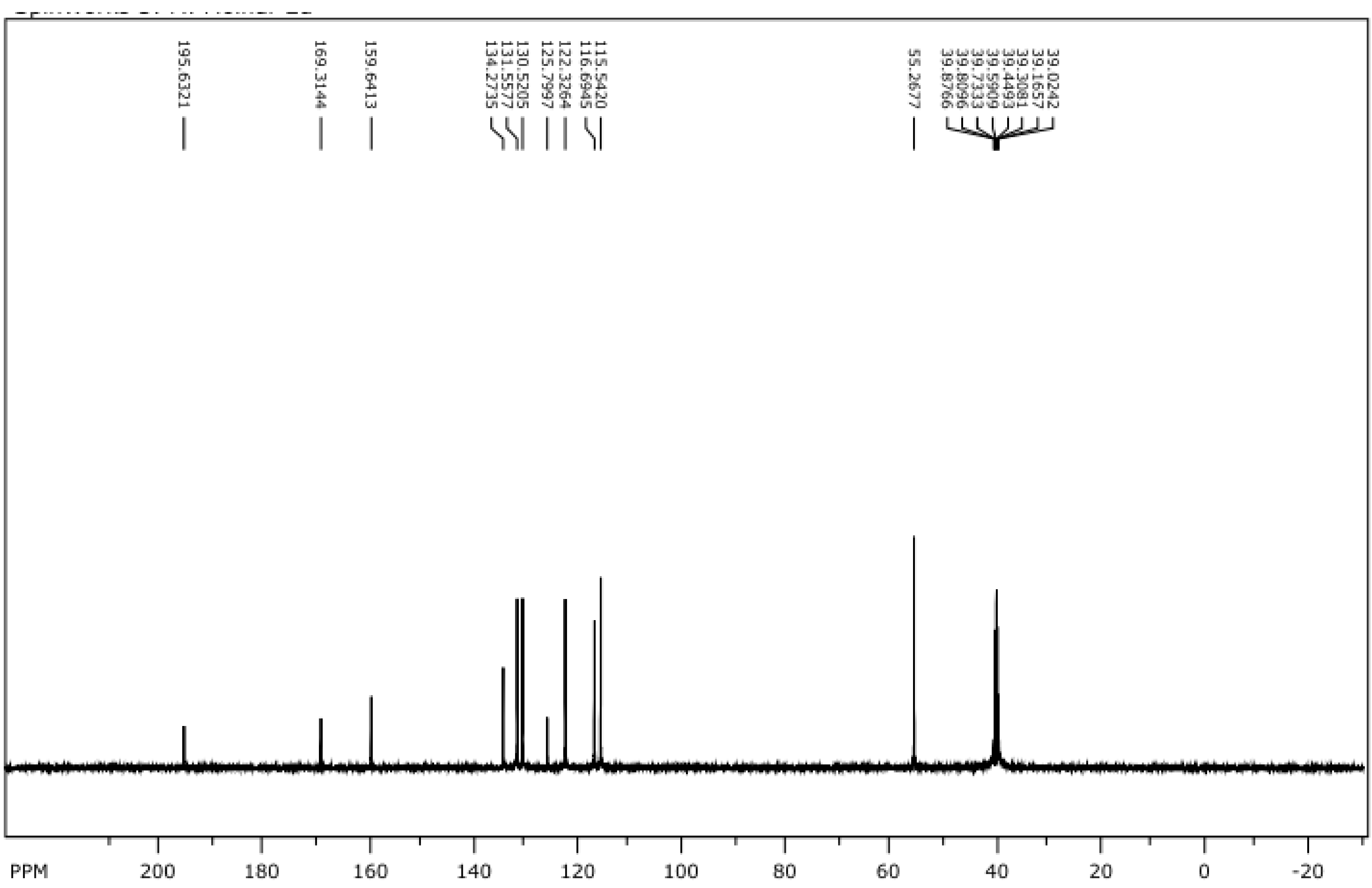
195.6321
169.3144
159.6413
134.2735
131.5577
130.5205
125.7997
122.3264
116.6945
115.5420
55.2677
39.8766
39.8096
39.7333
39.5909
39.4493
39.3081
39.1657
39.0242
PPM
200
180
160
140
120
100
80
60
40
20
0
-20

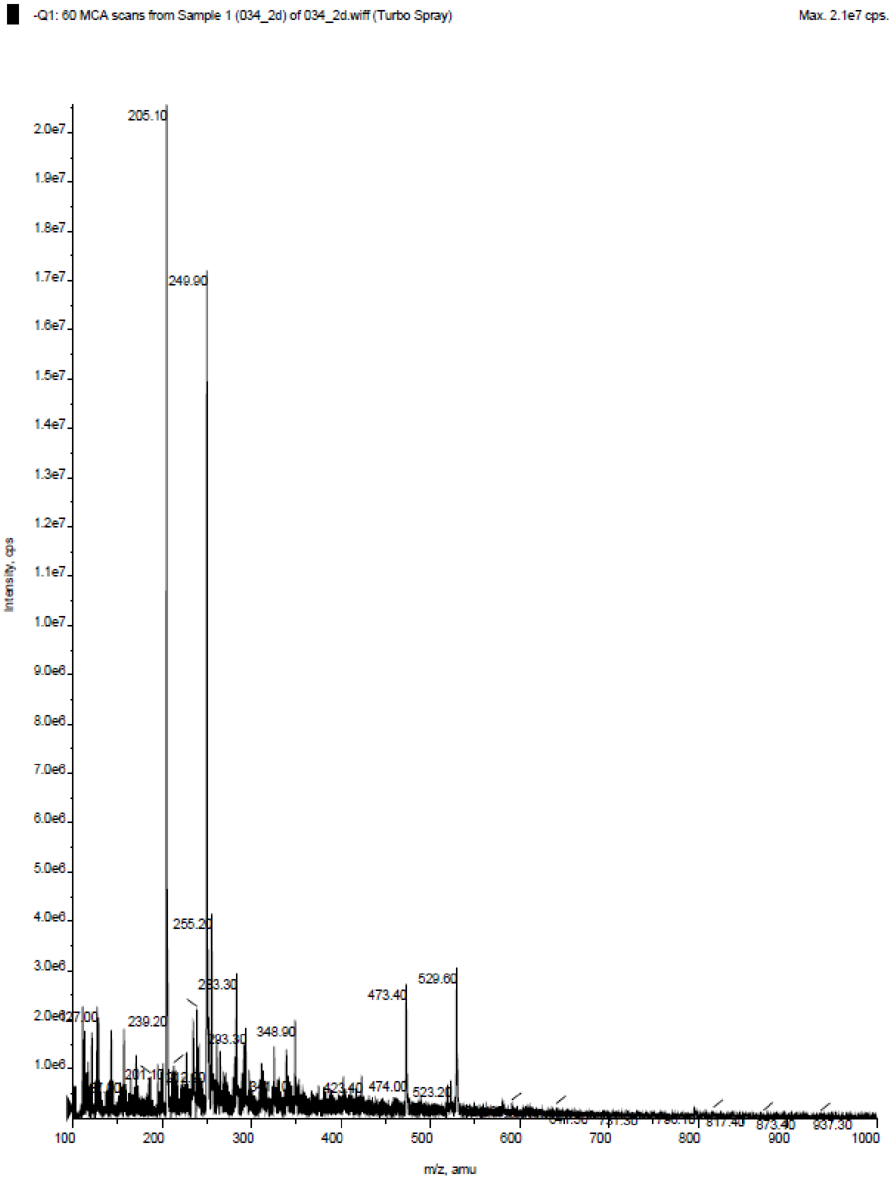
-Q1: 60 MCA scans from Sample 1 (034_2d) of 034_2d.wiff (Turbo Spray)
Max. 2.1e7 cps.
Intensity, cps
m/z, amu
205.10
249.90
255.20
473.40
529.60
239.20
348.90
293.30
423.40
474.00
523.20
817.40
873.40
937.30

(Z)-2-thioxo-5-(3,4,5-trimethoxybenzylidene)thiazolidin-4-one (2d)

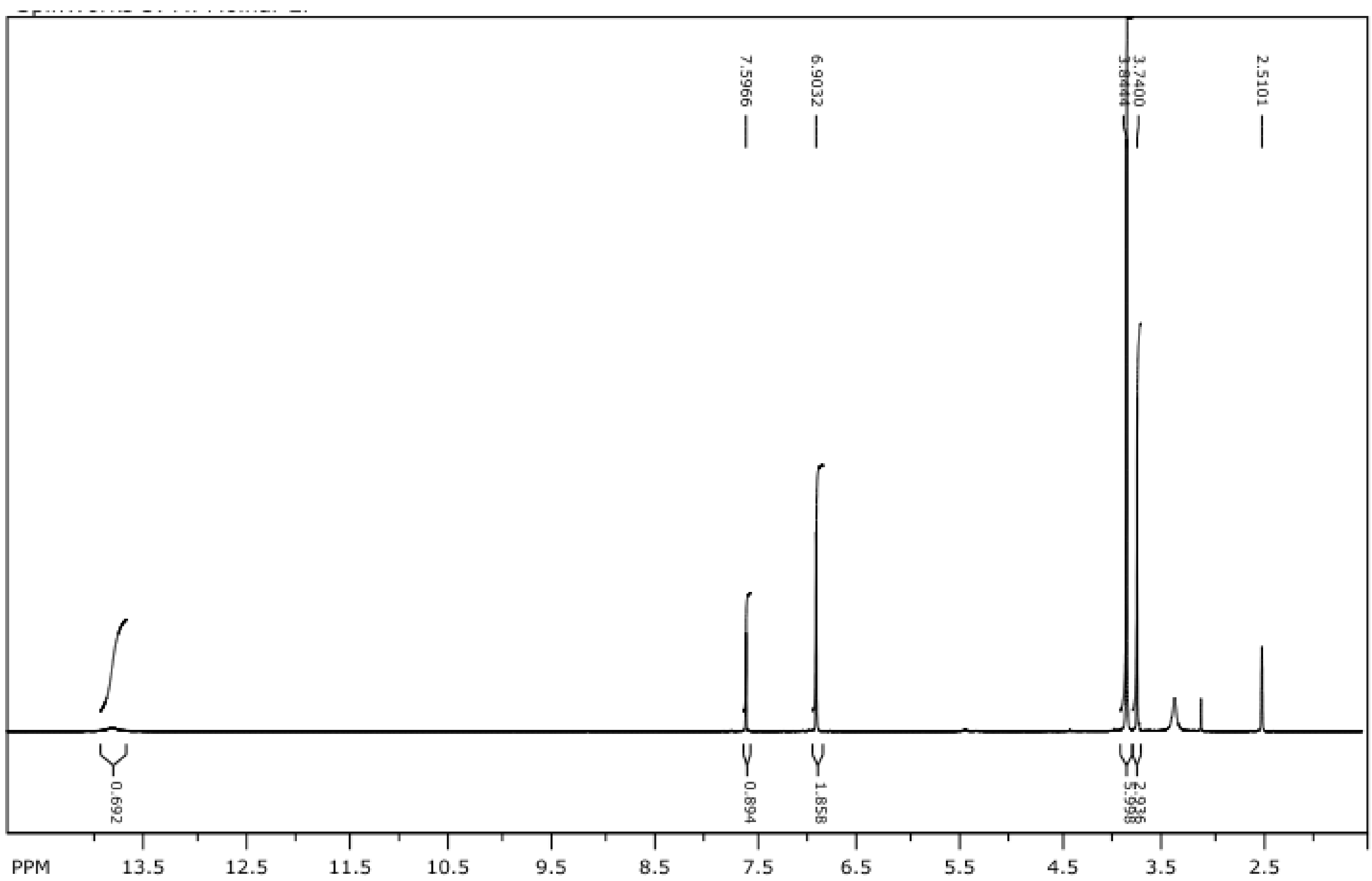

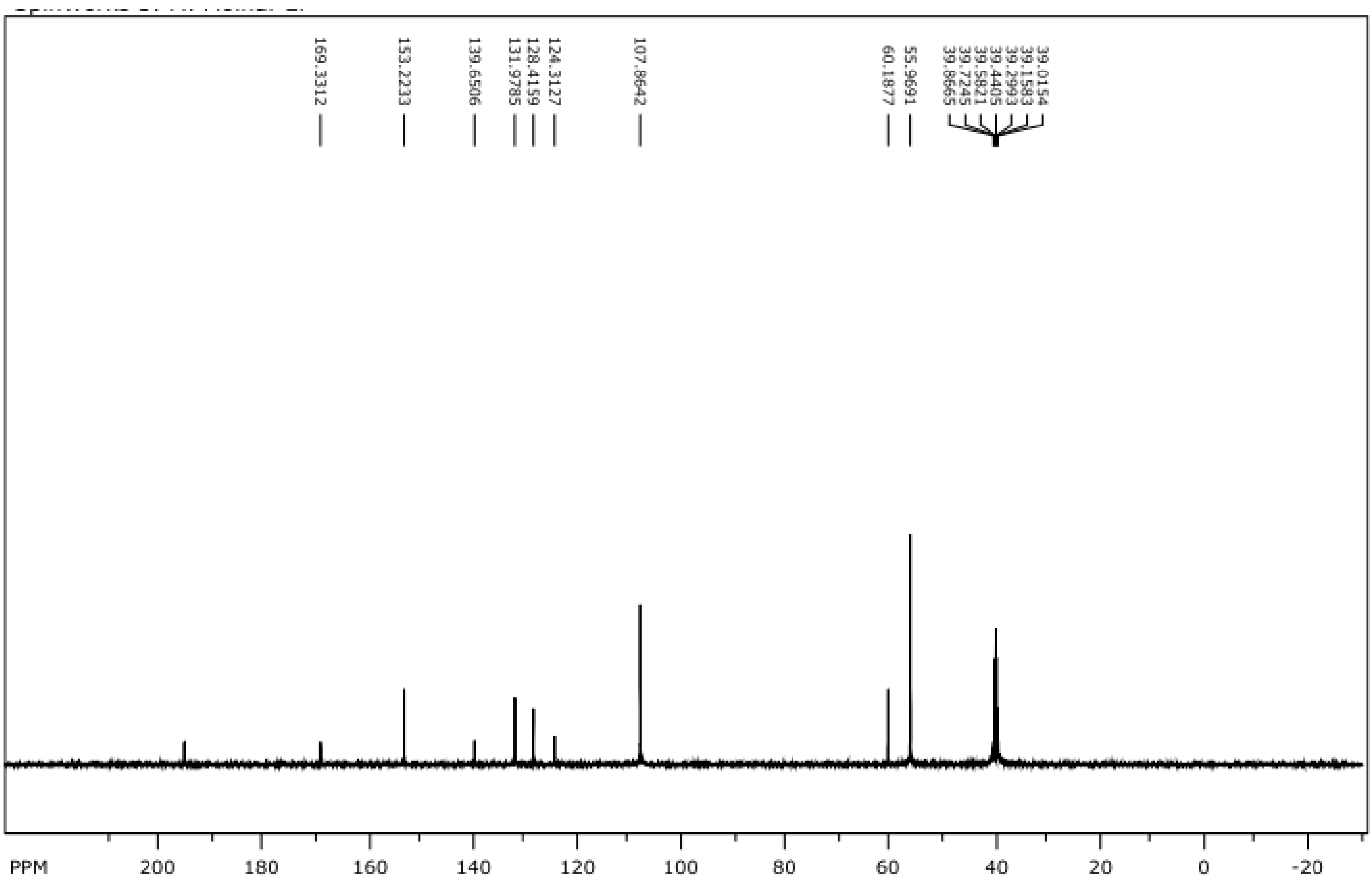
169.3312
153.2233
139.6506
131.9785
128.4159
124.3127
107.8642
60.1877
55.9691
39.8665
39.7245
39.5821
39.4405
39.2993
39.1583
39.0154
PPM
200
180
160
140
120
100
80
60
40
20
0
-20

-Q1: 60 MCA scans from Sample 1 (036_2f) of 036_2f.wiff (Turbo Spray)

Max. 2.7e7 cps.

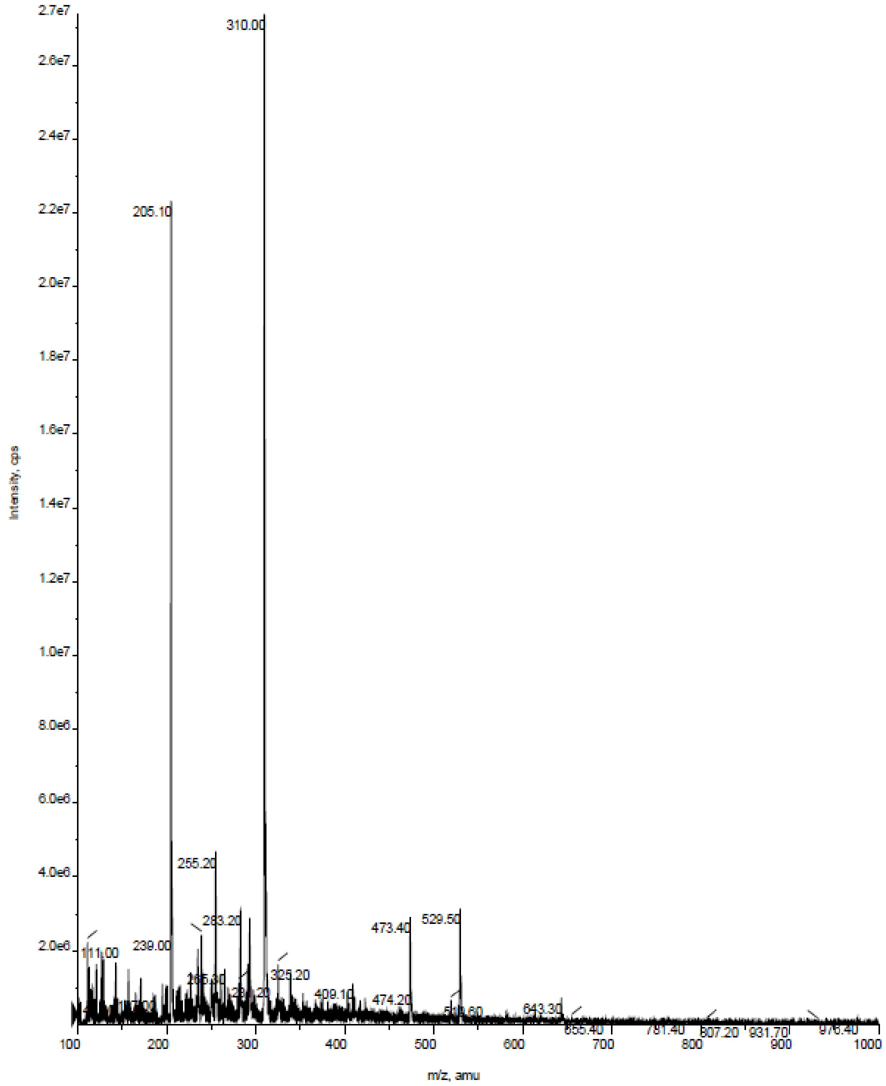

(*Z*)-5-(3,4-dihydroxybenzylidene)-2-thioxothiazolidin-4-one (2e)

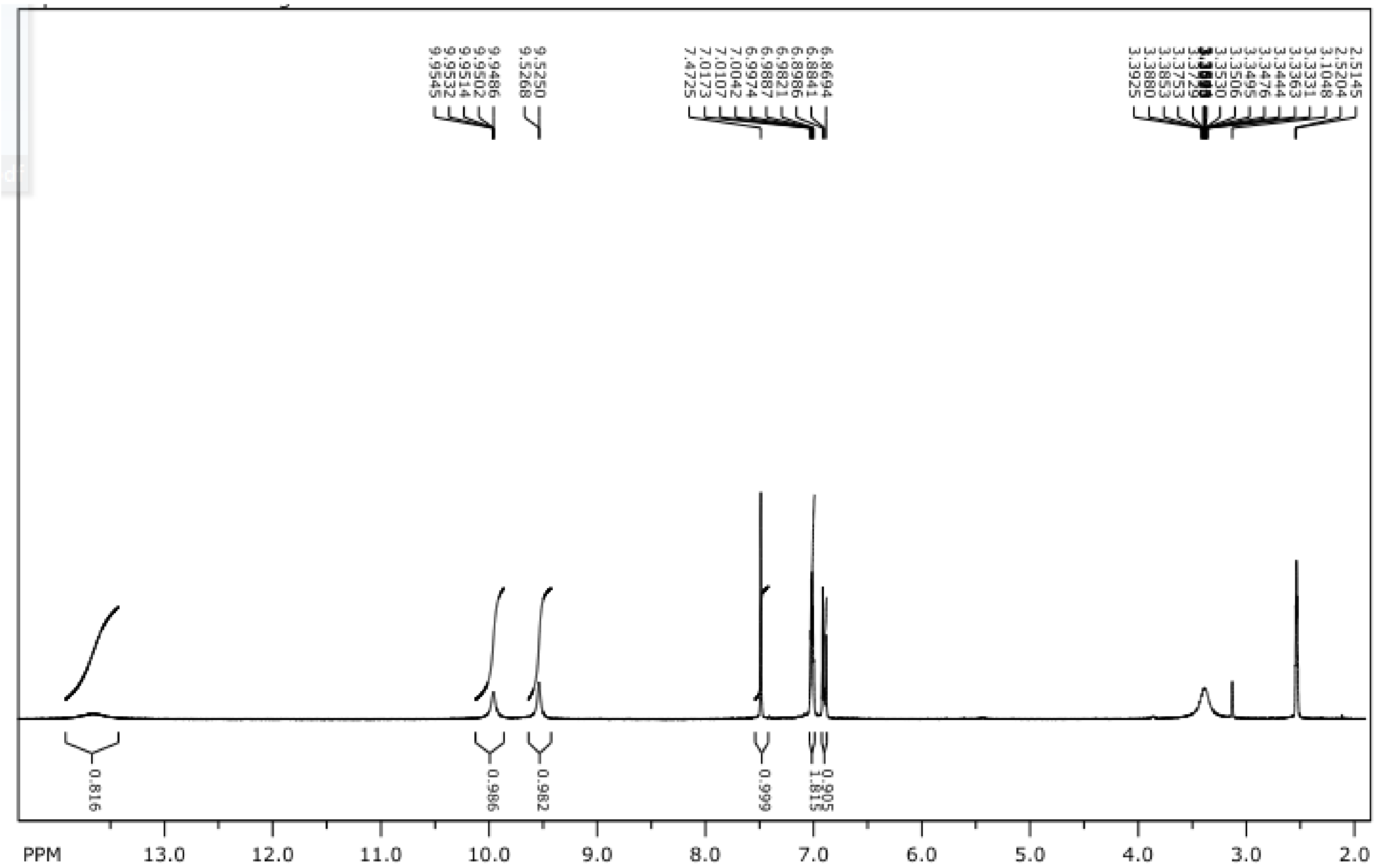

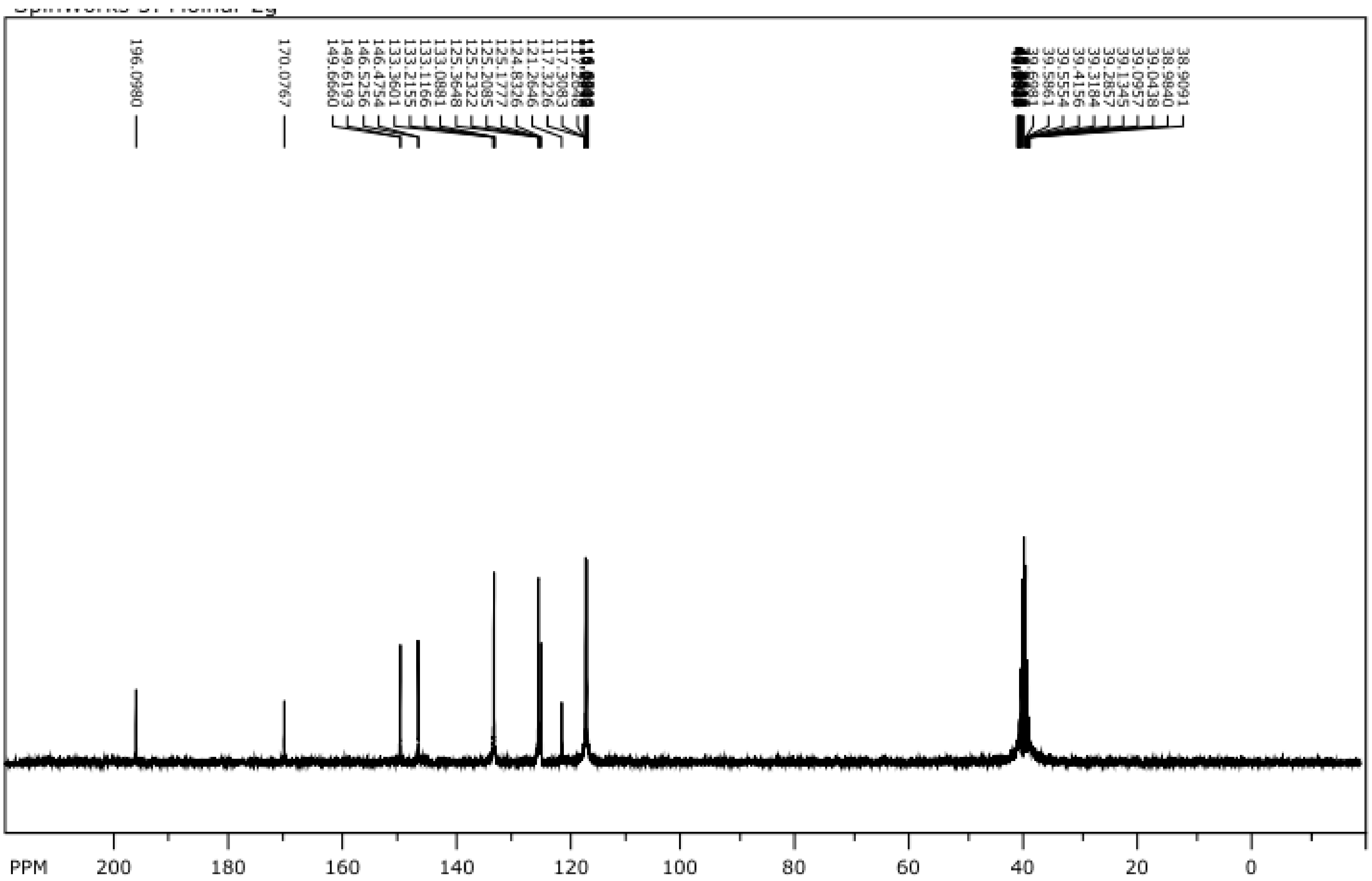
196.0980
170.0767
149.6660
149.6193
146.5256
146.4754
133.3601
133.2155
133.1166
133.0881
125.3648
125.2322
125.2085
125.1777
124.8326
121.2646
117.3226
117.3083
117.2048
39.5861
39.5554
39.4156
39.3184
39.2857
39.1345
39.0957
39.0438
38.9840
38.9091
PPM
200
180
160
140
120
100
80
60
40
20
0

-Q1: 60 MCA scans from Sample 1 (038_2g) of 038_2g.wiff (Turbo Spray) Max. 1.3e8 cps.

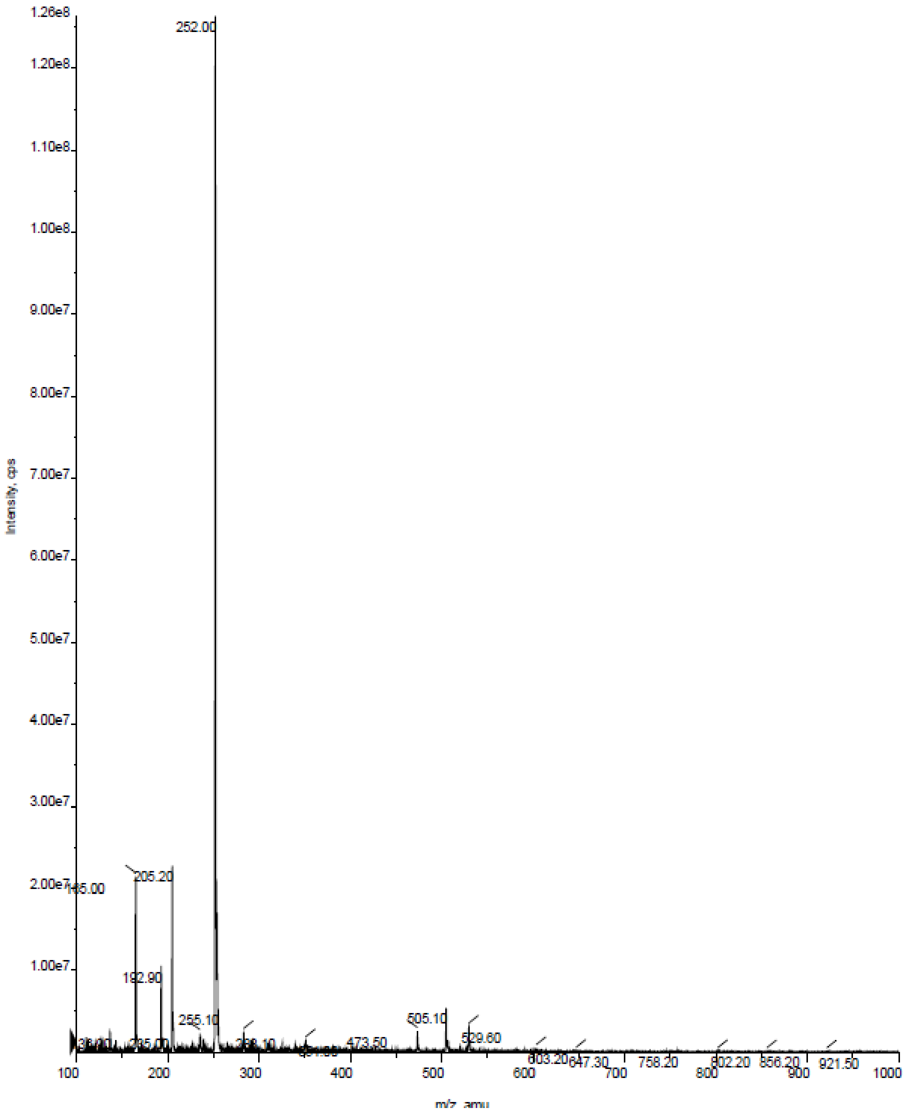

(*Z*)-5-(4-hydroxy-3-methoxybenzylidene)-2-thioxothiazolidin-4-one (2f)

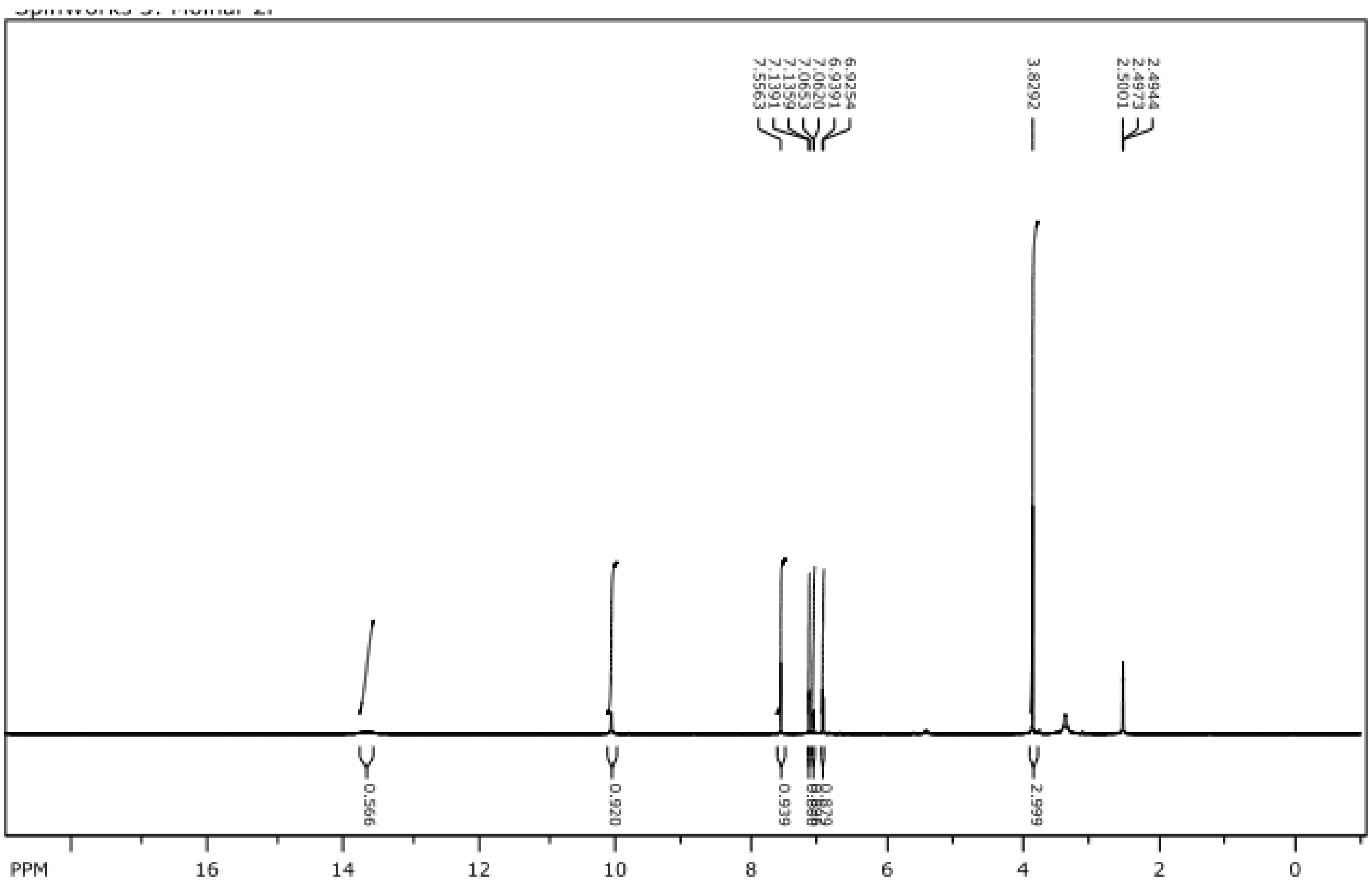

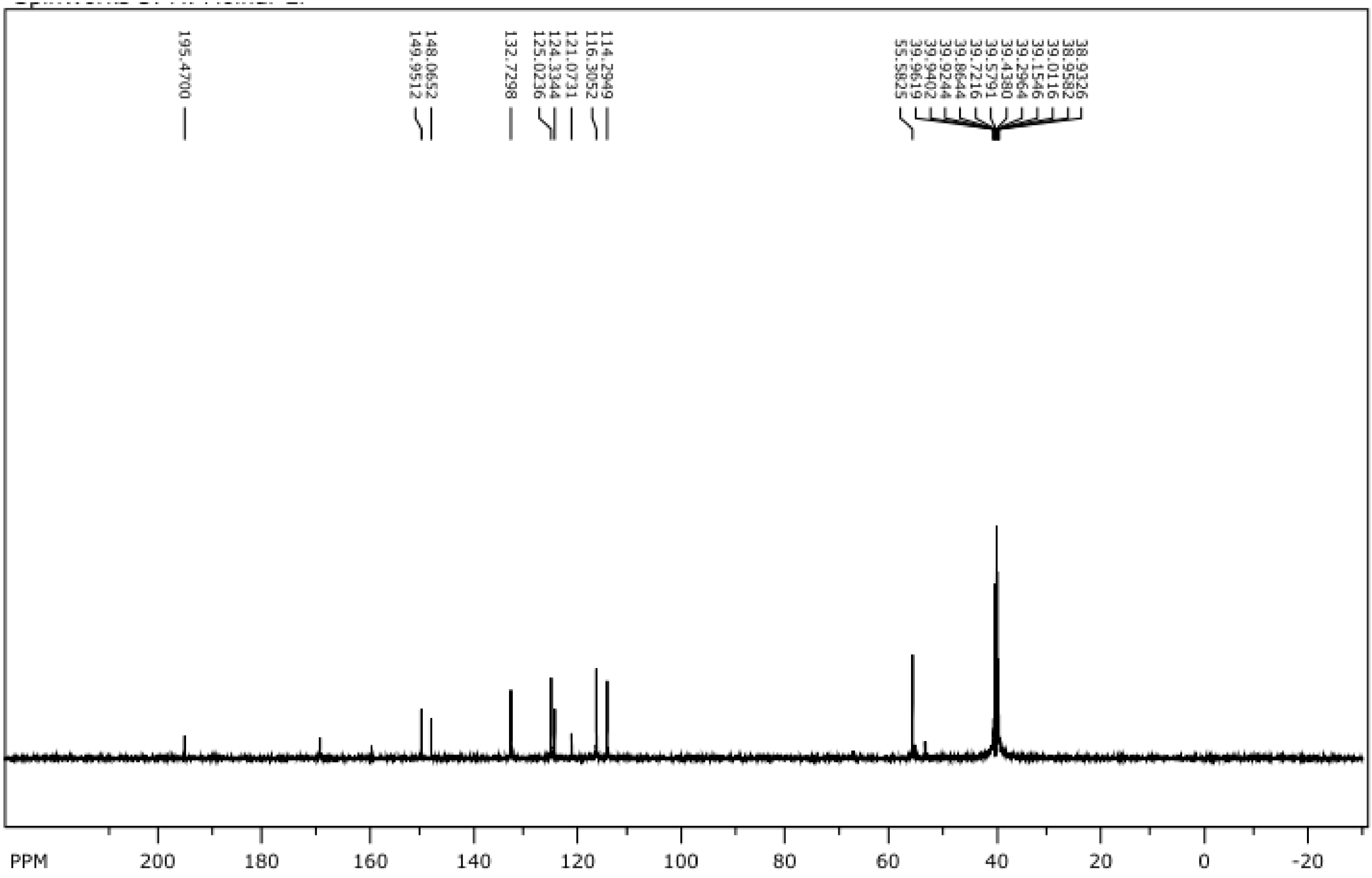
195.4700
148.0652
149.9512
132.7298
125.0236
124.3344
121.0731
116.3052
114.2949
55.5825
39.9619
39.9402
39.9244
39.8644
39.7216
39.5791
39.4380
39.2964
39.1546
39.0116
38.9582
38.9326
PPM
200
180
160
140
120
100
80
60
40
20
0
-20

-Q1: 60 MCA scans from Sample 1 (040_2i) of 040_2i.wiff (Turbo Spray) Max. 3.2e7 cps.

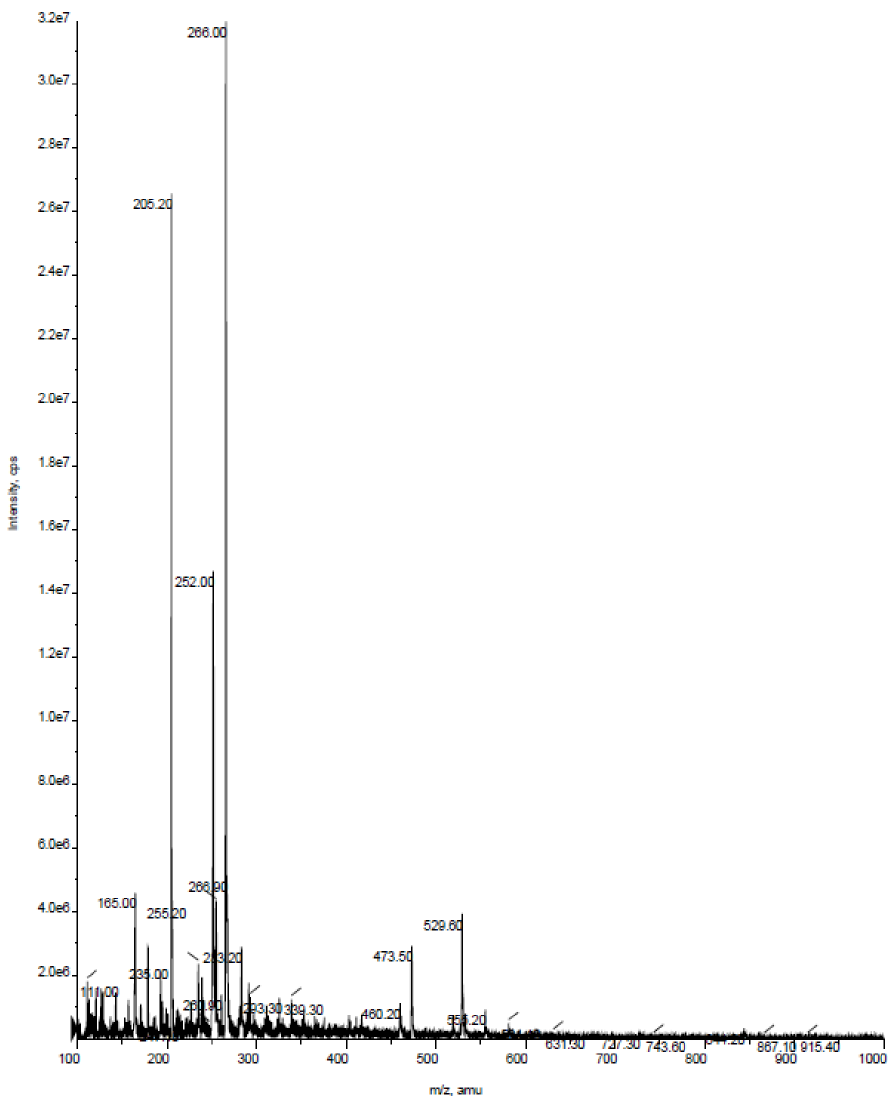

(*Z*)-5-(2-hydroxybenzylidene)-2-thioxothiazolidin-4-one (2g)

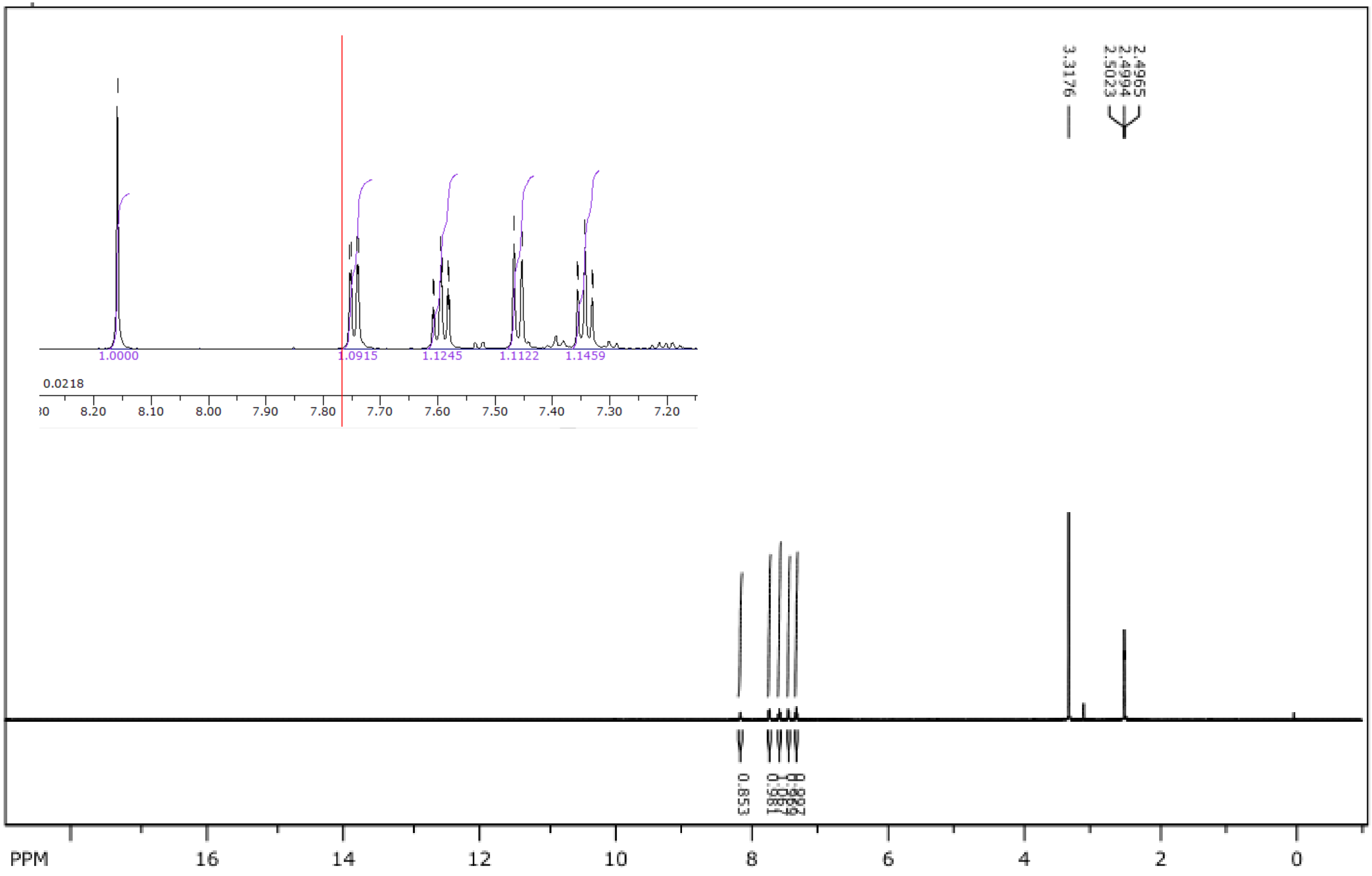

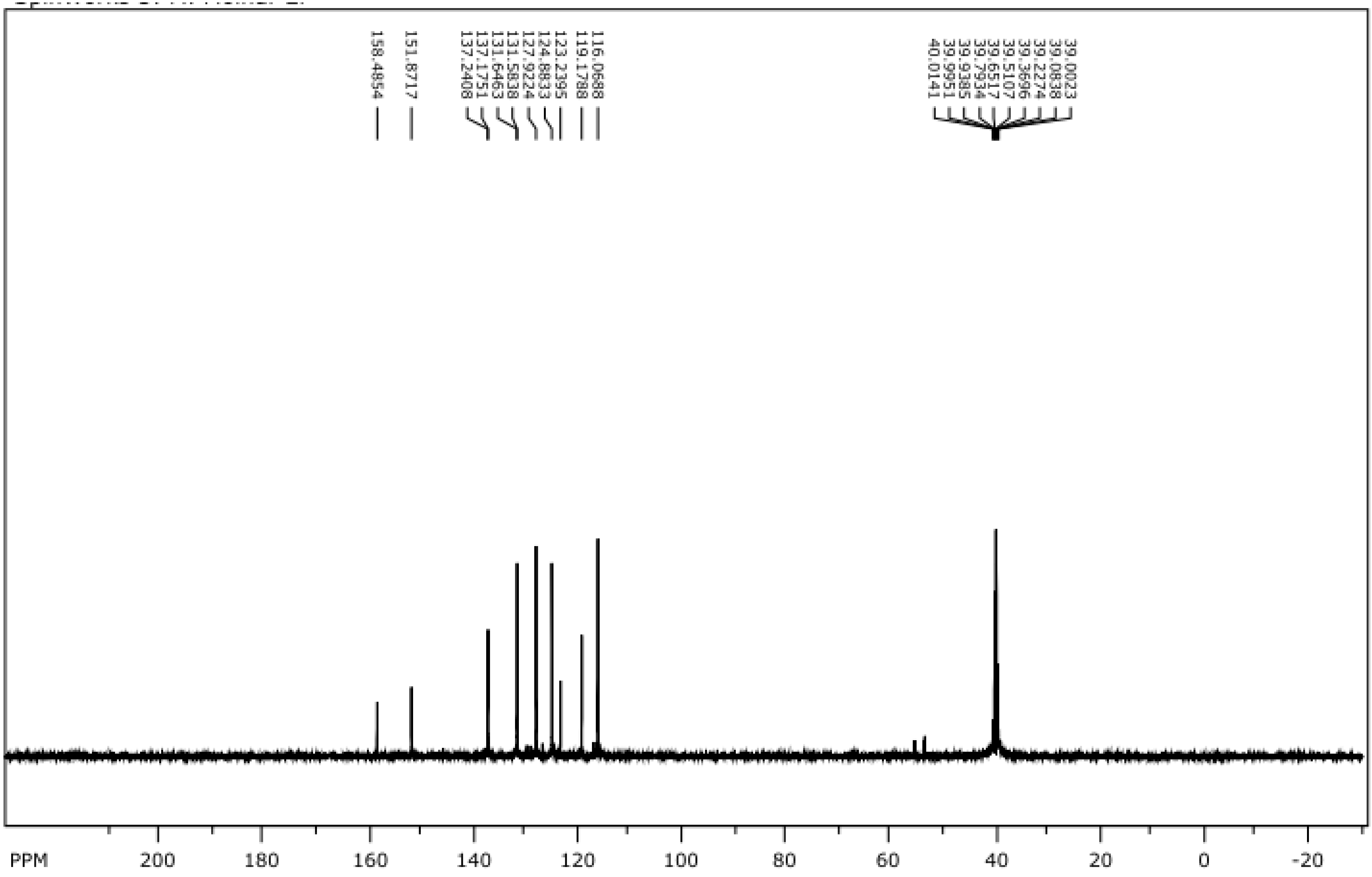
158.4854
151.8717
137.2408
137.1751
131.6463
131.5838
127.9224
124.8833
123.2395
119.1788
116.0688
40.0141
39.9951
39.9385
39.7934
39.6517
39.5107
39.3696
39.2274
39.0838
39.0023
PPM
200
180
160
140
120
100
80
60
40
20
0
-20

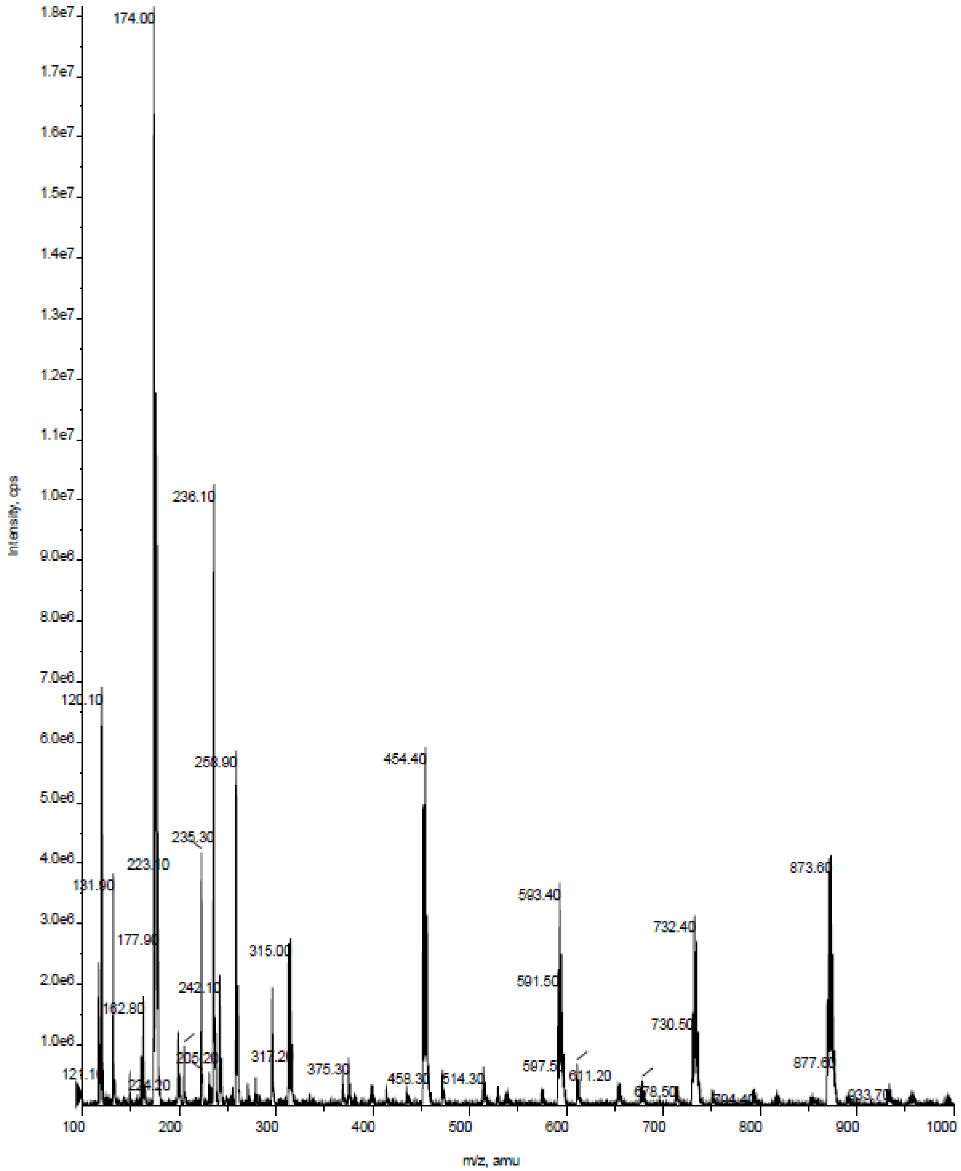

174.00
236.10
120.10
258.90
454.40
235.30
223.10
131.90
873.60
593.40
732.40
177.90
315.00
242.10
591.50
162.80
730.50
205.20
317.20
375.30
459.30
514.30
597.50
611.20
877.60
121.10
224.20
678.50
933.70
m/z, amu
Intensity, cps

(*Z*)-5-benzylidene-2-thioxothiazolidin-4-one (2h)

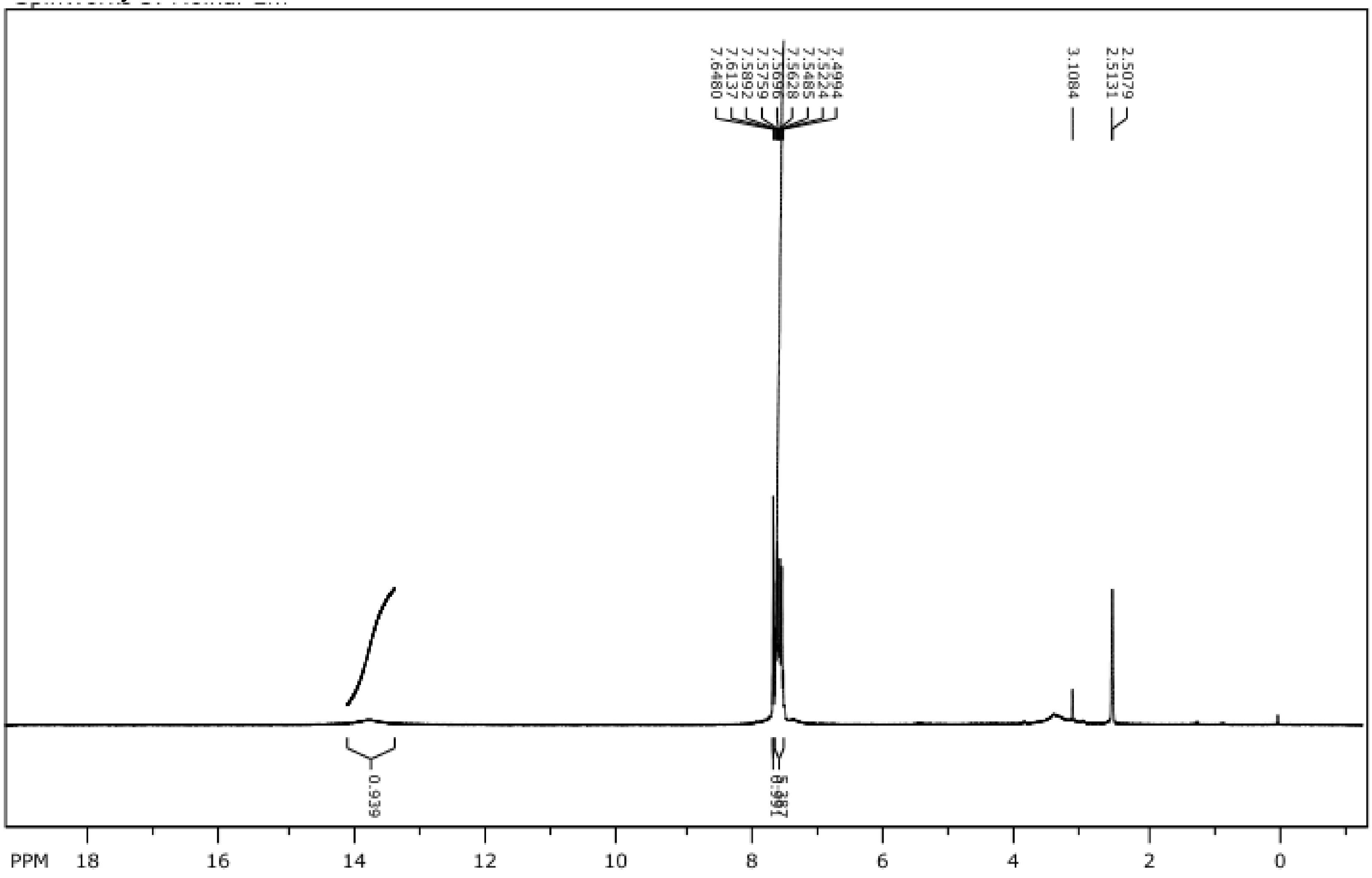

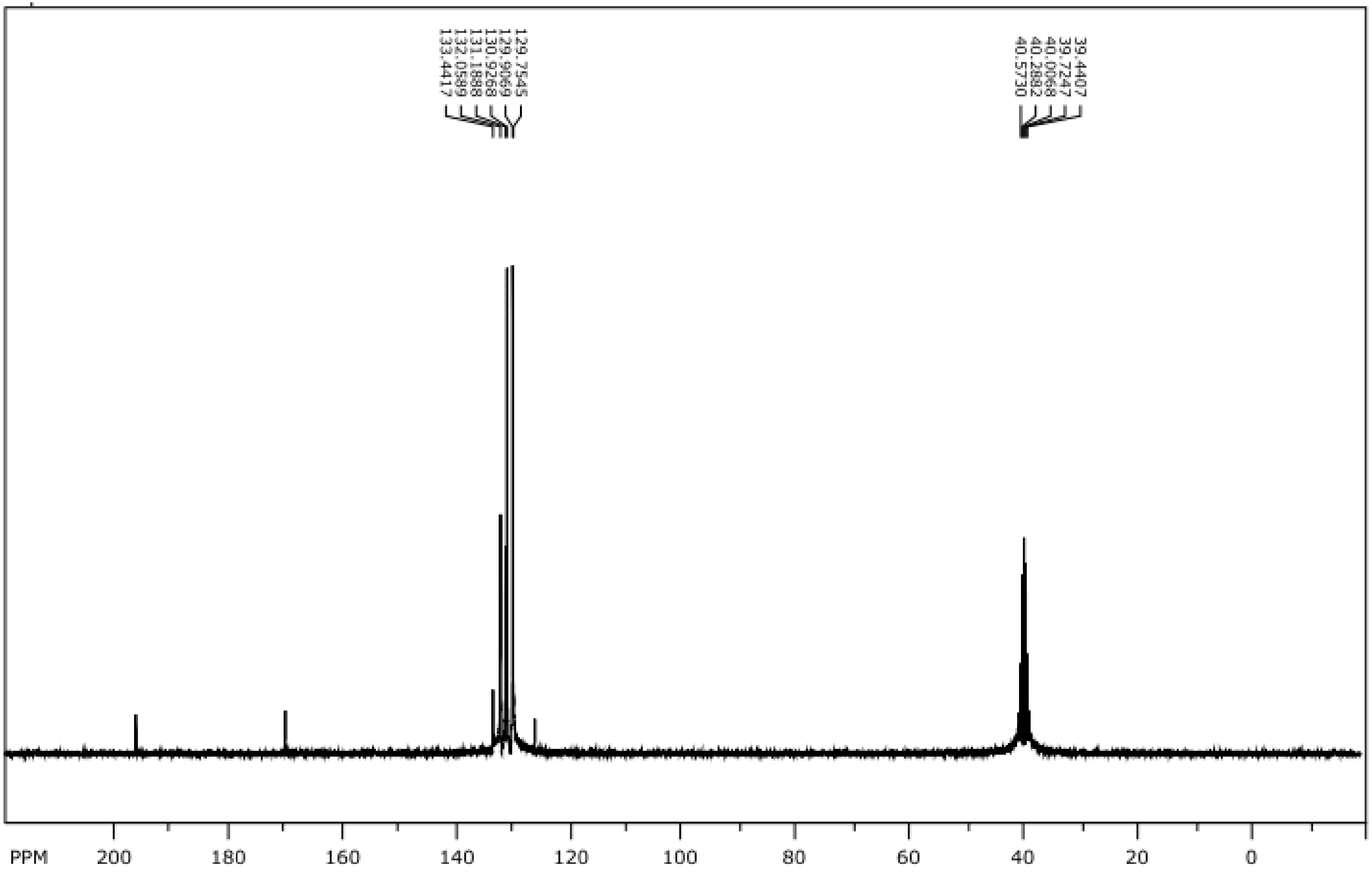
133.4417
132.0589
131.1888
130.9268
129.9069
129.7545
40.5730
40.2882
40.0068
39.7247
39.4407
PPM
200
180
160
140
120
100
80
60
40
20
0

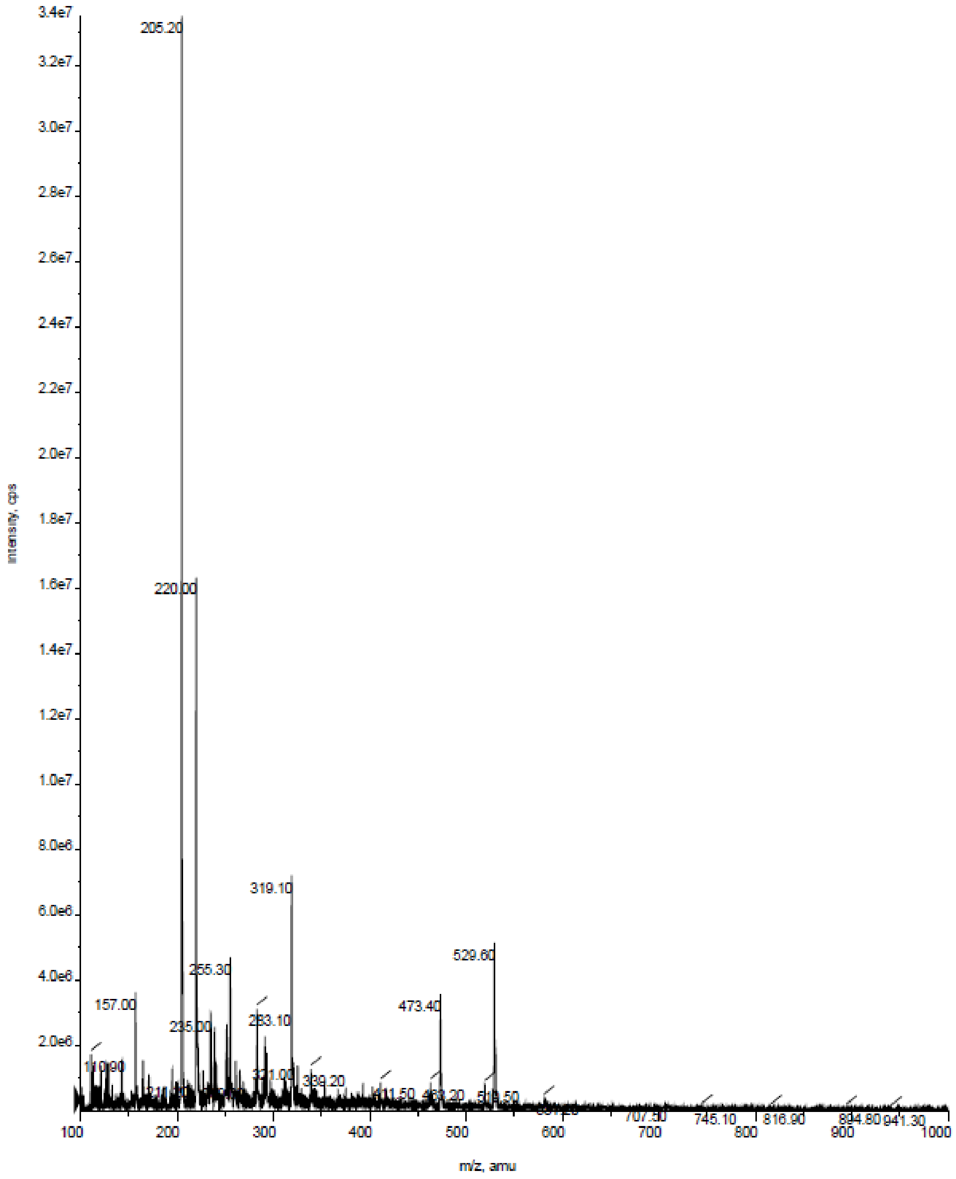
Intensity, cps
m/z, amu
3.4e7
3.2e7
3.0e7
2.8e7
2.6e7
2.4e7
2.2e7
2.0e7
1.8e7
1.6e7
1.4e7
1.2e7
1.0e7
8.0e6
6.0e6
4.0e6
2.0e6
100
200
300
400
500
600
700
800
900
1000
205.20
220.00
319.10
529.60
255.30
157.00
473.40
235.00
283.10
745.10
816.90
941.30

(*Z*)-5-(pyridin-4-ylmethylene)-2-thioxothiazolidin-4-one (2i)

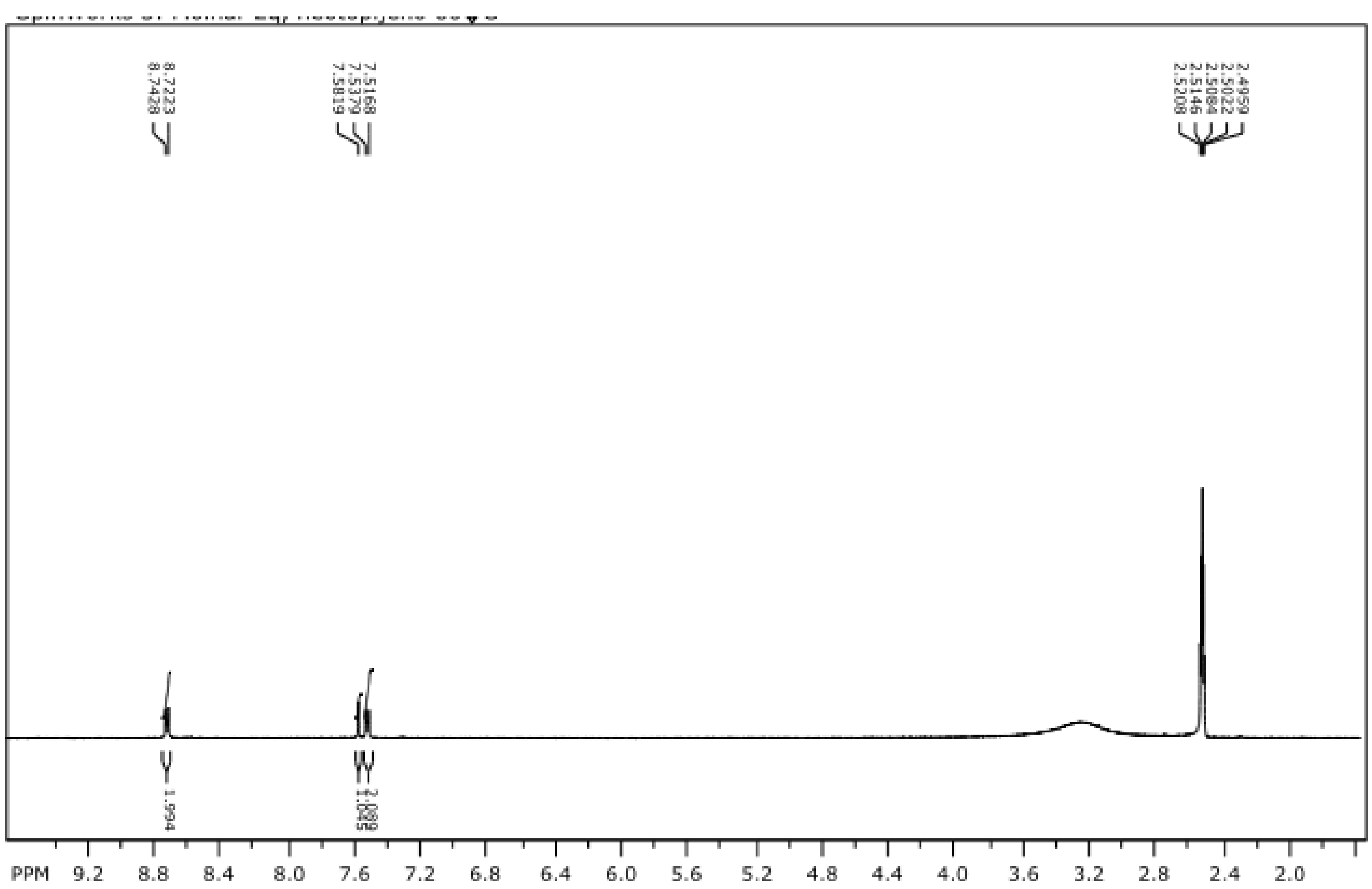

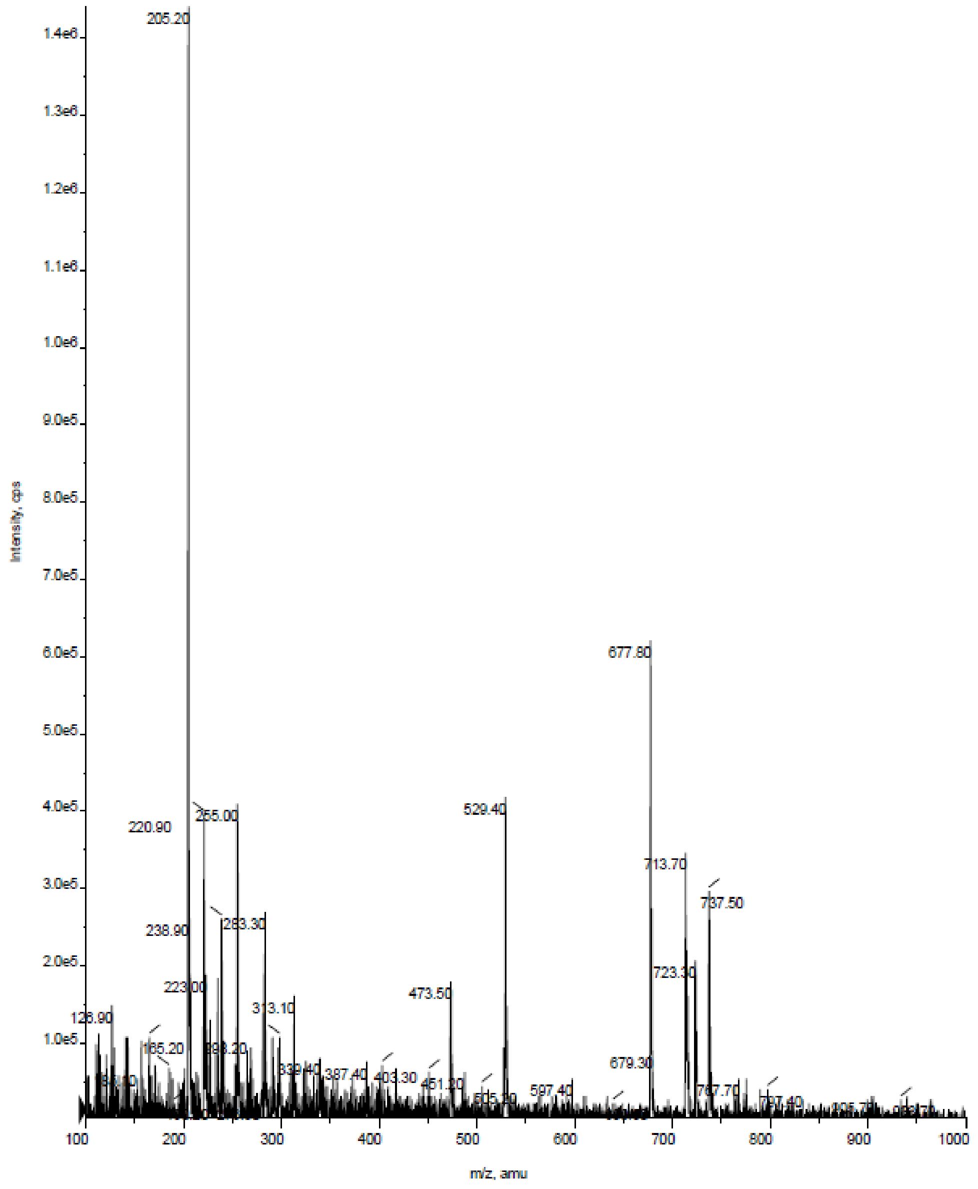

205.20
677.80
529.40
255.00
220.90
713.70
737.50
263.30
238.90
723.30
223.00
473.50
313.10
126.90
165.20
679.30
339.40
387.40
403.30
451.20
597.40
Intensity, cps
m/z, amu
1.4e6
1.3e6
1.2e6
1.1e6
1.0e6
9.0e5
8.0e5
7.0e5
6.0e5
5.0e5
4.0e5
3.0e5
2.0e5
1.0e5
100
200
300
400
500
600
700
800
900
1000

(*Z*)-5-(2,5-dimethoxybenzylidene)-2-thioxothiazolidin-4-one (2j)

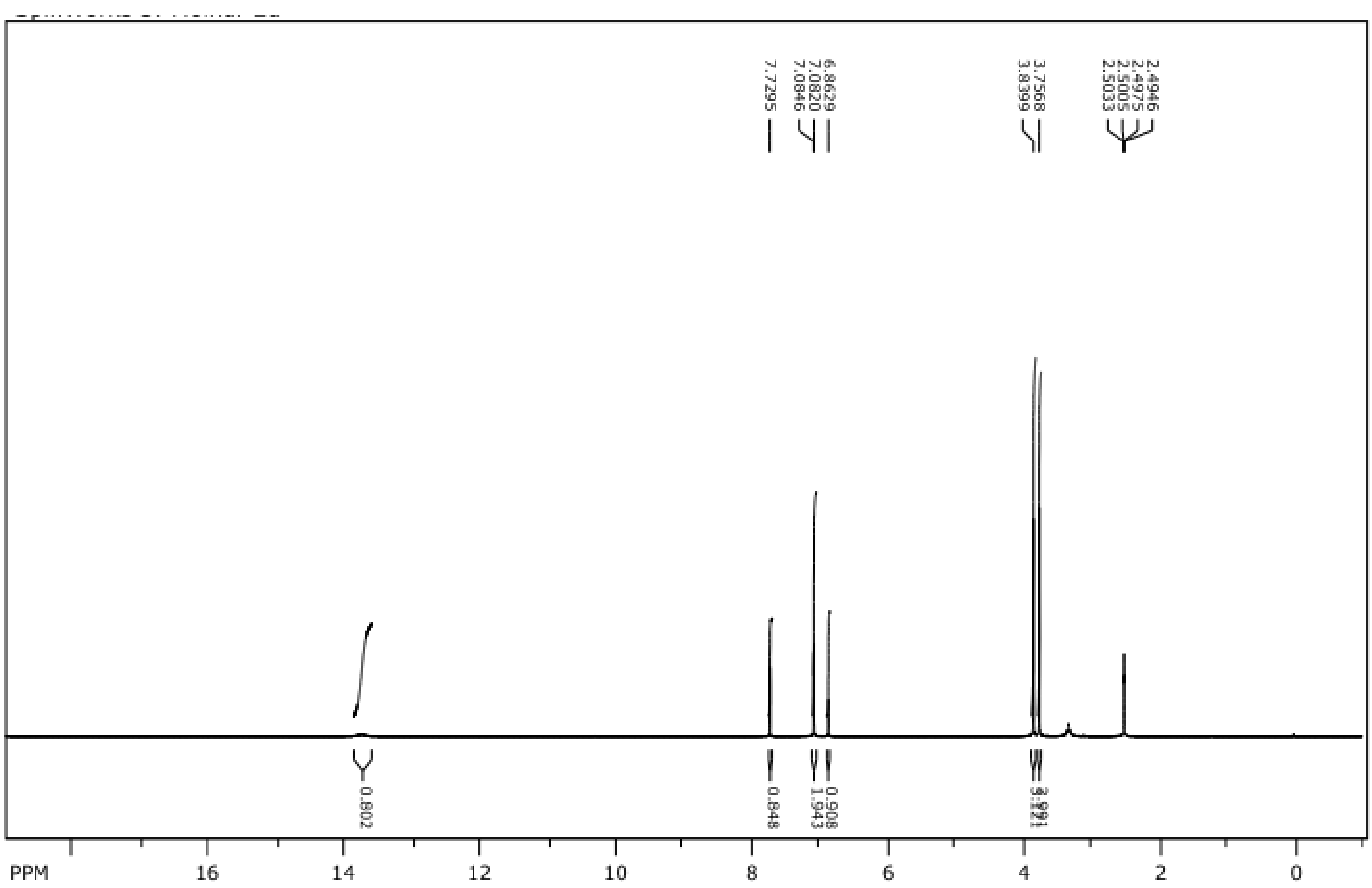

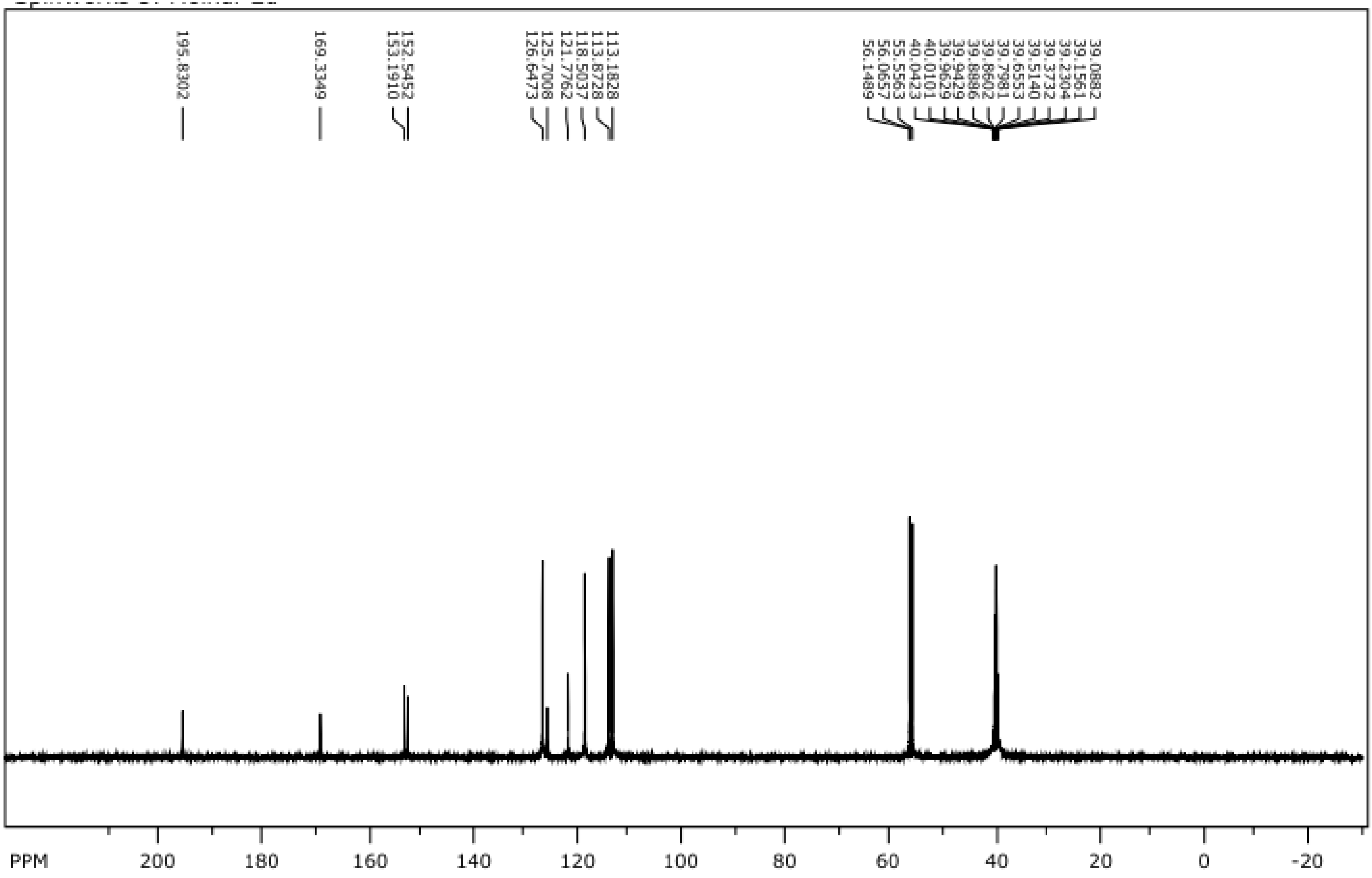
195.8302
169.3349
152.5452
153.1910
126.6473
125.7008
121.7762
118.5037
113.8728
113.1828
56.1489
56.0657
55.5563
40.0423
40.0101
39.9629
39.9429
39.8886
39.8602
39.7981
39.6553
39.5140
39.3732
39.2304
39.1561
39.0882
PPM
200
180
160
140
120
100
80
60
40
20
0
-20

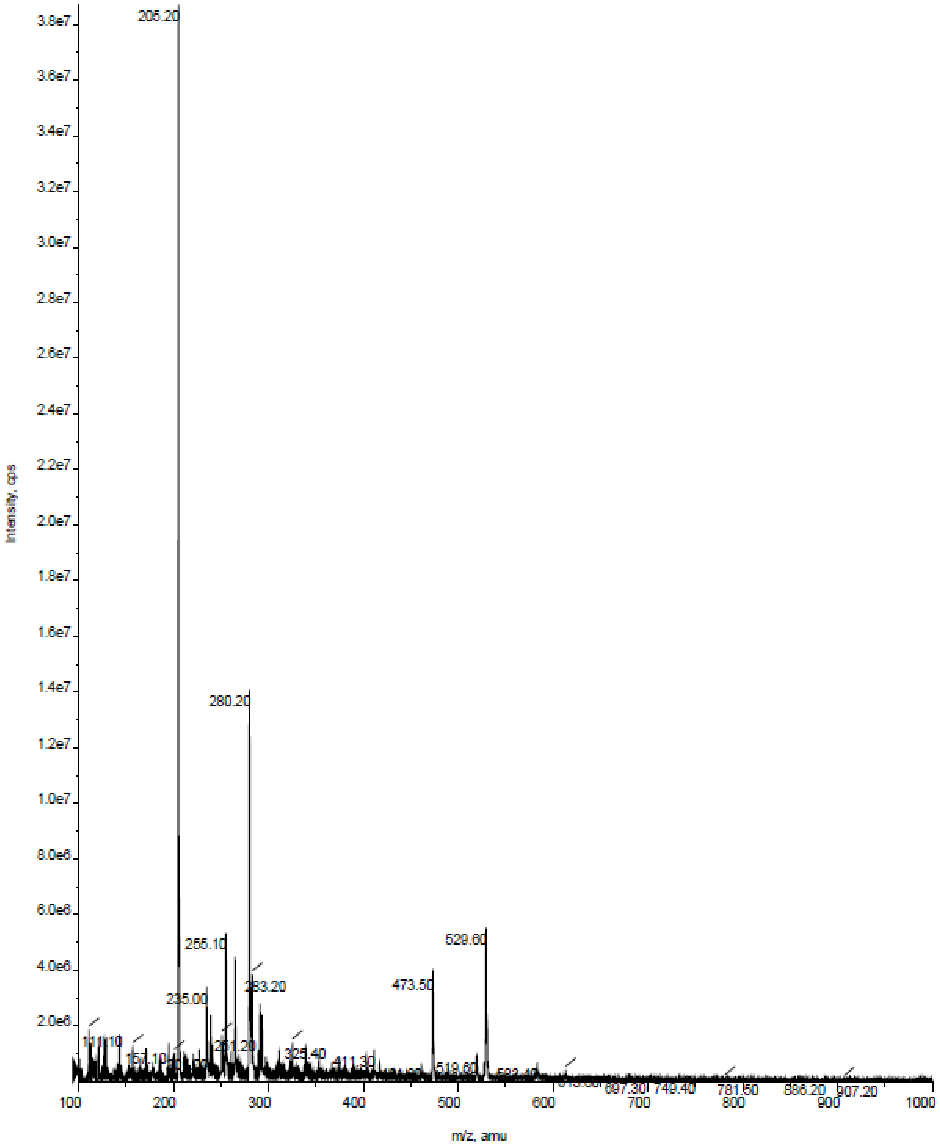
205.20
280.20
255.10
235.00
283.20
473.50
529.60
Intensity, cps
m/z, amu
100
200
300
400
500
600
700
800
900
1000
3.8e7
3.6e7
3.4e7
3.2e7
3.0e7
2.8e7
2.6e7
2.4e7
2.2e7
2.0e7
1.8e7
1.6e7
1.4e7
1.2e7
1.0e7
8.0e6
6.0e6
4.0e6
2.0e6

(*Z*)-5-(4-hydroxybenzylidene)-2-thioxothiazolidin-4-one (2k)

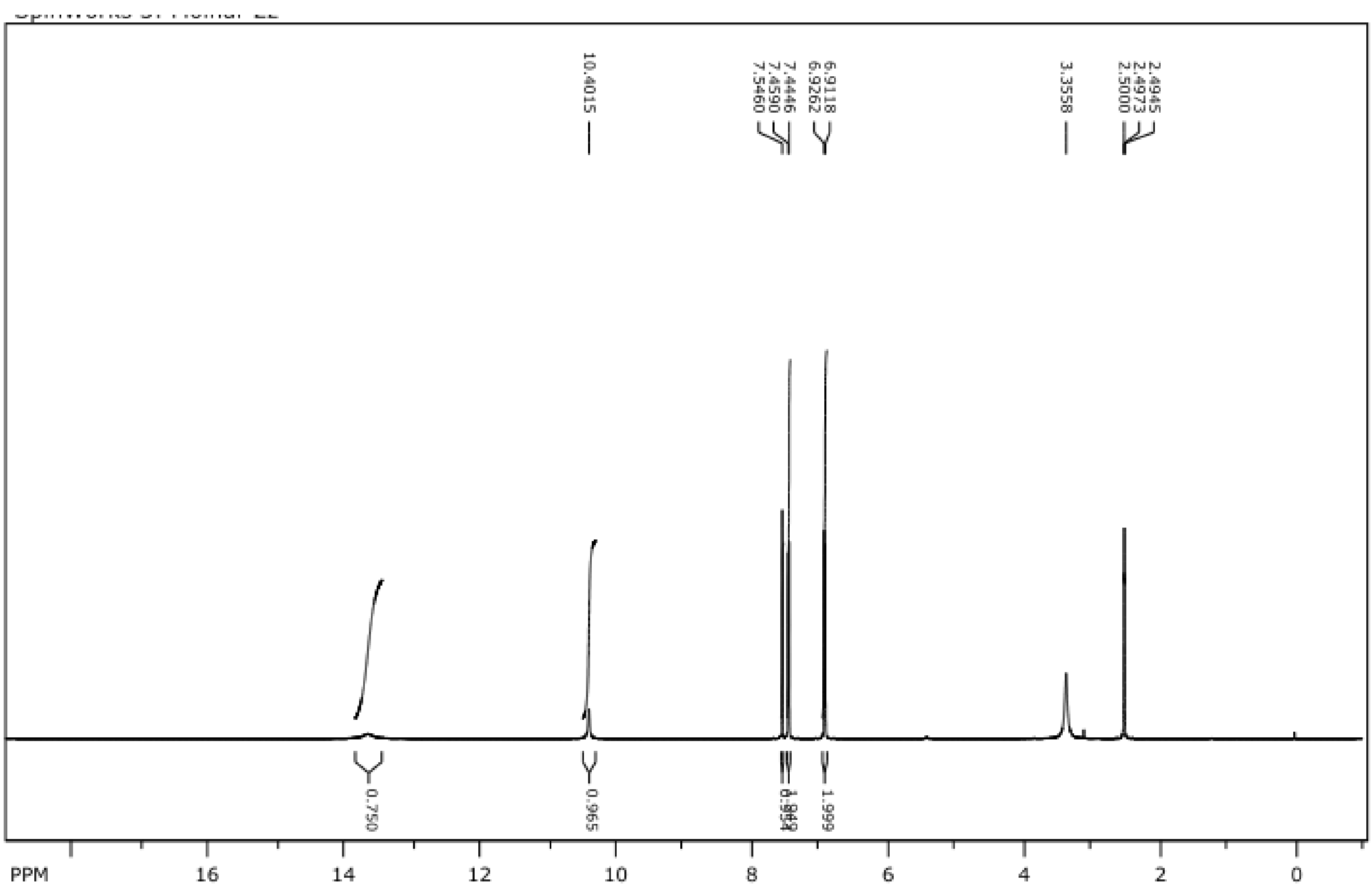

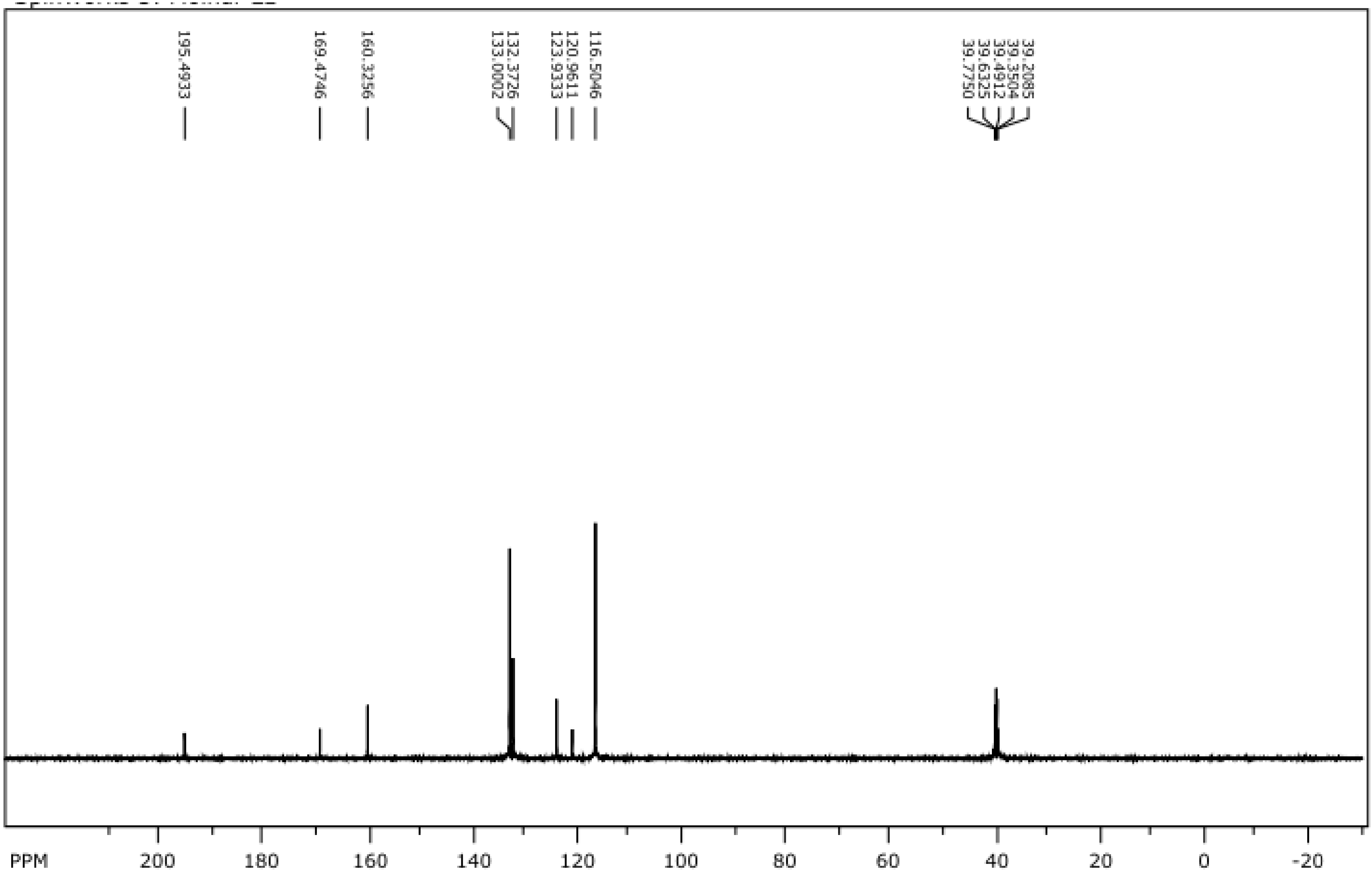

195.4933
169.4746
160.3256
132.3726
133.0002
123.9333
120.9611
116.5046
39.2085
39.3504
39.4912
39.6325
39.7750
PPM
200
180
160
140
120
100
80
60
40
20
0
-20

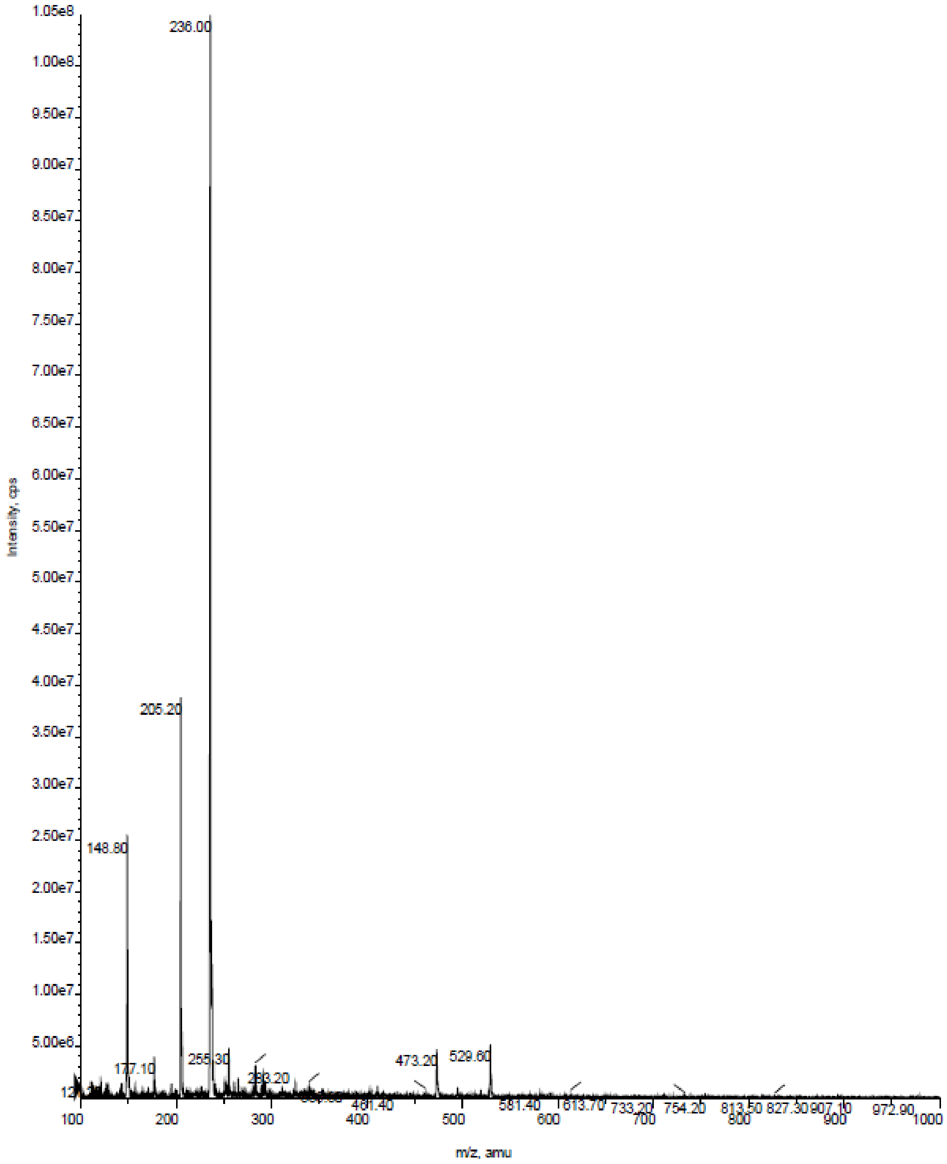

1.05e8
1.00e8
9.50e7
9.00e7
8.50e7
8.00e7
7.50e7
7.00e7
6.50e7
6.00e7
5.50e7
5.00e7
4.50e7
4.00e7
3.50e7
3.00e7
2.50e7
2.00e7
1.50e7
1.00e7
5.00e6
Intensity, cps
236.00
205.20
148.80
177.10
255.30
473.20
529.60
581.40
613.70
733.20
754.20
813.50
827.30
907.10
972.90
100
200
300
400
500
600
700
800
900
1000
m/z, amu

(*Z*)-3-(4-chlorophenyl)-5-(4-hydroxy-3-methoxybenzylidene)-2-thioxothiazolidin-4-one (2l)

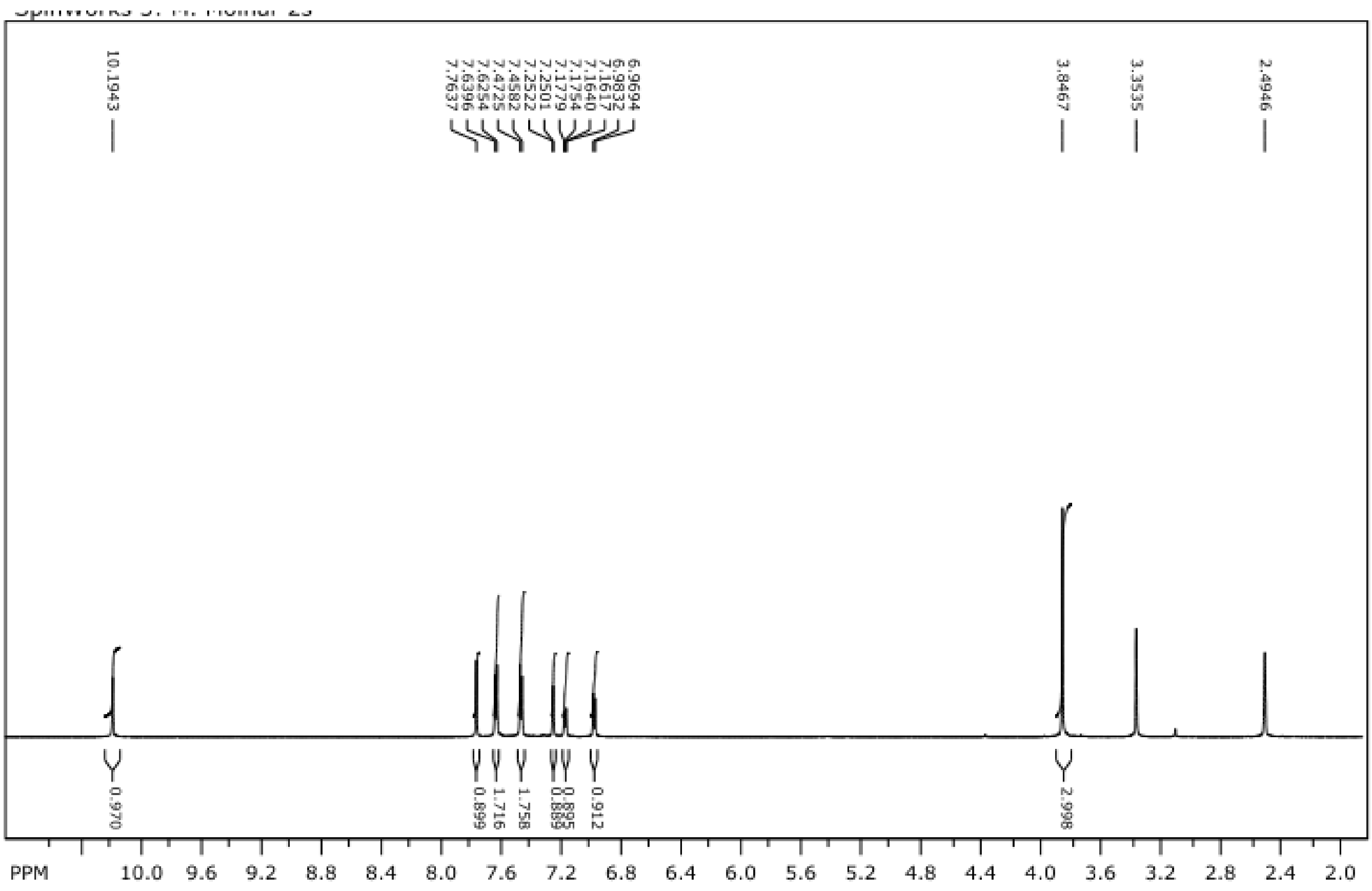

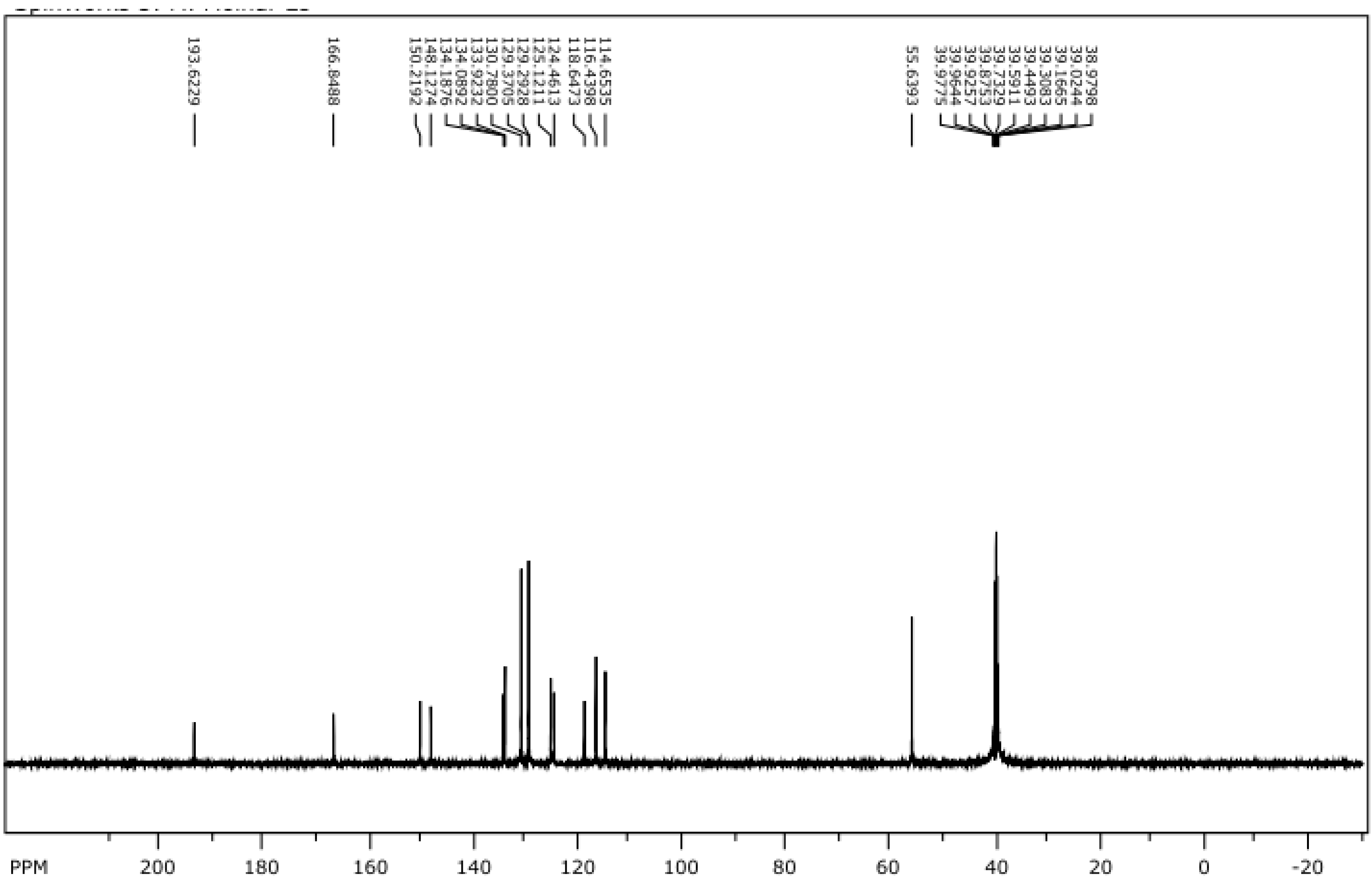
193.6229
166.8488
150.2192
148.1274
134.1876
134.0892
133.9232
130.7800
129.3705
129.2928
125.1211
124.4613
118.6473
116.4398
114.6535
55.6393
39.9775
39.9644
39.9257
39.8753
39.7329
39.5911
39.4493
39.3083
39.1665
39.0244
38.9798
PPM
200
180
160
140
120
100
80
60
40
20
0
-20

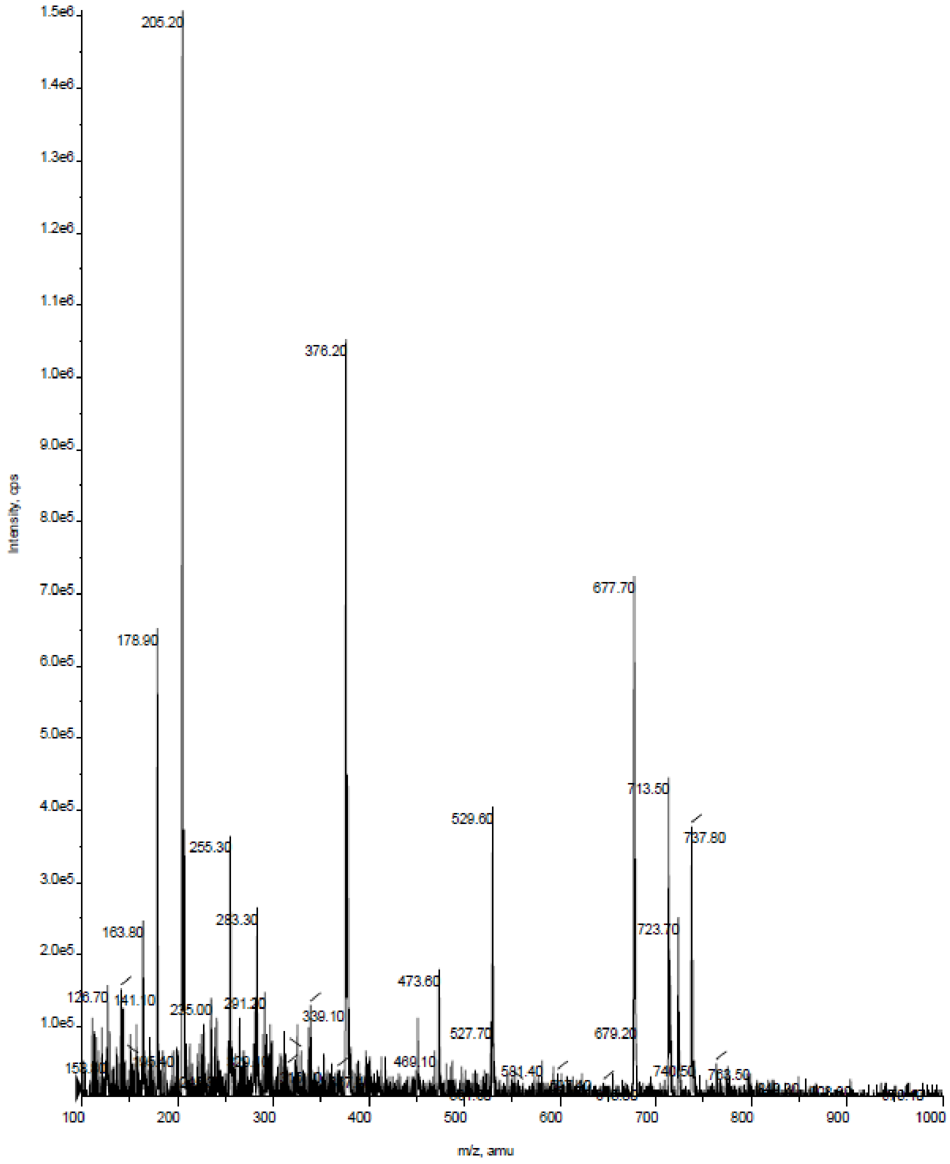

Intensity, cps
m/z, amu
205.20
376.20
677.70
178.90
713.50
737.80
529.60
255.30
283.30
723.70
163.80
473.60
126.70
141.10
235.00
291.20
339.10
527.70
679.20
469.10
591.40
740.50

(*Z*)-3-(4-chlorophenyl)-5-(4-(dimethylamino)benzylidene)-2-thioxothiazolidin-4-one (2m)

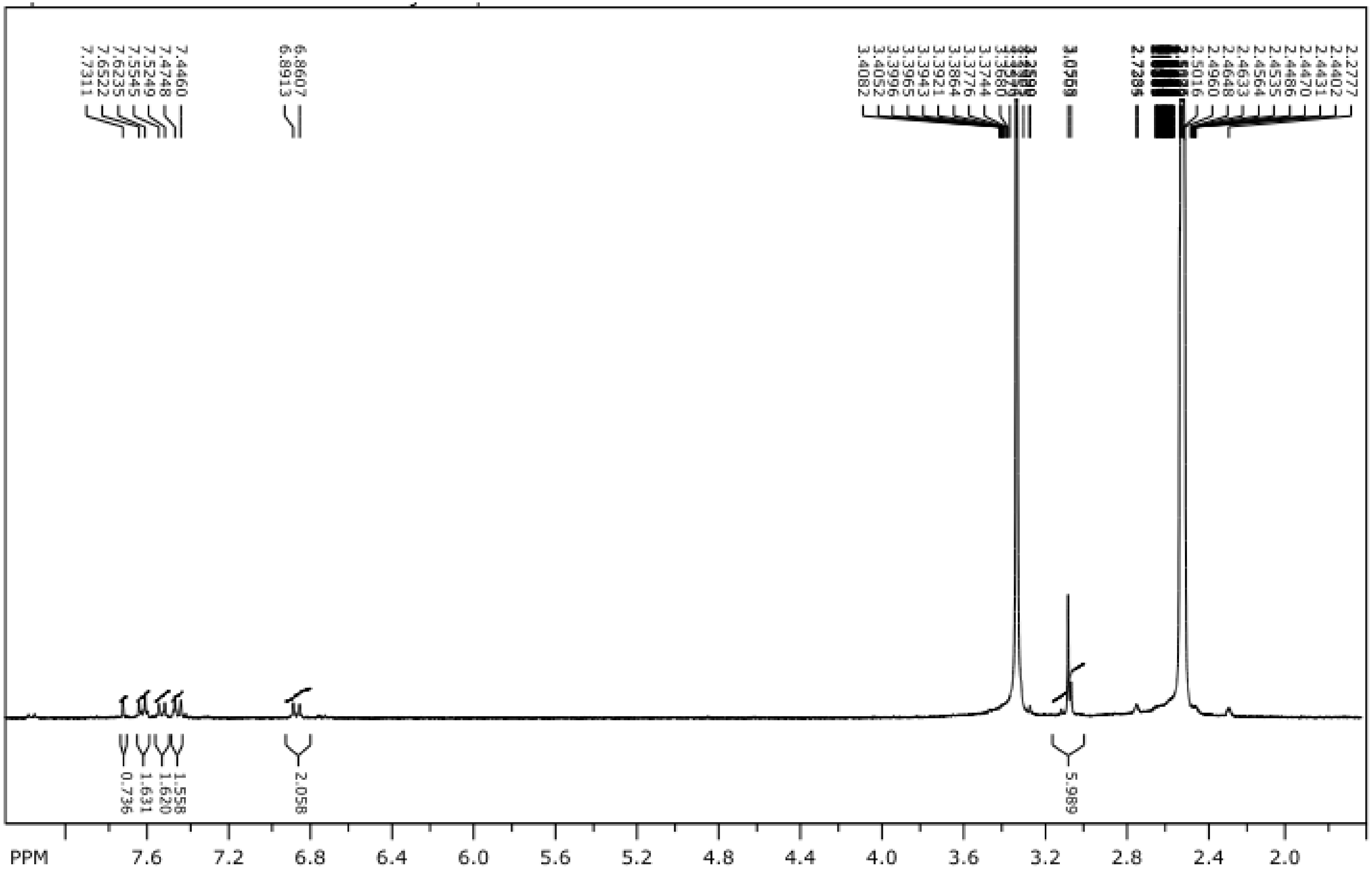

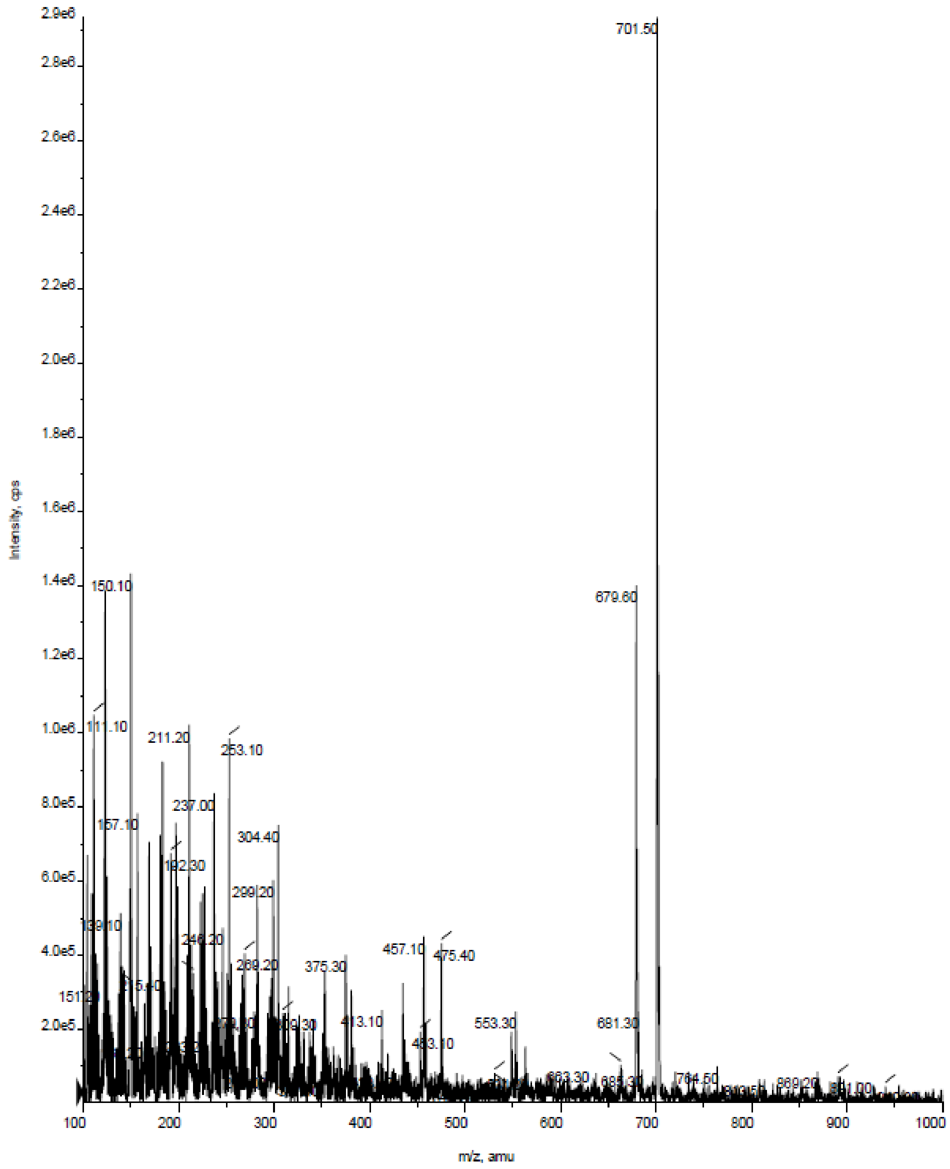
701.50
679.60
150.10
111.10
211.20
253.10
237.00
157.10
304.40
192.30
299.20
139.10
457.10
475.40
375.30
553.30
681.30
Intensity, cps
m/z, amu
2.9e6
2.8e6
2.6e6
2.4e6
2.2e6
2.0e6
1.8e6
1.6e6
1.4e6
1.2e6
1.0e6
8.0e5
6.0e5
4.0e5
2.0e5
100
200
300
400
500
600
700
800
900
1000

(*Z*)-3-(4-chlorophenyl)-5-(4-hydroxybenzylidene)-2-thioxothiazolidin-4-one (2n)

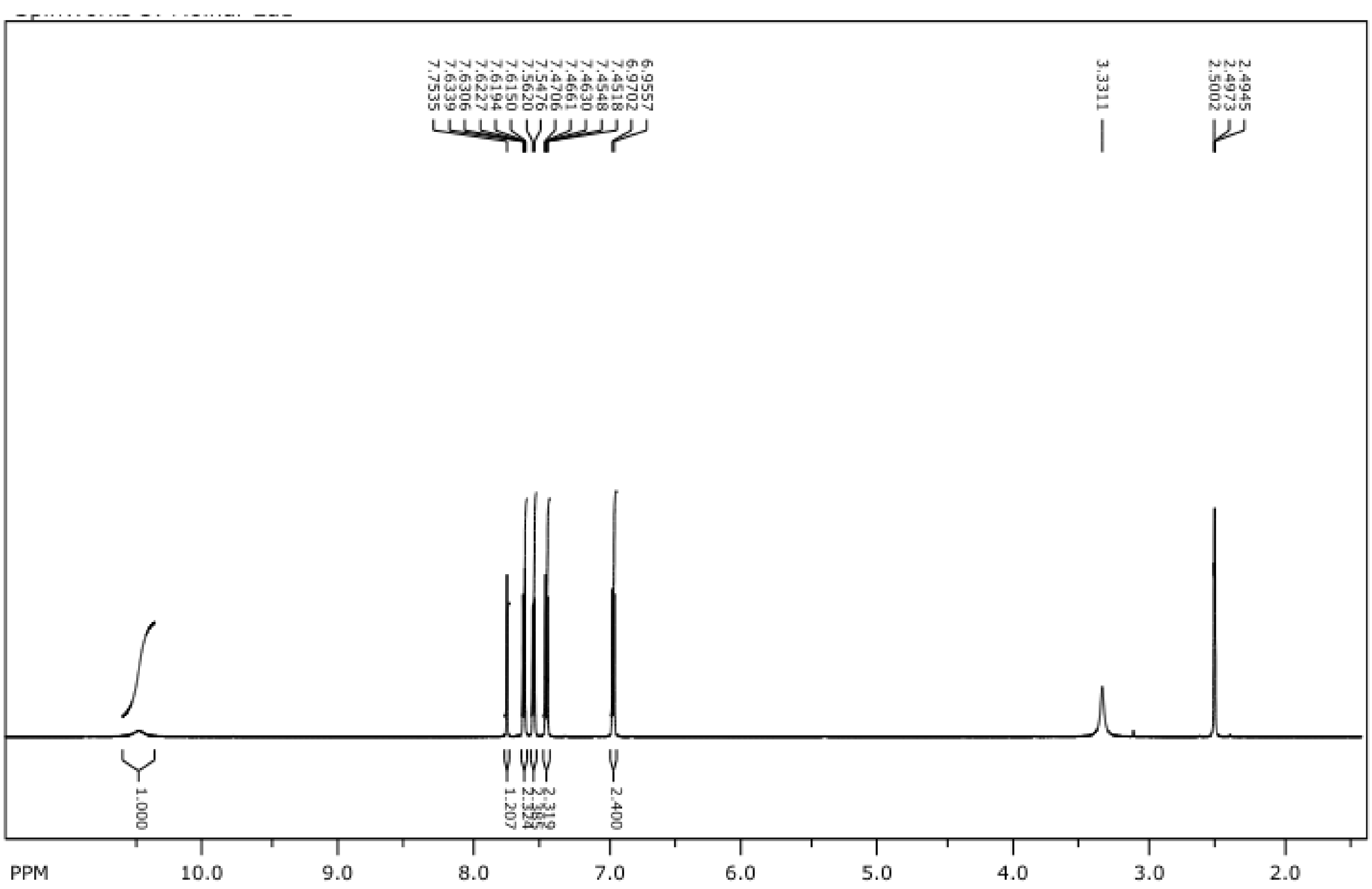

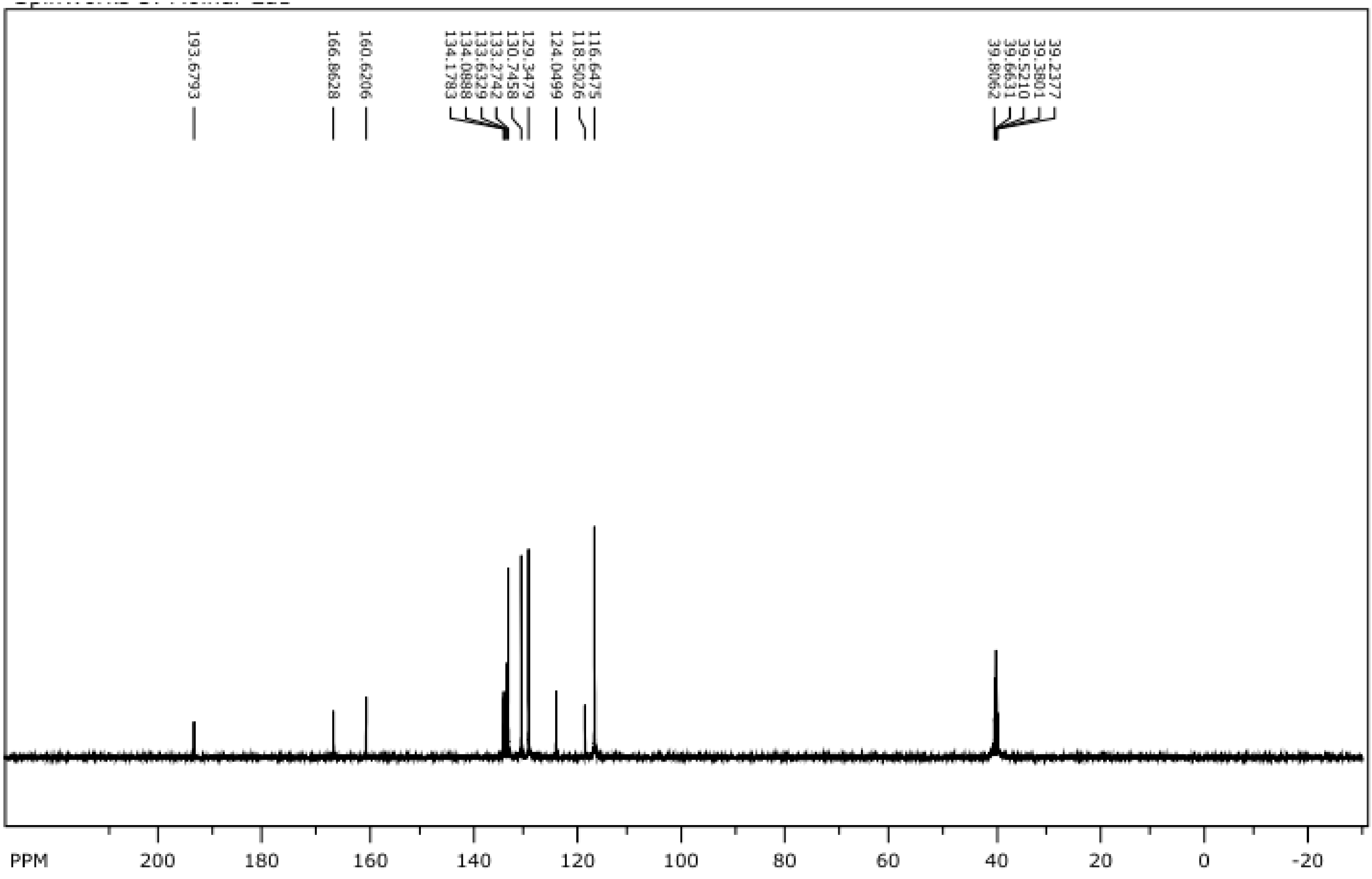
193.6793
166.8628
160.6206
134.1783
134.0888
133.6329
133.2742
130.7458
129.3479
124.0499
118.5026
116.6475
39.8062
39.6631
39.5210
39.3801
39.2377
PPM
200
180
160
140
120
100
80
60
40
20
0
-20

-Q1: 60 MCA scans from Sample 1 (054_2a1) of 054_2a1.wiff (Turbo Spray) Max. 4.1e7 cps.

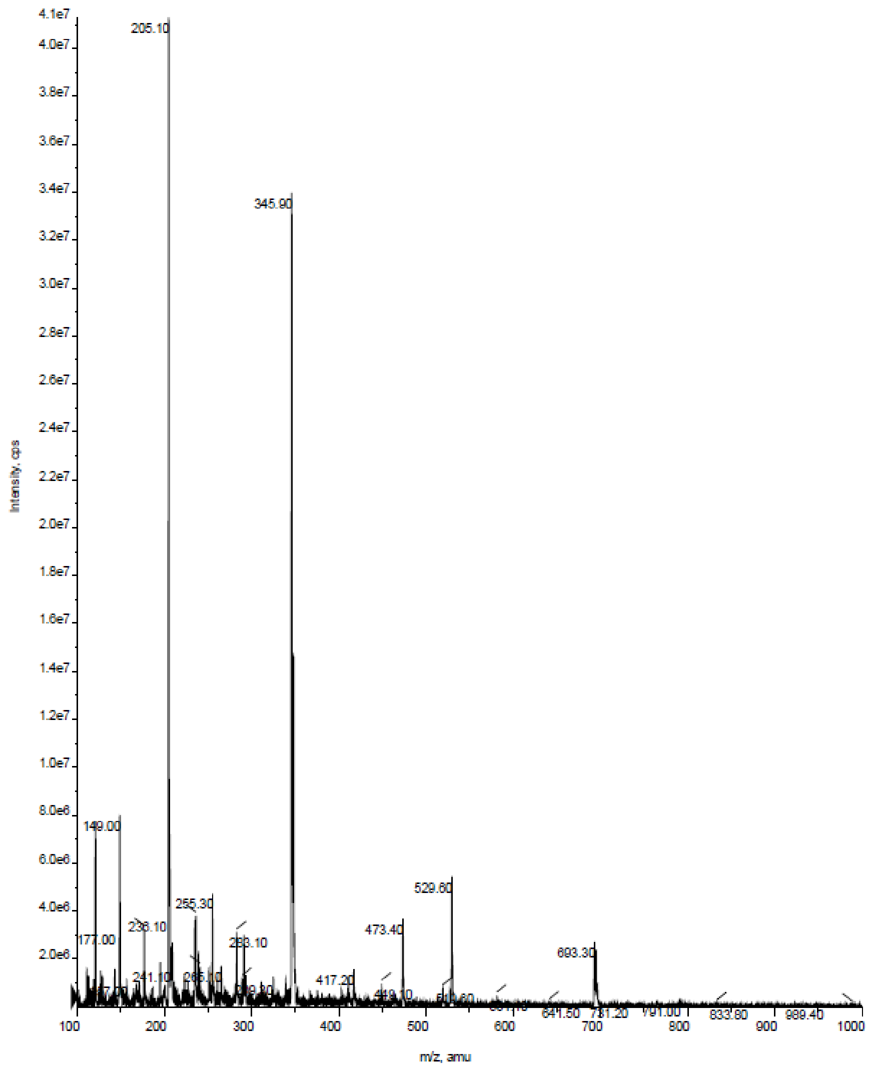

(*Z*)-3-(4-chlorophenyl)-5-(2,5-dimethoxybenzylidene)-2-thioxothiazolidin-4-one (2o)

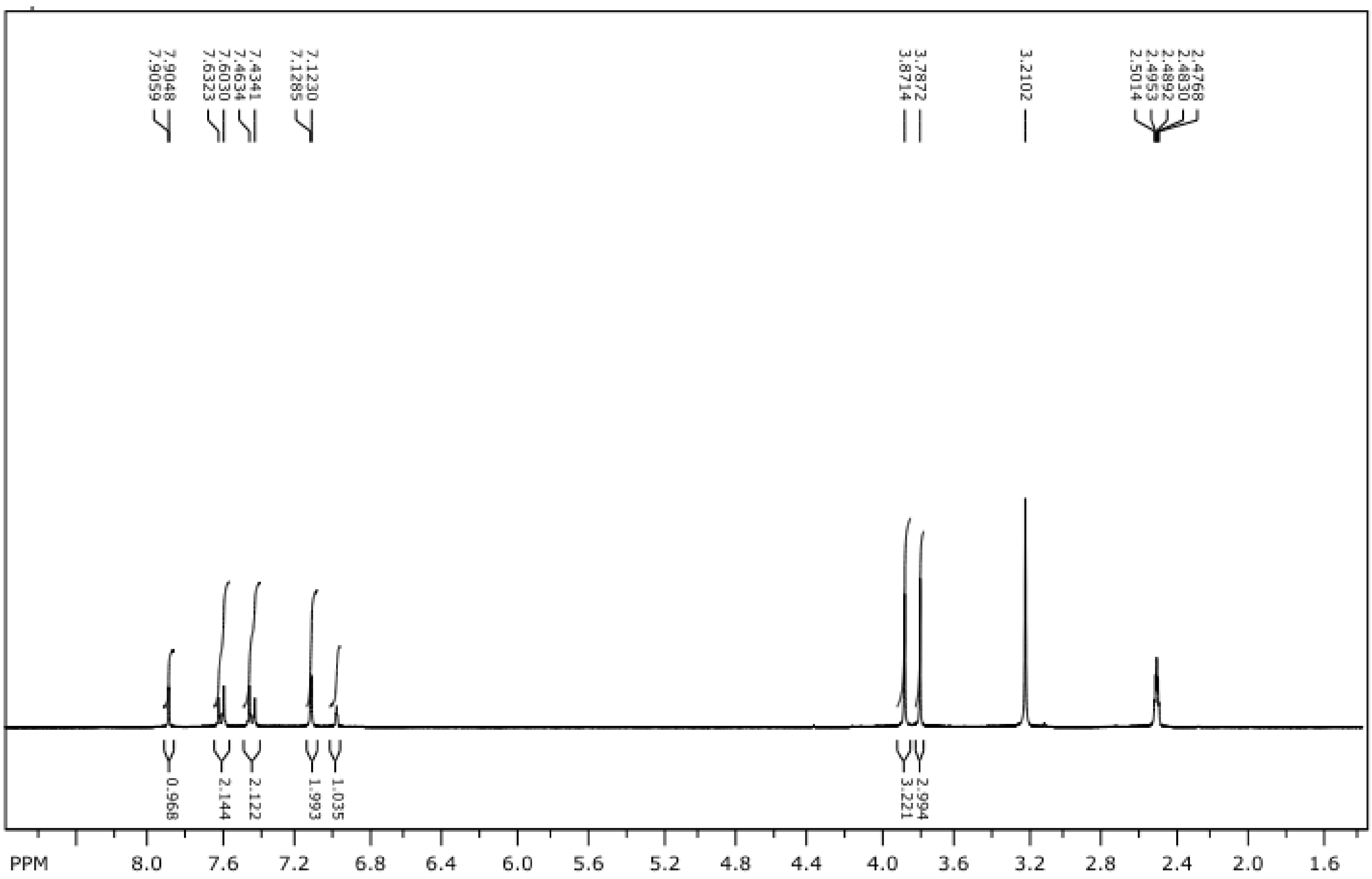

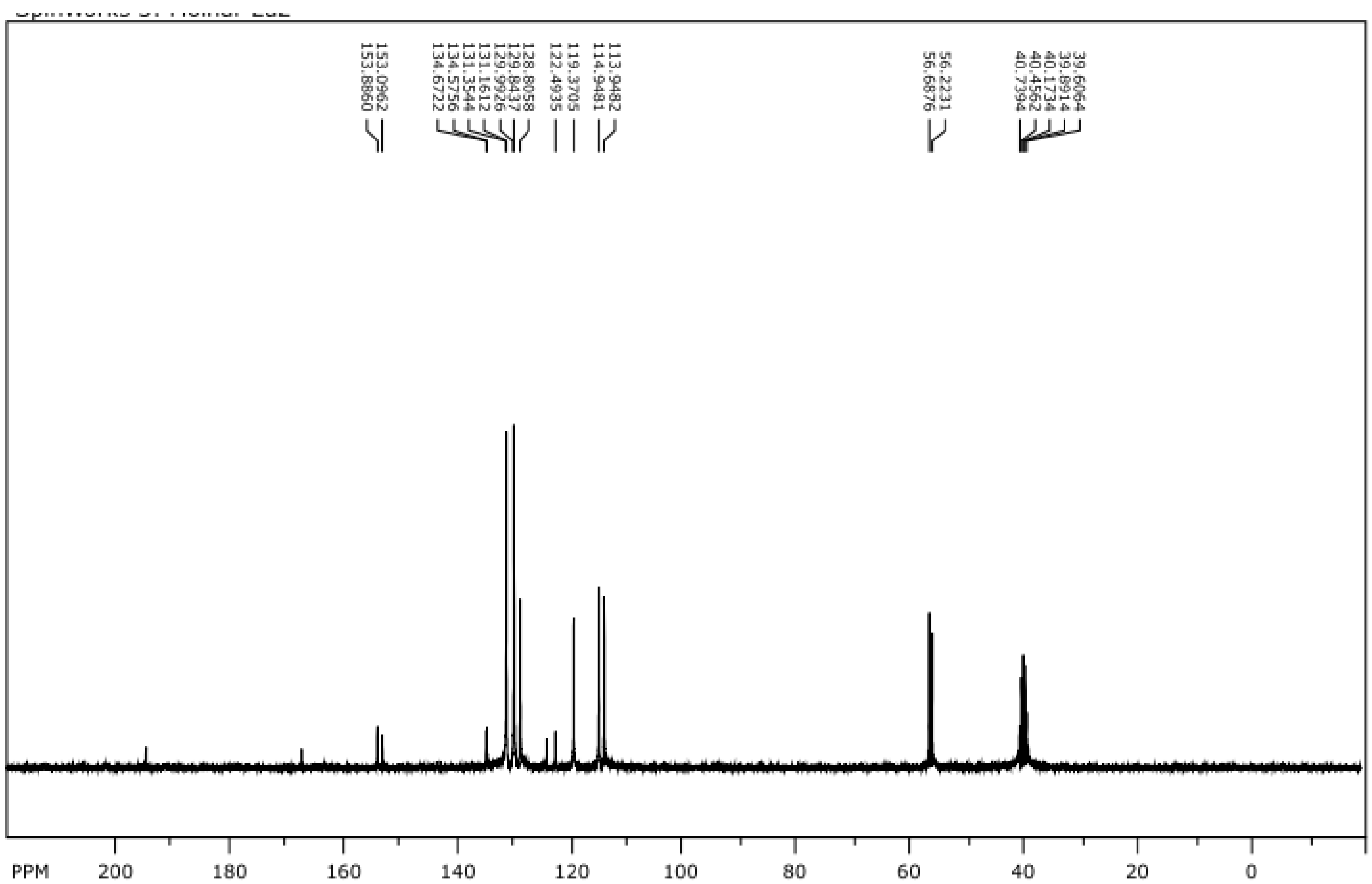
153.0962
153.8860
134.6722
134.5756
131.3544
131.1612
129.9926
129.8437
128.8058
122.4935
119.3705
114.9481
113.9482
56.2231
56.6876
39.6064
39.8914
40.1734
40.4562
40.7394
PPM
200
180
160
140
120
100
80
60
40
20
0

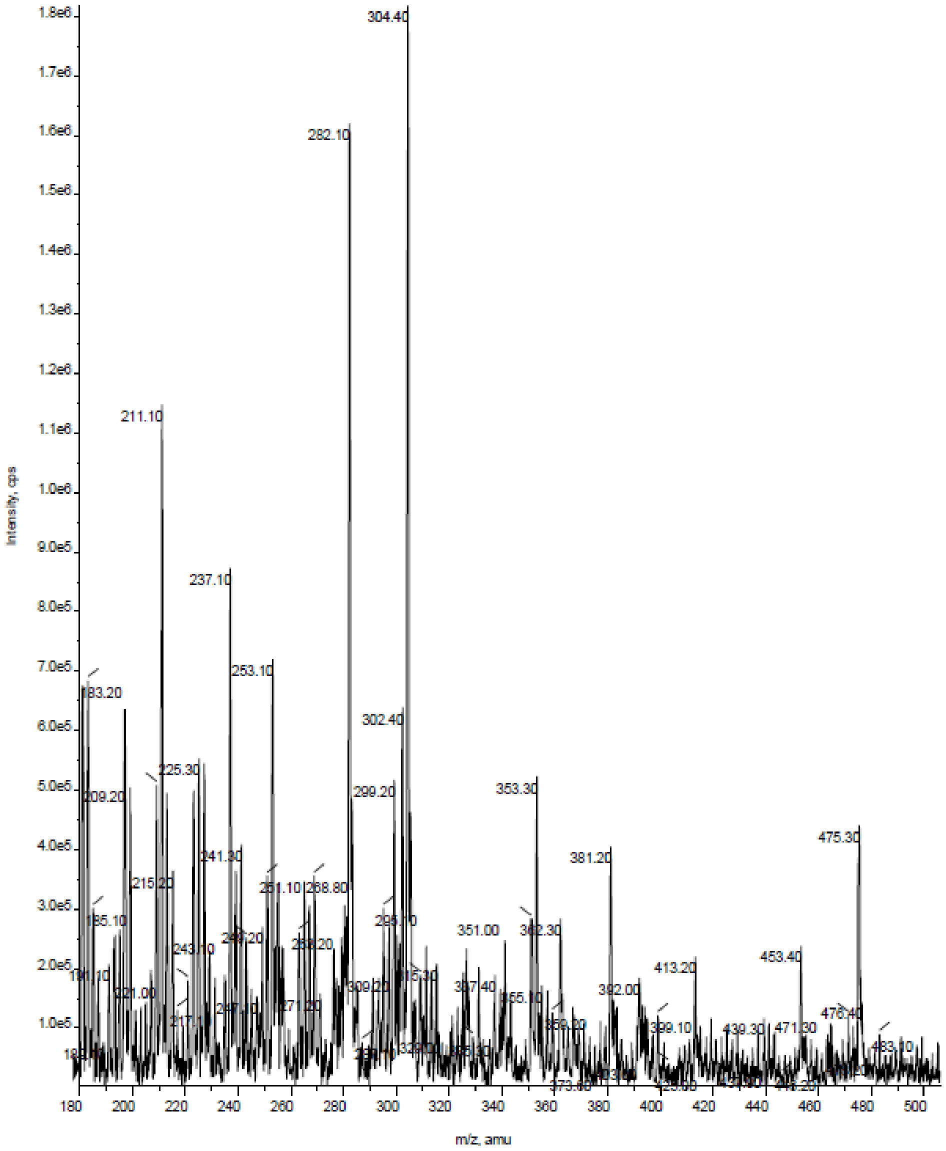

Intensity, cps
1.8e6
1.7e6
1.6e6
1.5e6
1.4e6
1.3e6
1.2e6
1.1e6
1.0e6
9.0e5
8.0e5
7.0e5
6.0e5
5.0e5
4.0e5
3.0e5
2.0e5
1.0e5
304.40
282.10
211.10
237.10
253.10
183.20
302.40
225.30
209.20
299.20
353.30
475.30
381.20
241.30
215.20
251.10
268.80
351.00
362.30
413.20
453.40
392.00
399.10
439.30
471.30
476.40
180
200
220
240
260
280
300
320
340
360
380
400
420
440
460
480
500
m/z, amu

(*Z*)-2-(5-((1,3-di-*p*-tolyl-1H-pyrazol-4-yl)methylene)-4-oxo-2-thioxothiazolidin-3-yl)acetic acid (3a)

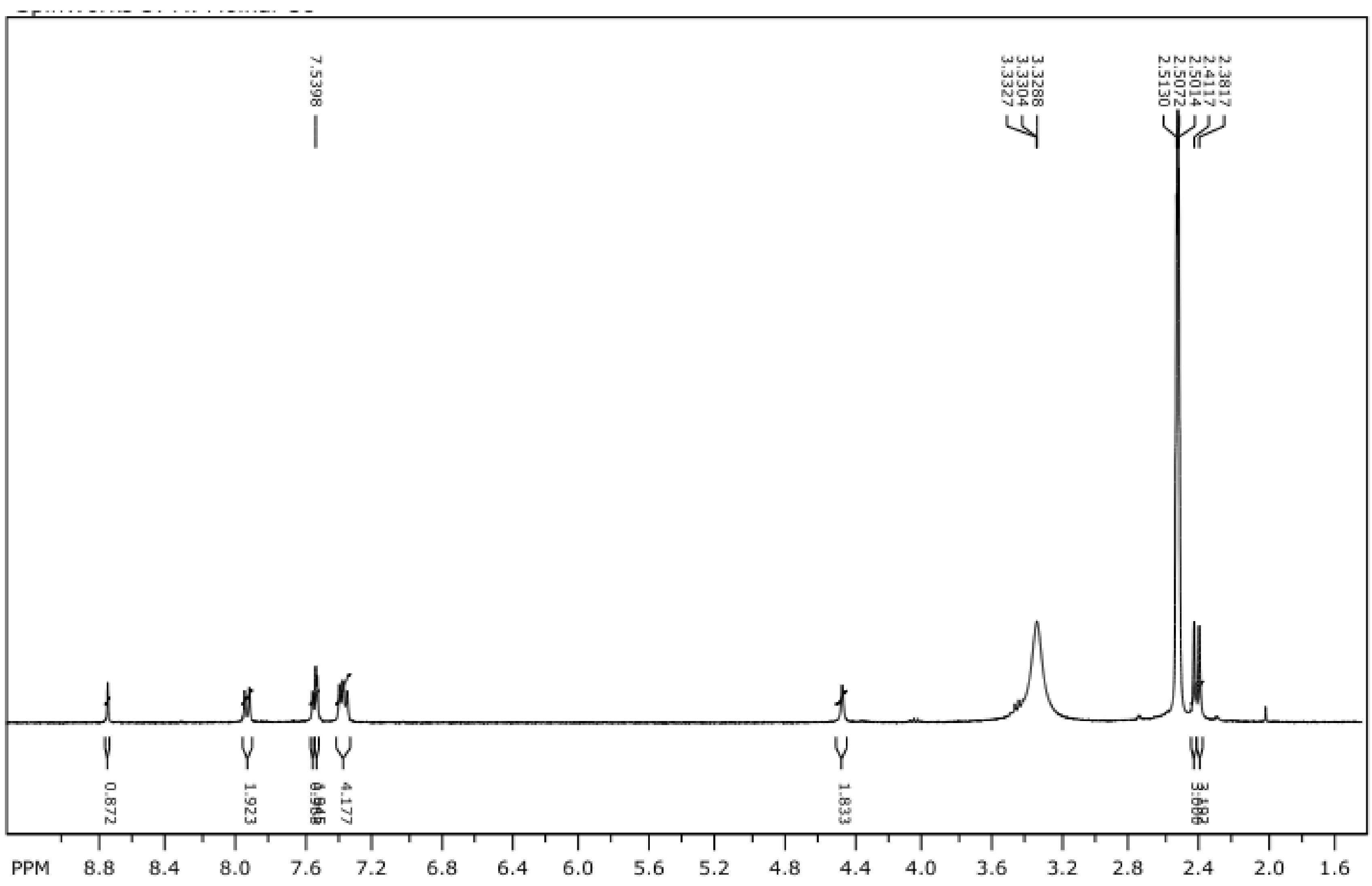

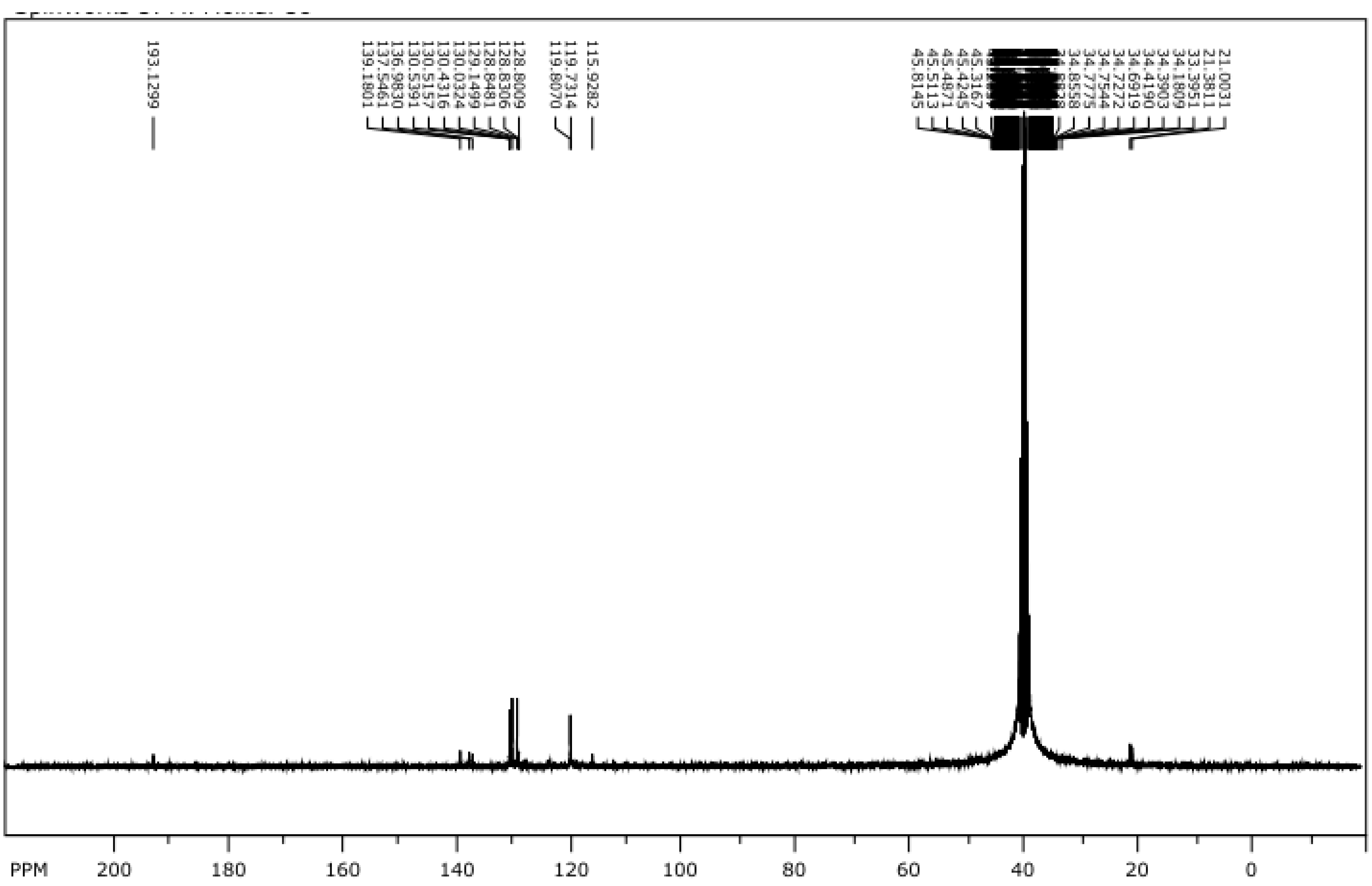
193.1299
139.1801
137.5461
136.9830
130.5391
130.5157
130.4316
130.0324
129.1499
128.8481
128.8306
128.8009
119.8070
119.7314
115.9282
45.8145
45.5113
45.4871
45.4245
45.3167
34.8558
34.7775
34.7544
34.7272
34.6919
34.4190
34.3903
34.1809
33.3951
21.3811
21.0031
PPM
200
180
160
140
120
100
80
60
40
20
0

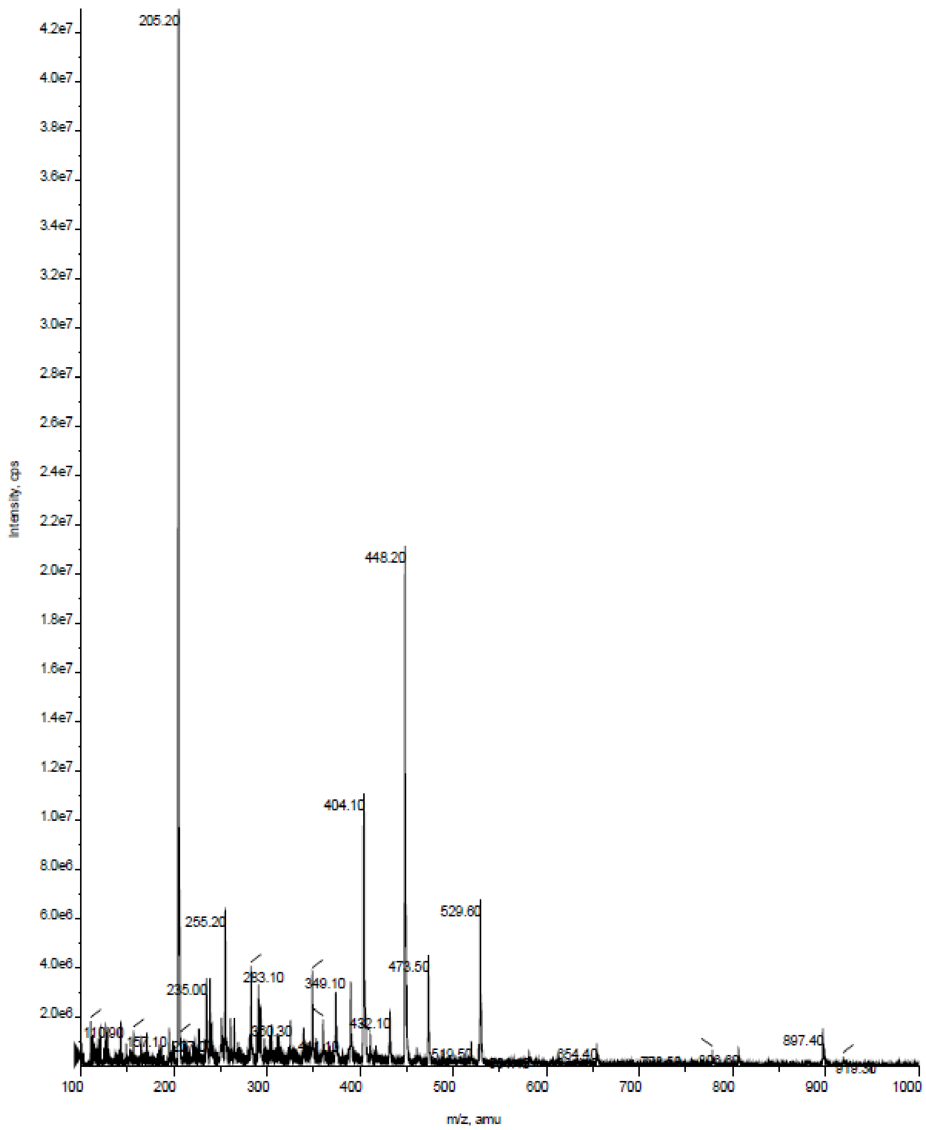
205.20
448.20
404.10
529.60
255.20
478.50
283.10
349.10
235.00
432.10
897.40
654.40
Intensity, cps
m/z, amu

(*Z*)-2-(5-((3-(4-fluorophenyl)-1-(*p*-tolyl)-1*H*-pyrazol-4-yl)methylene)-4-oxo-2-thioxothiazolidin-3-yl)acetic acid (3b)

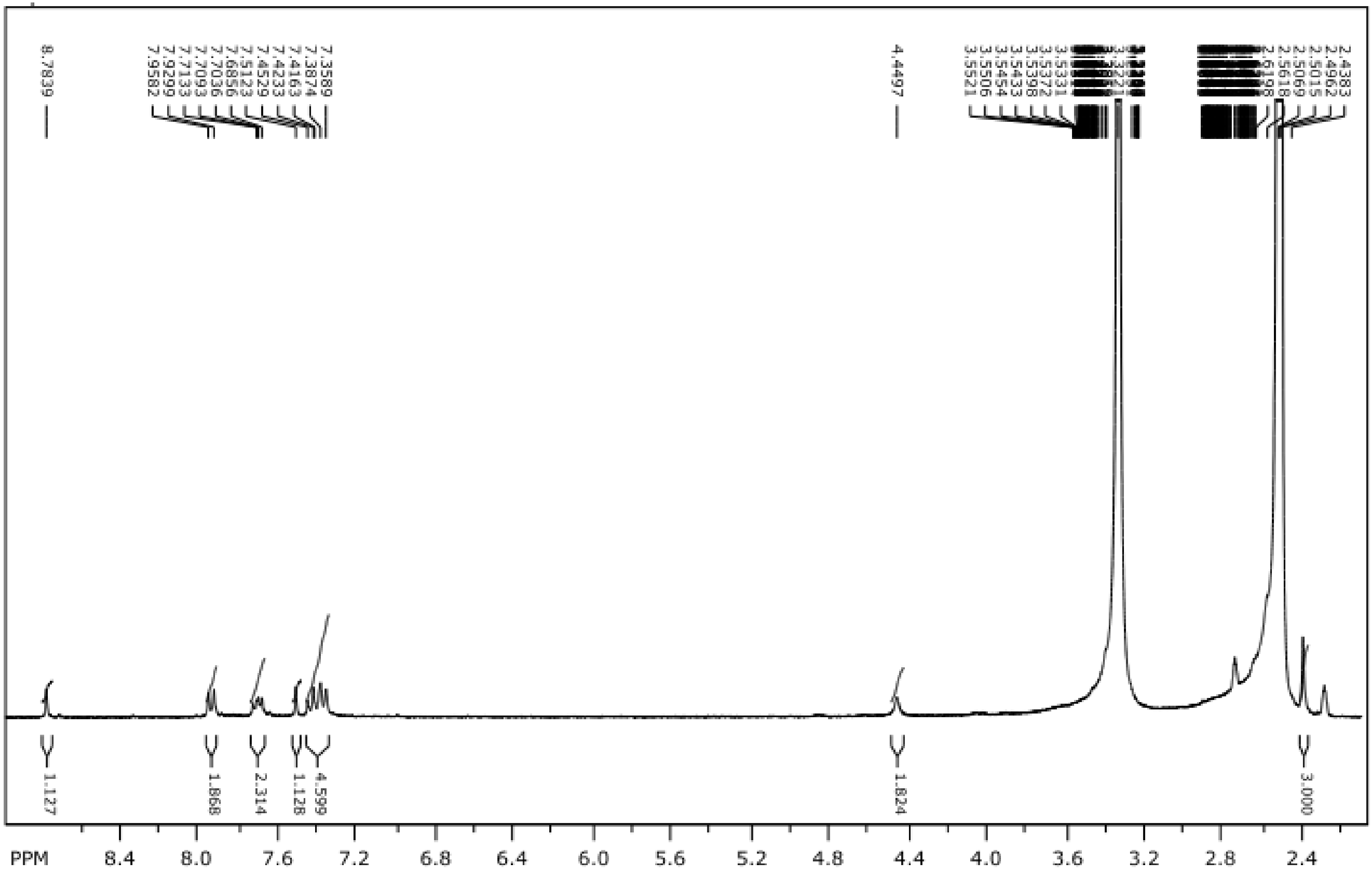

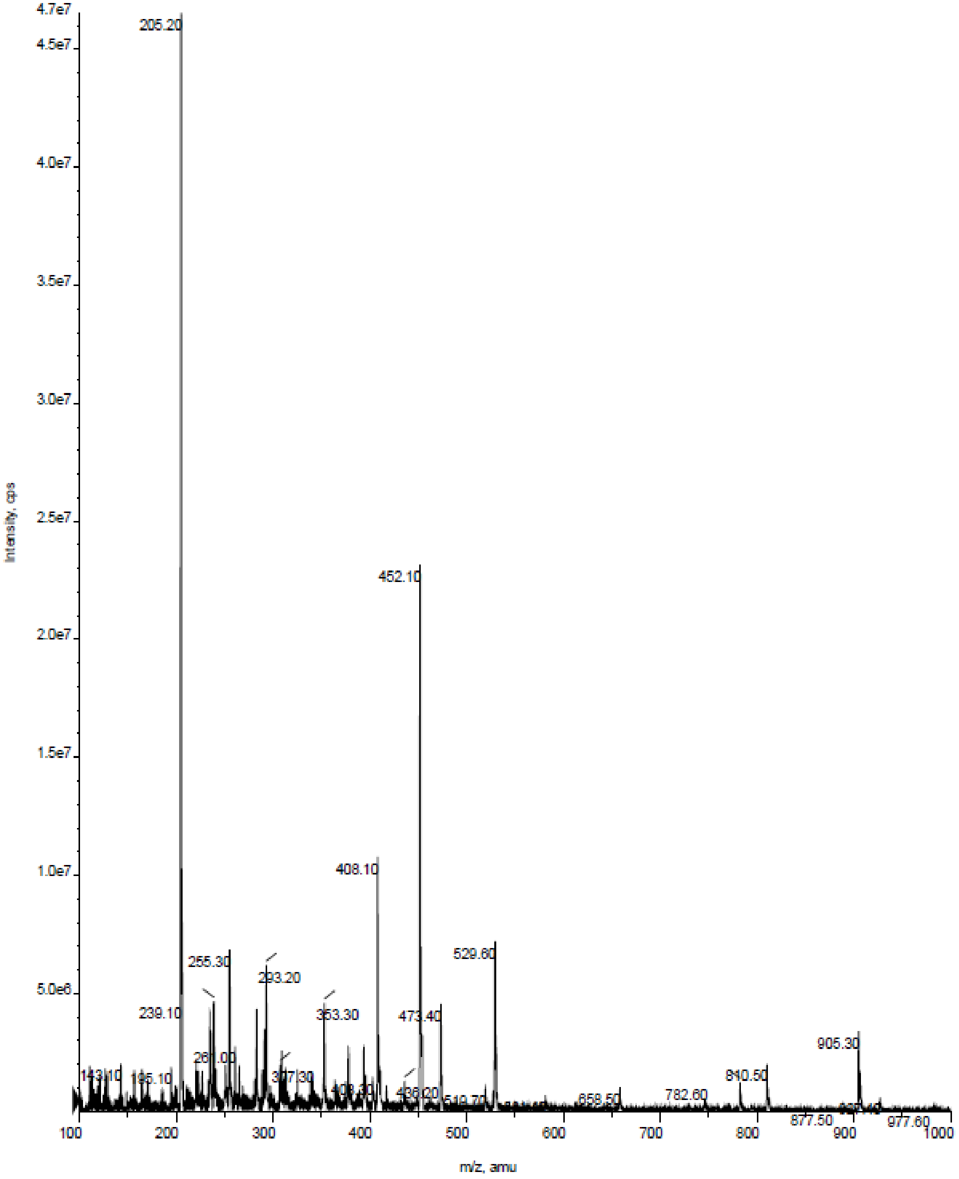

205.20
452.10
408.10
529.60
255.30
293.20
239.10
353.30
473.40
905.30
810.50
782.60
658.50
Intensity, cps
m/z, amu

(*Z*)-2-(5-((3-(4-methoxyphenyl)-1-(*p*-tolyl)-1*H*-pyrazol-4-yl)methylene)-4-oxo-2-thioxothiazolidin-3-yl)acetic acid (3c)

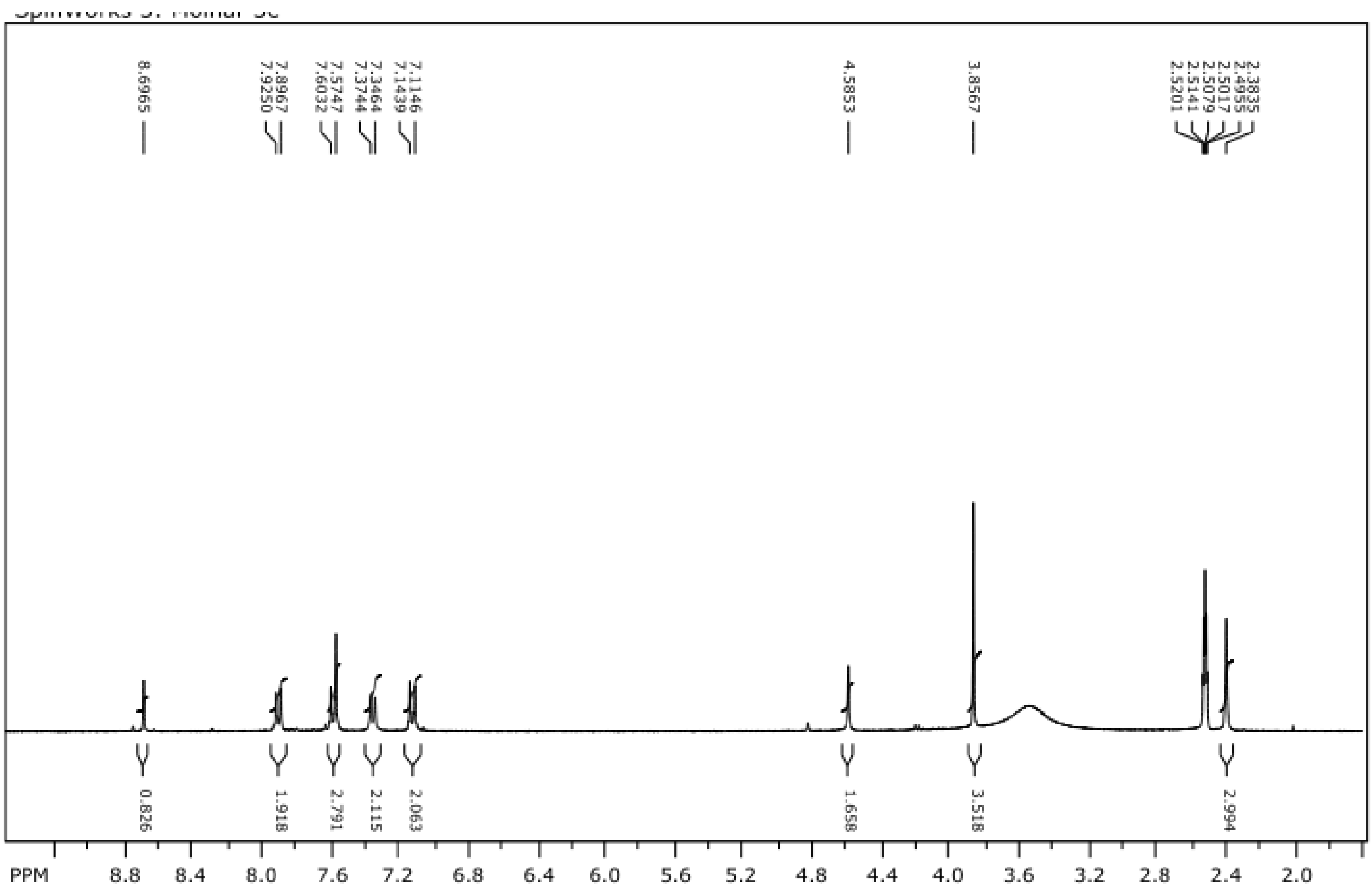

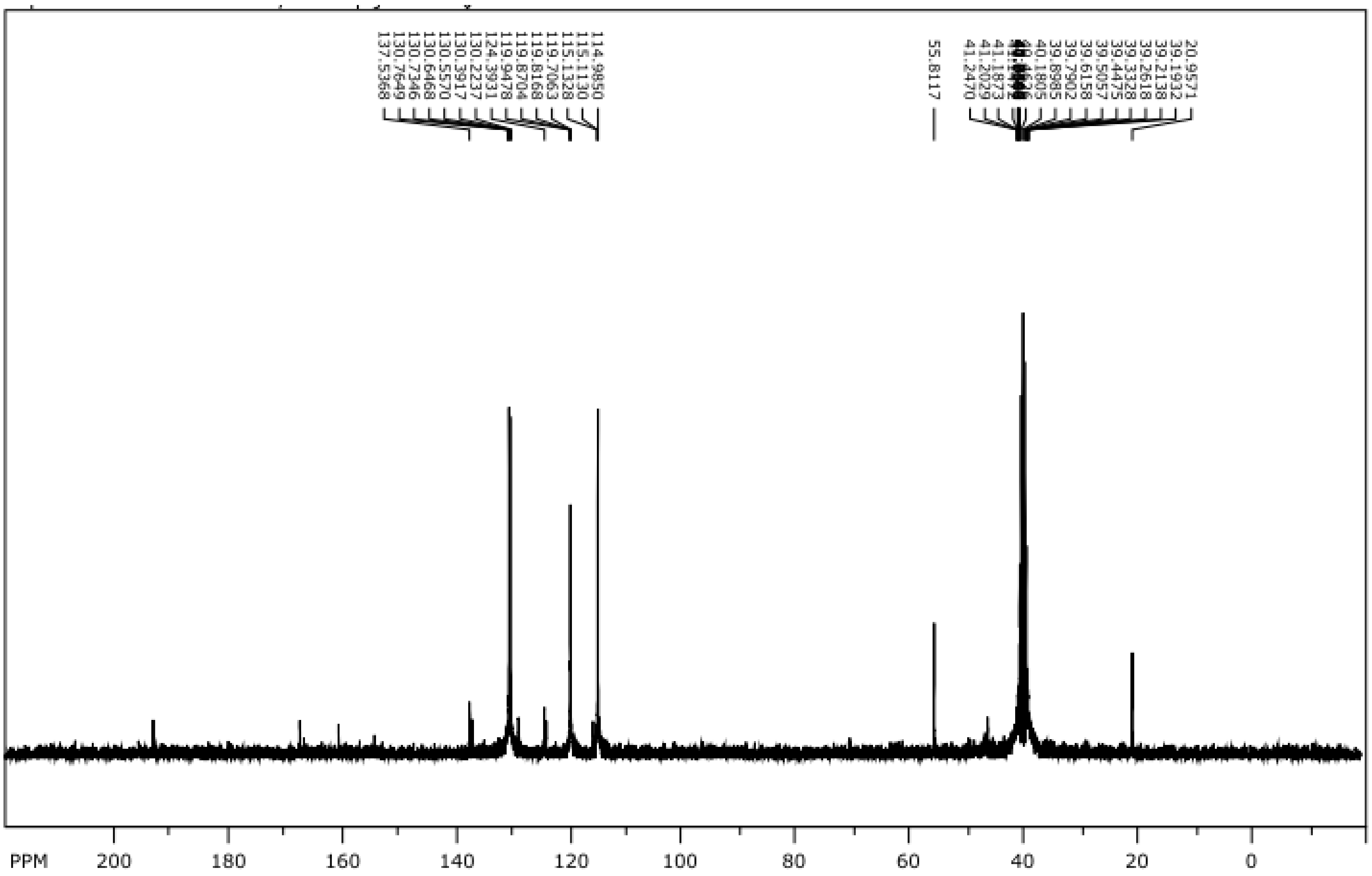
137.5368
130.7649
130.7346
130.6468
130.5570
130.3917
130.2237
124.3931
119.9478
119.8704
119.8168
119.7063
115.1328
115.1130
114.9850
55.8117
41.2470
41.2029
41.1873
40.1805
39.8985
39.7902
39.6158
39.5057
39.4475
39.3328
39.2618
39.2138
39.1932
20.9571
PPM
200
180
160
140
120
100
80
60
40
20
0

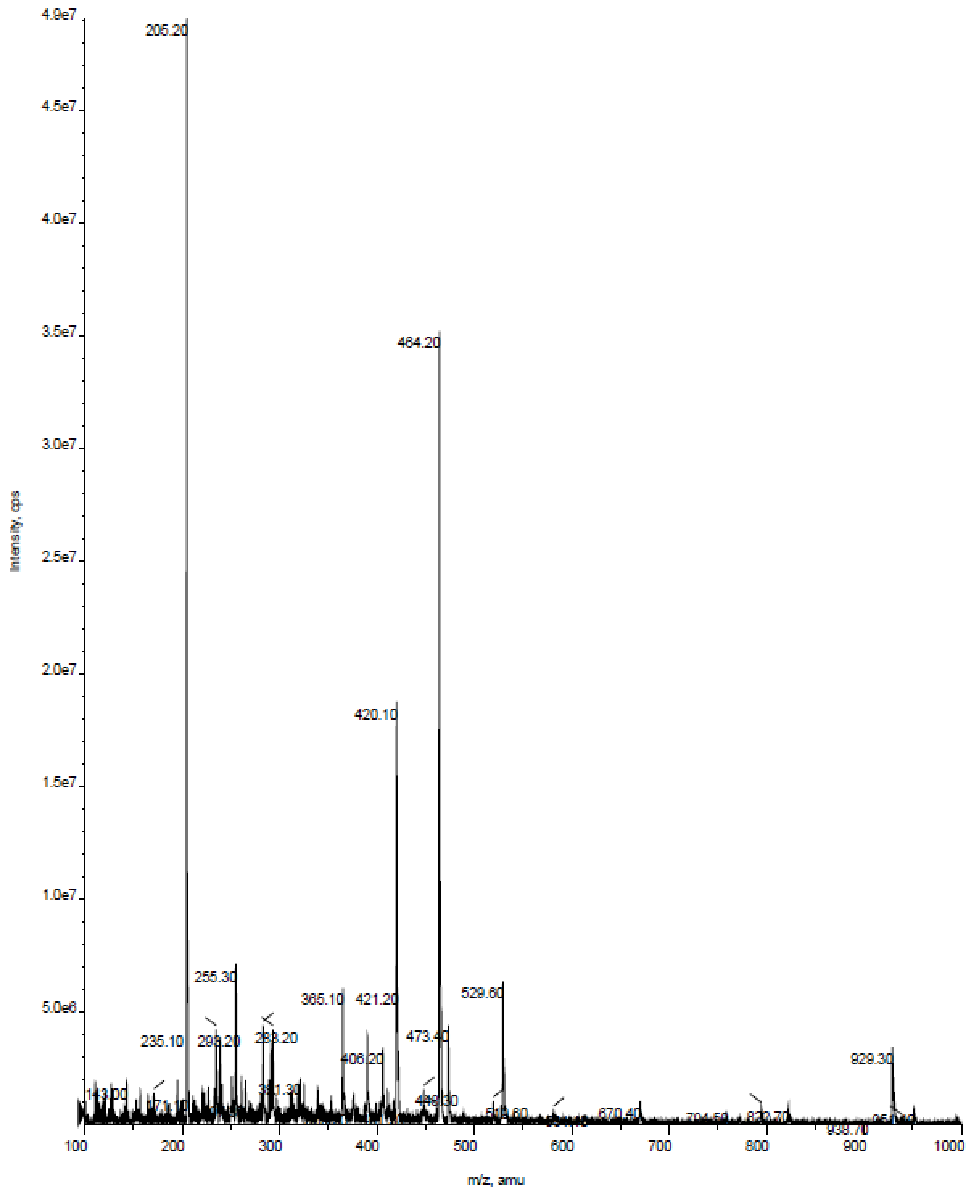

4.9e7
4.5e7
4.0e7
3.5e7
3.0e7
2.5e7
2.0e7
1.5e7
1.0e7
5.0e6
Intensity, cps
205.20
464.20
420.10
255.30
365.10
421.20
529.60
235.10
473.40
406.20
929.30
670.40
100
200
300
400
500
600
700
800
900
1000
m/z, amu

(*Z*)-2-(5-((3-(4-chlorophenyl)-1-(*p*-tolyl)-1*H*-pyrazol-4-yl)methylene)-4-oxo-2-thioxothiazolidin-3-yl)acetic acid (3d)

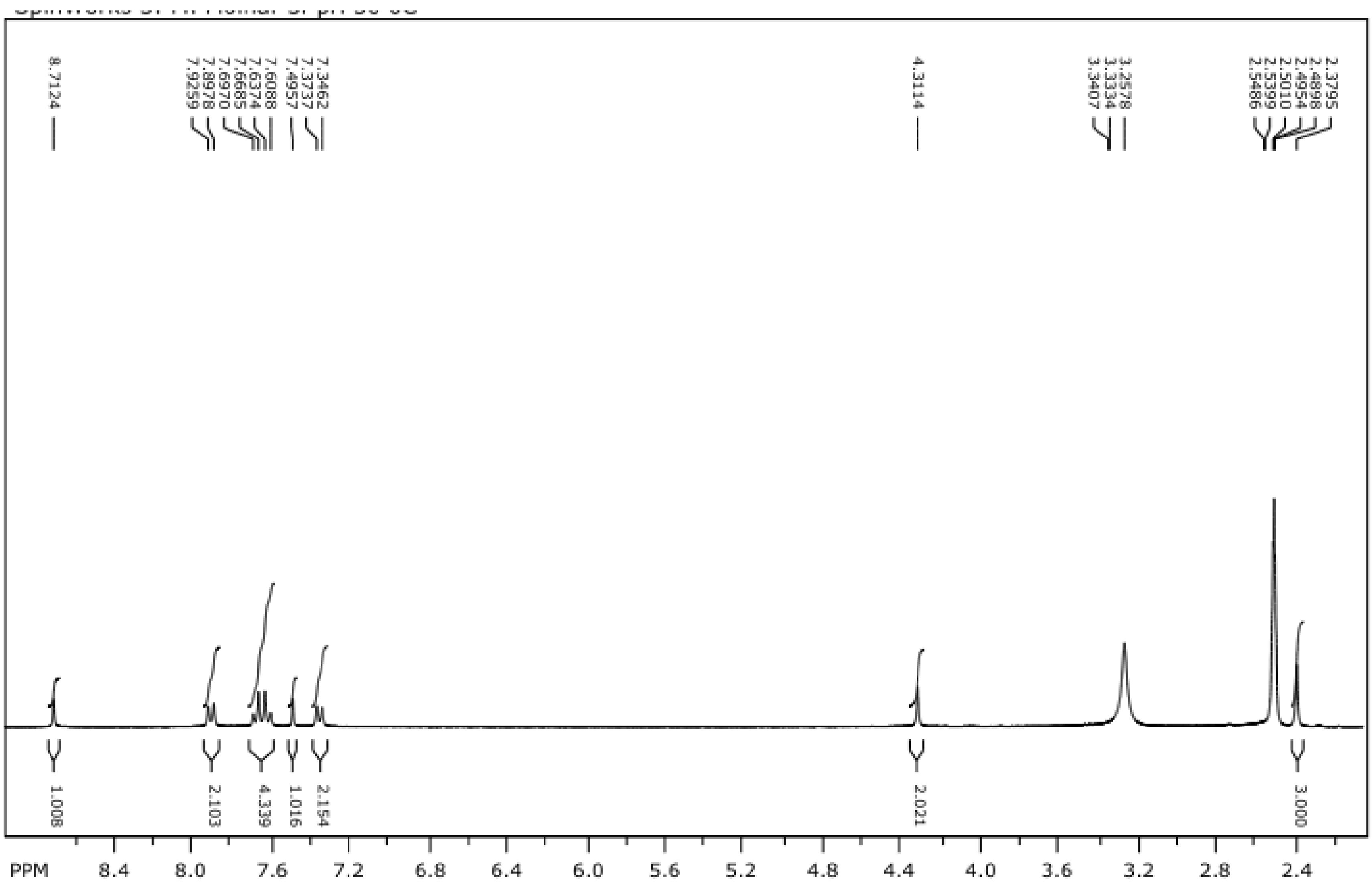

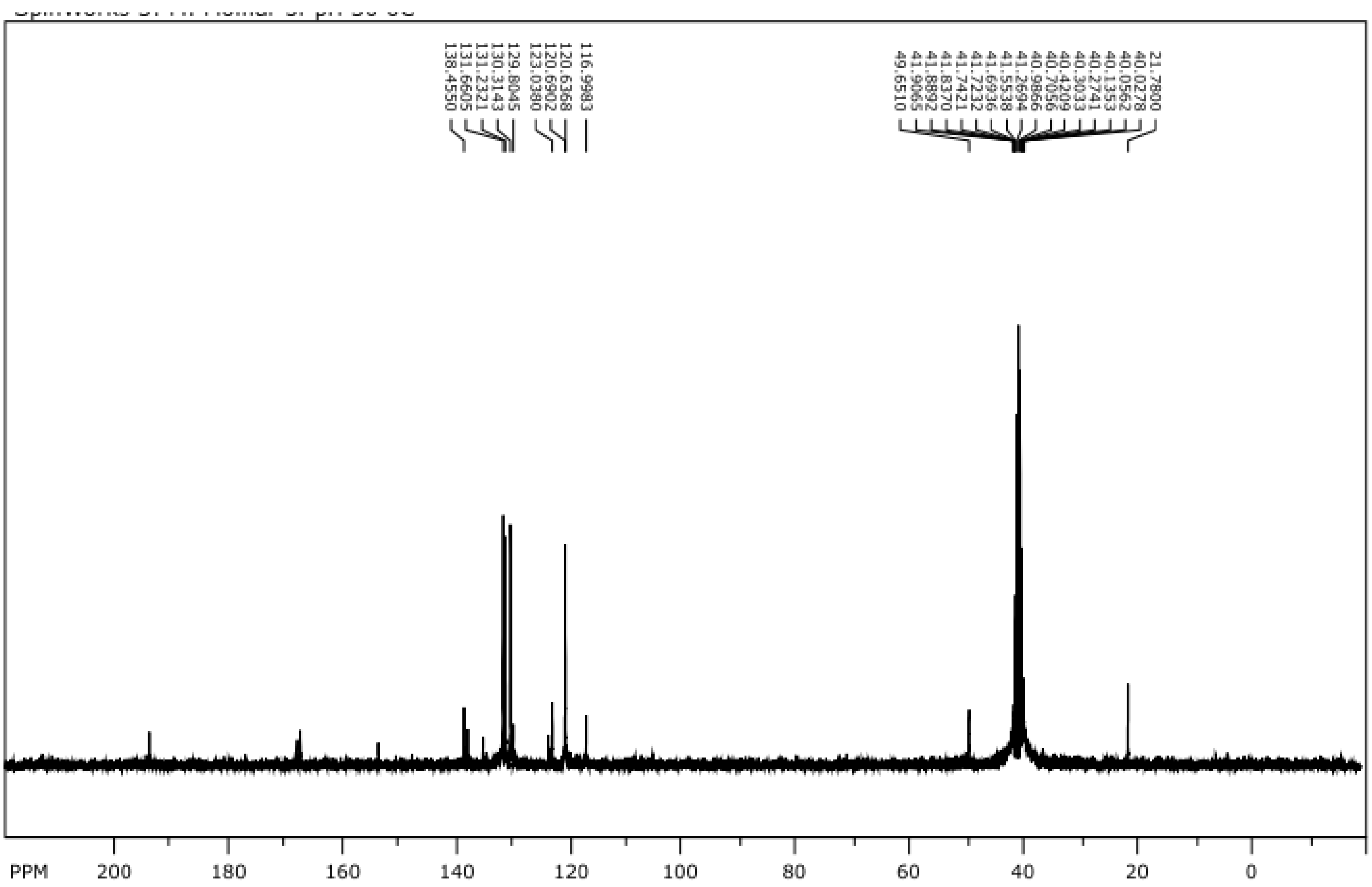

138.4550
131.6605
131.2321
130.3143
129.8045
123.0380
120.6902
120.6368
116.9983
49.6510
41.9065
41.8892
41.8370
41.7421
41.7232
41.6936
41.5538
41.2694
40.9866
40.7056
40.4209
40.3033
40.2741
40.1353
40.0562
40.0278
21.7800
PPM
200
180
160
140
120
100
80
60
40
20
0

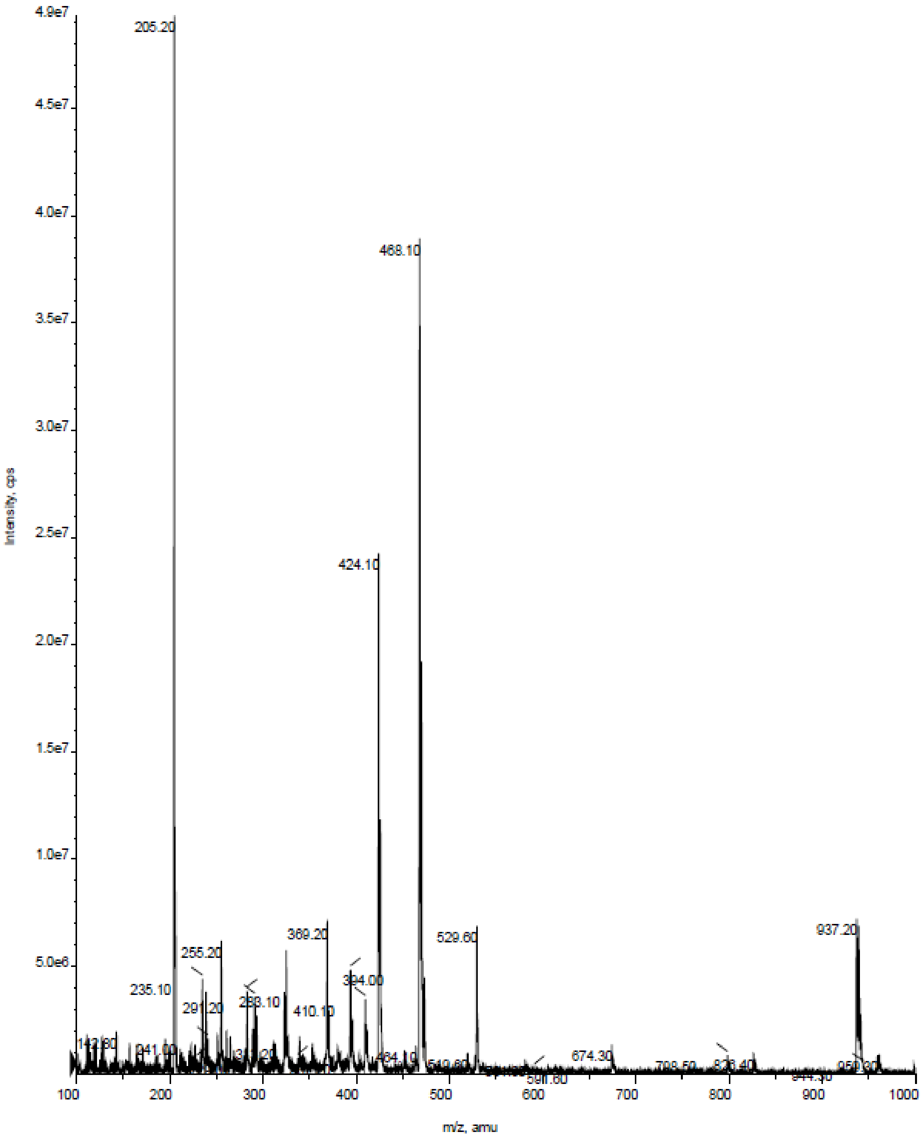

Intensity, cps
4.9e7
4.5e7
4.0e7
3.5e7
3.0e7
2.5e7
2.0e7
1.5e7
1.0e7
5.0e6
205.20
468.10
424.10
369.20
255.20
235.10
291.20
283.10
394.00
410.10
529.60
674.30
937.20
100
200
300
400
500
600
700
800
900
1000
m/z, amu

(*Z*)-2-(5-((3-(4-bromophenyl)-1-(*p*-tolyl)-1*H*-pyrazol-4-yl)methylene)-4-oxo-2-thioxothiazolidin-3-yl)acetic acid (3e)

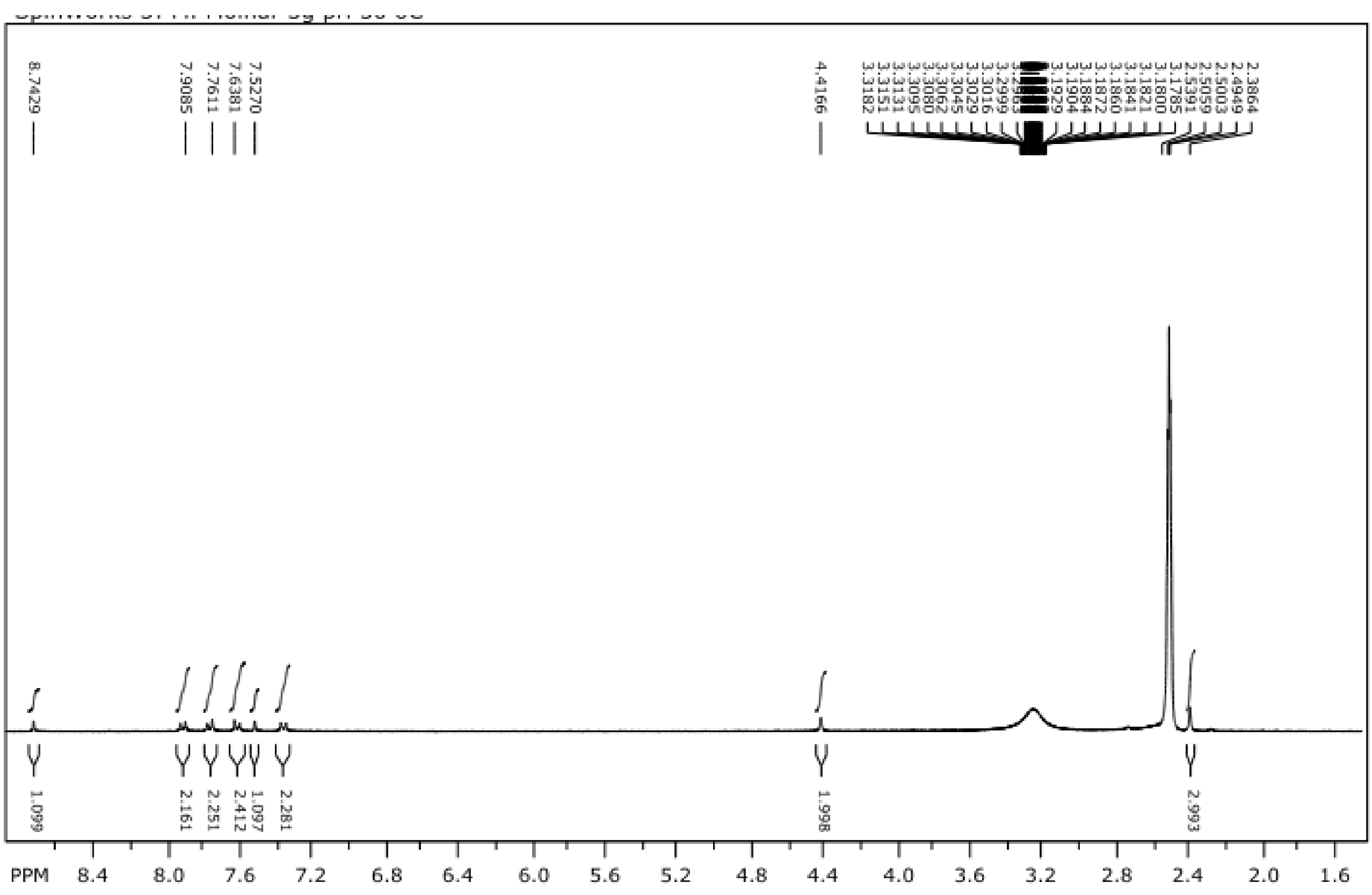

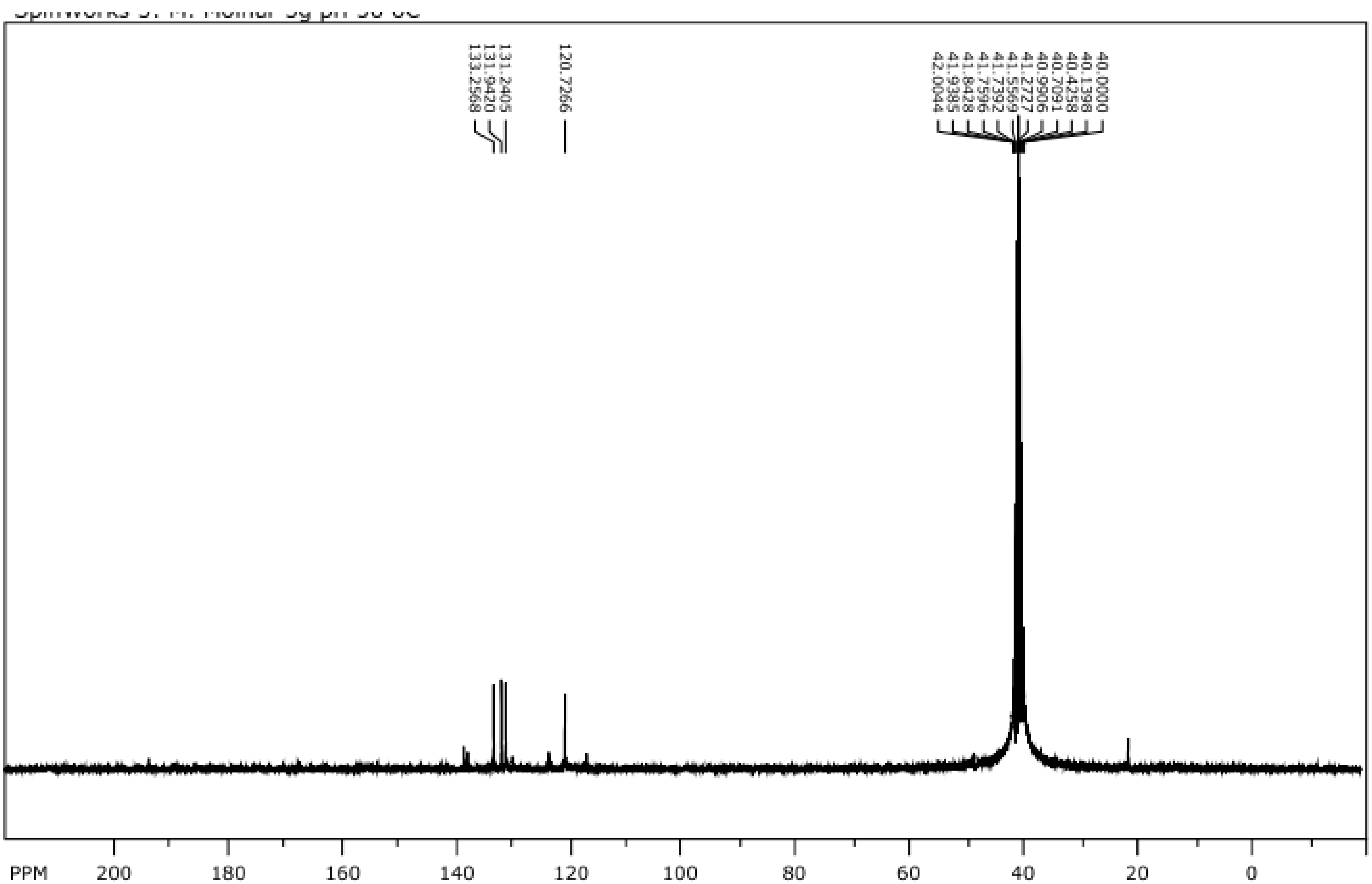
133.2568
131.9420
131.2405
120.7266
42.0044
41.9385
41.8428
41.7596
41.7392
41.5569
41.2727
40.9906
40.7091
40.4258
40.1398
40.0000
PPM
200
180
160
140
120
100
80
60
40
20
0

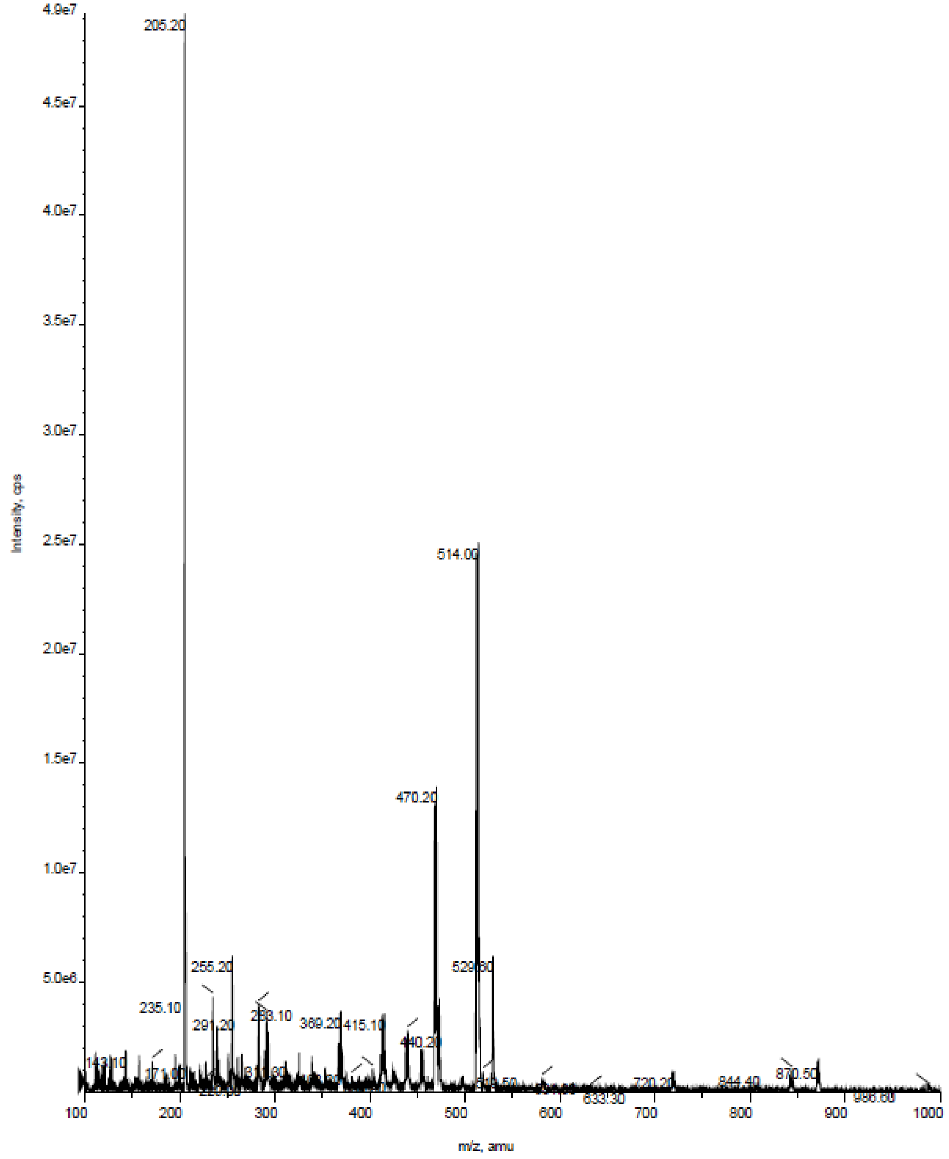

4.9e7
4.5e7
4.0e7
3.5e7
3.0e7
2.5e7
2.0e7
1.5e7
1.0e7
5.0e6
Intensity, cps
205.20
514.00
470.20
255.20
529.60
235.10
291.20
369.20
415.10
720.20
844.40
870.50
633.30
100
200
300
400
500
600
700
800
900
1000
m/z, amu

(*Z*)-2-(5-((3-(4-iodophenyl)-1-(*p*-tolyl)-1*H*-pyrazol-4-yl)methylene)-4-oxo-2-thioxothiazolidin-3-yl)acetic acid (3f)

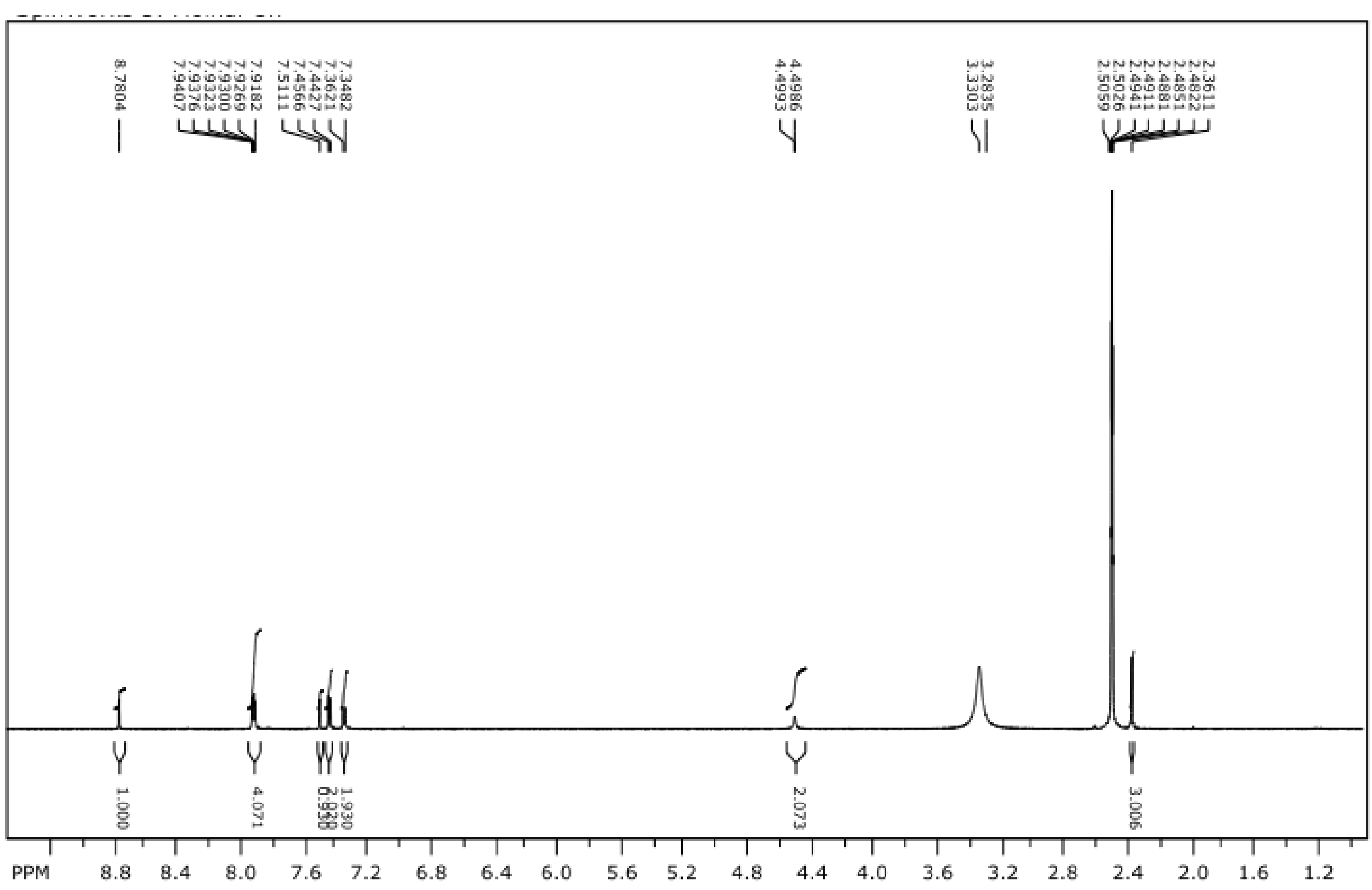

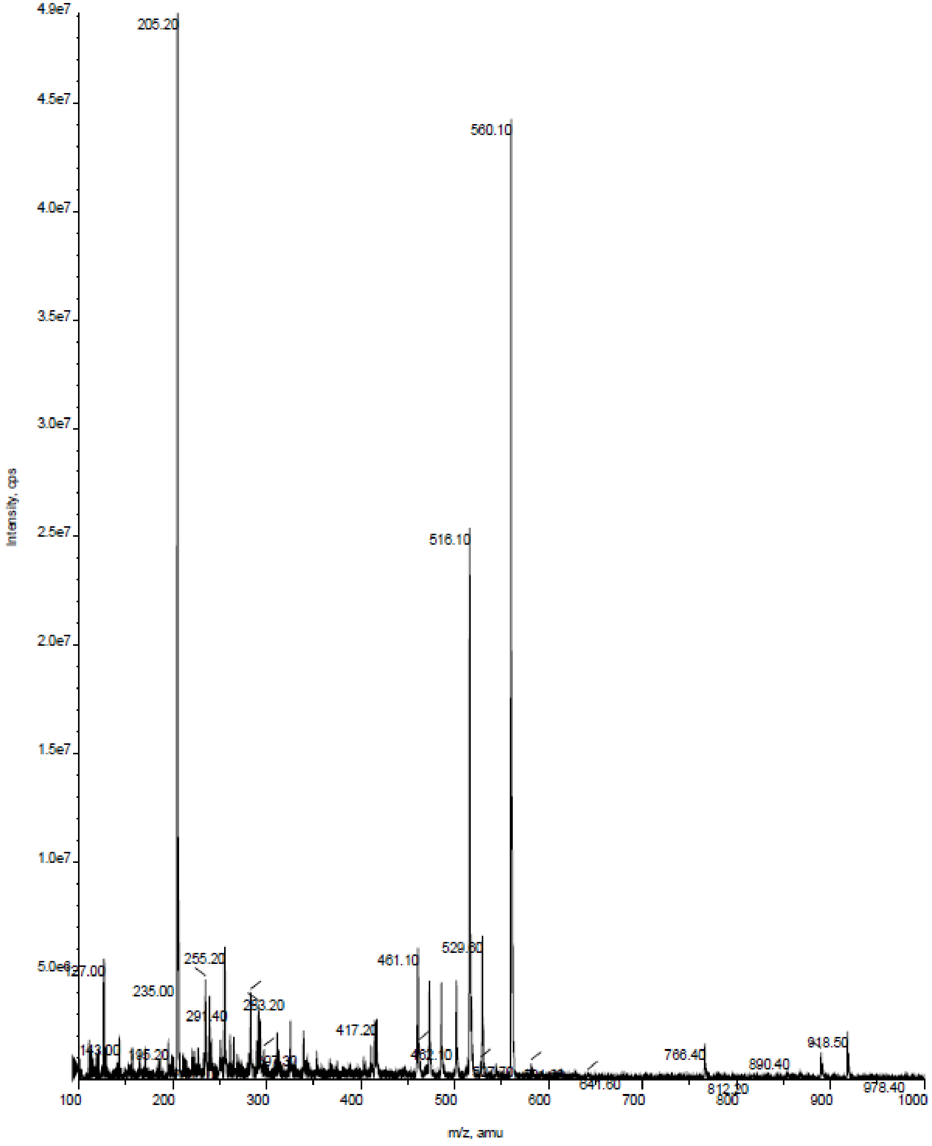

Intensity, cps
m/z, amu
205.20
560.10
516.10
461.10
529.60
255.20
235.00
127.00
291.40
283.20
417.20
143.00
766.40
890.40
918.50
978.40
